# Genome-wide temporal-spatial gene expression profiling of drought responsiveness in rice

**DOI:** 10.1186/1471-2164-12-149

**Published:** 2011-03-16

**Authors:** Di Wang, Yajiao Pan, Xiuqin Zhao, Linghua Zhu, Binying Fu, Zhikang Li

**Affiliations:** 1 Institute of Crop Sciences/National Key Facility for Crop Gene Resources and Genetic Improvement, Chinese Academy of Agricultural Sciences, Beijing 100081, China; 2 College of Life Sciences and Technology, Shenyang Agriculture University, Shenyang 110161, China; 3 International Rice Research Institute, DAPO Box 7777, Metro Manila, Philippines

## Abstract

**Background:**

Rice is highly sensitive to drought, and the effect of drought may vary with the different genotypes and development stages. Genome-wide gene expression profiling was used as the initial point to dissect molecular genetic mechanism of this complex trait and provide valuable information for the improvement of drought tolerance in rice. Affymetrix rice genome array containing 48,564 *japonica *and 1,260 *indica *sequences was used to analyze the gene expression pattern of rice exposed to drought stress. The transcriptome from leaf, root, and young panicle at three developmental stages was comparatively analyzed combined with bioinformatics exploring drought stress related *cis*-elements.

**Results:**

There were 5,284 genes detected to be differentially expressed under drought stress. Most of these genes were tissue- or stage-specific regulated by drought. The tissue-specific down-regulated genes showed distinct function categories as photosynthesis-related genes prevalent in leaf, and the genes involved in cell membrane biogenesis and cell wall modification over-presented in root and young panicle. In a drought environment, several genes, such as *GA2ox, SAP15*, and *Chitinase III*, were regulated in a reciprocal way in two tissues at the same development stage. A total of 261 transcription factor genes were detected to be differentially regulated by drought stress. Most of them were also regulated in a tissue- or stage-specific manner. A *cis*-element containing special CGCG box was identified to over-present in the upstream of 55 common induced genes, and it may be very important for rice plants responding to drought environment.

**Conclusions:**

Genome-wide gene expression profiling revealed that most of the drought differentially expressed genes (DEGs) were under temporal and spatial regulation, suggesting a crosstalk between various development cues and environmental stimuli. The identification of the differentially regulated DEGs, including TF genes and unique candidate *cis*-element for drought responsiveness, is a very useful resource for the functional dissection of the molecular mechanism in rice responding to environment stress.

## Background

Rice is sensitive to drought stress because it is acclimated to either rain-fed or fully irrigated fields. The effect of drought on rice plants considerably varies with genotypes, different developmental stages, and degree and duration of drought stress. Relatively, rice plants are less affected by water deficit at the seedling stage, although drought stress at the vegetative stage does result in reduced height, fewer tillers, and smaller leaf area. However, rice is highly sensitive to water deficit at the panicle initiation and booting stages [1,2].

Drought tolerance (DT) is a complex trait both genetically and physiologically. Developing DT varieties by breeding is the major strategy for reducing rice yield losses caused by drought. However, to date, few successes have been achieved in this effort because plant DT is a typical quantitative trait influenced by many different genes [3]. Genome-wide genetic analysis of DT has identified many genomic regions associated with drought tolerance or responsiveness [4]. Some secondary traits, such as root architecture and osmotic adjustment, have been identified to be related to drought response [5-7]. Few large and discrete DT quantitative trait loci (QTLs) have been identified and applied to marker-assisted selection of DT in rice. However, DT QTL mapping results can be most usefully applied to the identification of promising chromosome regions for the confirmation of functional candidate genes of drought tolerance.

Drought stress causes a wide range of physiological and biochemical responses in plants. These responses include reduced stomatal conductance and photosynthesis, and accumulation of osmolytes and proteins in cells [8]. A number of genes have been identified to be involved in drought response and tolerance, and their functions were confirmed by gene transfer, resulting in plant stress tolerance [9].

With the development of molecular technology and advancement in nanotechnology, DNA microarrays have been devised as a standard strategy for the global analysis of plant gene expression. Microarrays can simultaneously detect thousands of targets in a high throughput manner, and thus their use has enormously expanded to cover all kingdoms of living organisms. The availability of complete genome sequences and of huge EST collections allows the development of different microarray platforms in rice. Several biological processes and important traits of rice have been analyzed using cDNA microarray or whole genome array including salt-responsive genes by cDNA microarray analysis [10], genetic programs involved in pollination/fertilization and stress responses [11], spatial and temporal gene profiling of panicle development [12], comparative analysis of two rice genotypes under salt stress [13,14], drought and high salinity stress responsiveness gene profiling of different organs [15], and grain filing-related genes under high temperature [16]. DNA microarrays provide a high-throughput platform to screen thousands of genes simultaneously to identify gene alterations in the entire transcriptome across a variety of biological conditions. Combined with the whole rice genome sequences, gene function analysis, and comparative analysis of different genome sequences of crops, the entire genome transcriptional data will be the initial point for dissecting the molecular genetic mechanism of important complex agronomical traits in rice.

To achieve a more comprehensive understanding of the global spatial and temporal gene expression patterns of rice in response to drought stress, we performed a genome-wide gene profiling analysis using a unique drought tolerant rice line and an Affymetrix whole gene array set. Three tissues (i.e., leaf, young panicle, and root) at three developmental stages under drought stress and control conditions were used to profile their gene expression level. A temporal and spatial gene regulation pattern responsive to drought stress was primarily revealed in our study.

## Methods

### Plant materials, growth condition, and stress treatment

A drought tolerant rice line, DK151, an F_7 _line derived from a cross between two DT IR64 introgression lines (ILs), DGI 187 and DGI 74 (Additional file 1), was used in this study. Sterilized seeds of DK151 were allowed to germinate in distilled water for two days. The germinated seeds were then transferred to the seedling nursery. Rice plants at the four-leaf stage were transplanted in PVC tubes (size: 75 cm × 20 cm, each with a 20 cm hole from the bottom) with Turface (baked clay substrate mechanically broken into pieces with diameters of approximately 5 mm) (Applied Industrial Materials, Corp., Buffalo Grove, IL, USA) and watered with alternate applications of half-strength nutrient solution [17] and distilled water. The experiment design was a split-plot with three blocks. Each treatment was represented by six replicate pots with one plant per pot. Pots were randomized within the blocks. This experiment was processed in the green house of IRRI in the 2007 dry season.

To simulate drought stress, plants of DK151 were stressed by slowly draining the solution. The hole plug of each tube was removed. We applied the stress at three different stages: 4-tiller (tillering) stage, panicle elongation stage, and booting stage. Plants were stressed until the leaf became fully rolled at noon (measured leaf relative water content was 65%-75%). It took three and two days for drought stress to become apparent at the tillering and panicle elongation stages and at booting stage, respectively. Both leaf and root samples were collected for the first two stages, and leaves and young panicle samples were collected at the booting stage. Three biological replicates (each from individual plant) were prepared for microarray analysis. After collection, samples were snap frozen in liquid nitrogen and kept in a -80°C freezer for total RNA extraction.

### Total RNA isolation, qualification, and processing for microarray analysis

RNA preparation was conducted following the instructions of Affymetrix (Affymetrix GeneChip Expression Analysis Technical Manual, Affymetrix). Briefly total RNA was extracted from liquid nitrogen frozen sample using TRIZOL reagent according to the instruction, and then purified and concentrated using RNeasy MinElute Cleanup kit (Qiagen 74204, Germany) and an on-column DNase treatment as recommended by Affymetrix. The following steps were then processed in CapitalBio Corporation, Beijing. Total RNA of 2 μg was used for synthesizing ds cDNA. Biotin-tagged cRNA was generated from an in vitro transcription reaction using MessageAmp™II aRNA Amplification Kit and then fragmented into 35-200 bases in length according to the Affymetrix protocol. The resulting cRNA was then hybridized to the Affymetrix rice genome array (Affymetrix) containing 48,564 *japonica *and 1,260 *indica *sequences. Hybridization was processed at 45°C, with rotation for 16 h (Affymetrix GeneChip Hybridization Oven 640). Chips were then washed and stained in the Affymetrix Fluidics Station 450 and then scanned using the Affymetrix Gene Chip Scanner 3000. All experiment steps were conducted in CapitalBio Corporation in Beijing.

### Array data analysis

GeneChip Operating Software (GCOS1.4) was used to analyze the hybridization data. The scanned images were first examined by visual inspection and then processed to generate raw data saved as CEL files using the default setting of GCOS1.4. We used dChip software to perform invariant-set normalization according to the dChip user's manual. The whole set of original microarray data has been deposited in NCBI's Gene Expression Omnibus and can be freely accessed through GEO Series number GSE26280.

For comparison analysis, two classes of unpaired methods in the Significant Analysis of Microarray (SAM) software were applied to identify differentially expressed genes (DEGs) between the drought stress sample and the control sample. As there is no fixed standard threshold between significant and non-significant differential gene expressions, we identified the DEGs using the empirical criteria of more than five-fold change and significant *t *test of *P *value less than 0.05 based on three independent biological replicates. The DEGs were performed in complete linkage hierarchical clustering analysis using the TIGR MeV 4.2 software http://www.tm4.org.

### Functional classification and prediction of cis-acting regulatory elements for DEGs

The putative function of each DEG corresponding to the probe set on the chip was predicted by Affymetrix annotation combined with TIGR definition and NCBI database. GO analysis was performed by a Molecule Annotation System (MAS, http://bioinfo.capitalbio.com/mas/). Significance analysis of GO was performed by the gene enrichment based on hypergeometric distribution finished by Fisher or Chi-sequare test.

A Weeder program [18] was used to predict the *cis *regulatory elements for the DEGs set under drought stress. The gene sequences including the upstream were downloaded from the TIGR Web site for all selected DEGs. The shared motifs 6, 8, 10, and 12 bp in length (allowing one mismatch) and the known ABRE motifs were located in the promoter regions (-10 to -1000 bp upstream of the start codon) and compared with the promoter regions of all control genes (*P*-value ≤ 0.05).

### RT-PCR confirmation of candidate genes related to drought responsiveness

Several genes with special tissue-specific or stage-specific DEGs were selected to confirm the expression level of microarray results using RT-PCR. The sequences corresponding to the genes were obtained from the rice genome sequences database (TIGR). The sequences of exons from genes were used to design the RT-PCR primers using the Primer 3 software http://frodo.wi.mit.edu/. An *Actin *gene was used as internal control. RT-PCR-amplified products were sequenced, and 100% homology to the target sequences was confirmed. PCR reaction was performed using the same RNA samples used for the microarray analysis. The first-strand cDNA was obtained from 1 μg of total RNA in a 50 μl reaction mixture, and 1 μl of synthesized cDNA was used as template for PCR reaction (94°C for 2 min and then 26 cycles of 30 s at 94°C, 30 s at 52°C, and 30 s at 72°C, followed by 72°C for 2 min). All assays were performed in triplicate. After gel electrophoresis, we measured the intensity of each band and normalized the data using the beta actin bands of each well, respectively.

## Results and Discussion

### Drought stress treatment and root sampling

The objective of this study is to genome-wide profile the expression of genes in rice in a drought environment. To simulate natural growth conditions, rice plants were cultured in the glasshouse. Turface in the growth tubes was used to support plants for the easy and quick collection of root samples under drought treatment and control conditions. This simulated drought strategy is quite different from that previously used, such as hydroponic cultured in PEG solution [19] or air-drying [20] mixed clay soil with sand [21]. Plant roots play a vital role in water and mineral acquisition, and they are more sensitive to the change in soil environment. Under drought stress, roots can continue to grow and send the stress signal to the shoot. However, knowledge of gene expression and metabolic regulation in the root is limited because of the difficulty of the root sampling. There is no study yet on rice root under drought stress environment because of the difficulty of root sampling. Thus, our study offers the first comprehensive genome-wide gene profiling of drought responsiveness in the whole rice plant.

### Identification and classification of drought-induced DEGs in different tissues at three developmental stages of rice

Affymetrix whole rice genome array was used to profile the rice gene expression under drought and control conditions in this study. The array contains 49,824 rice genome genes/transcripts (48,564 and 1,260 for *japonica *and *indica*, respectively). Among these, 18,976 (38.1%) to 23,068 (46.4%) genes were detected to be expressed in each of the six samples under stressed or control environments (Additional file 2). We found most genes to be detectable in young panicle under control (46.5%) and drought stress (46.1%), and the least genes to be detectable in the leaf under control (38.1%-39.2%) and drought conditions (39.2%-41.9%).

To identify statistically significant DEGs under drought stress, we used the combined criteria of five-fold or more change and significant *t *test of *P *value of less than 0.05 based on three biological replicates. We detected the expression of 5,284 DEGs (10.6%) in at least one of the six samples. A total of 1154, 878, 1114, 3283, 905, and 998 DEGs were found up- or down-regulated by drought in the samples of root at the tillering stage (TR), leaf at tillering stage (TL), root at panicle elongation stage (PR), leaf at panicle elongation (PL), young panicle sample at booting stage (BP), and leaf at booting stages (BL), respectively (Table 1). In particular, most DEGs were identified in the leaf tissue during panicle elongation, the critical stage at which rice plants transit from the vegetative stage to the reproductive one, including 1316 drought-induced genes and 1967 drought-repressed genes, respectively.

**Table 1:** Summary of the genes up or down-regulated by drought stress in each tissue at different developmental stages

Tissue	Up Regulated Genes	Down Regulated Genes	Sub-Total
Root at tillering stage	391	763	1154
Leave at tillering stage	320	558	878
Root at panicle elongaiton stage	581	533	1114
Leave at panicle elongation stage	1316	1967	3283
Panicle at booting stage	351	554	905
Leave at booting stage	643	355	998

***Note***: The DEGs were identified using the empirical criterion of more than five-fold change and significant *t *tests of *P *< 0.05 based on the three independent biological replicates.

**Figure 1:**
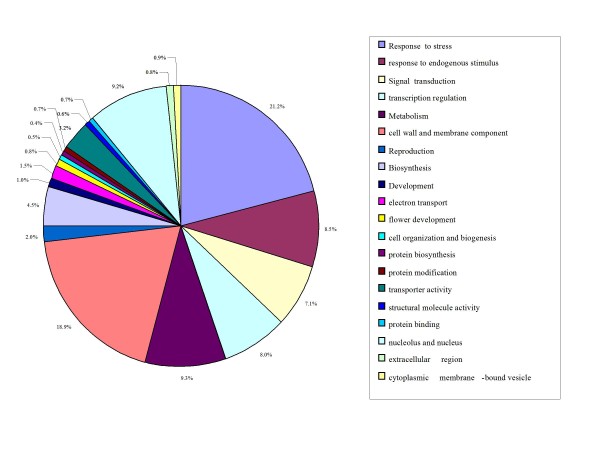
**Function classification of all 5283 DEGs in at least one sample under drought stress**.

In Figure 1, the detected 5,284 DEGs with known and putative function covered virtually all functional categories. The predominant DEGs were involved in response to stress including biotic and abiotic stimuli (21.2%, *q *= 4.33E-137), response to endogenous stimulus (8.5%, *q *= 2.43E-175), transcription regulation (8.0%, *q *= 0.005), metabolism such as lipid and carbohydrate metabolism (9.3%, *q *= 0.63), signal transcription (7.1%, *q *= 6.49E-102), and cell wall and membrane component (18.9%, *q *= 3.1E-70).

To investigate the similarities and differences of all DEGs among samples according to transcription levels, hierarchical complete linkage analysis was performed using TIGR MeV (Version 4.3, http://www.tm4.org/mev.html). Results show that the DEGs can be classified into several groups according to the expression pattern: the first group was the up-regulated gene set in at least one tissue; the second group was down-regulated gene set in at least one tissue; and the third group indicates the genes were specifically induced or repressed in one unique tissue (i.e., leaf, root, or panicle) under drought stress (Additional file 3).

To confirm the microarray profiling data, 24 genes were selected for semi-quantitative RT-PCR analysis. The gene specific primers are listed in Additional file 4. Among these genes, there were 4, 14, and 6 with reciprocal action under drought from two tissues at the tillering stage, panicle elongation stage, and booting stage, respectively. There is good correlation between RT-PCR and microarray data in general. The expression pattern of several genes failed to confirm the microarray data because semi-quantitative RT-PCR examined the expression patterns of individual genes, as profiled by a single peak in the melting curve analysis. In the microarray analysis, no distinction was made between gene family members (Additional file 5).

### Comparison of DEGs in different tissues at three development stages

**Figure 2:**
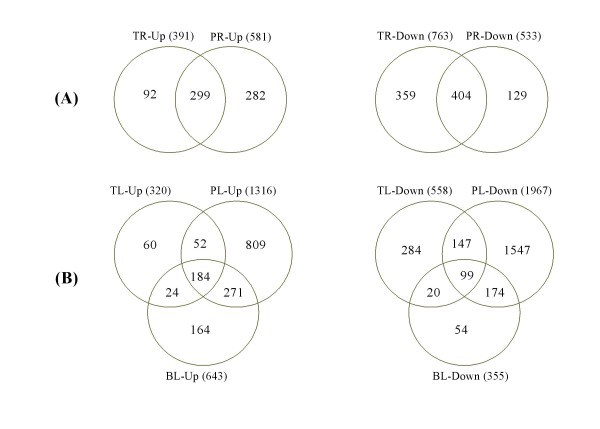
**Venn diagram of up- and down-regulated genes under drought stress in developmental stages**. **A**. In roots between the tillering stage (TR) and the panicle elongation stage (PR); **B**. In leaves in the tillering stage (TL), panicle elongation stage (PL), and booting stage (BL).

We first compared the DEGs in the root or leaf tissues at different developmental stages. Venn diagram results indicate that a number of DEGs overlapped between leaves or between roots at different developmental stages. Figure 2A shows the comparison results of up- or down-regulated genes in roots under drought stress at the tillering and panicle elongation stages. There were 299 and 404 genes commonly up- and down-regulated in roots by drought. A total of 92, 282 genes were induced and 359, 129 genes were repressed by drought exclusively in roots at the tillering and panicle elongation stages, respectively. Correspondingly, there was only a small portion of genes commonly regulated in leaves; 184 and 99 genes were detected to be commonly induced and repressed in all leaves at the three stages. Moreover, 60, 809, and 164 genes were induced, and 284, 1547, and 54 genes were peculiarly repressed by drought in leaves at the tillering, panicle elongation, and booting stages, respectively (Figure 2B).

Stage-specific DEGs were also analyzed. There were only 121, 279, and 167 genes shared, which were up-regulated by drought. A total of 58, 81, and 19 genes were commonly down-regulated between two tissues at the tillering, panicle elongation, and booting stages, respectively (Additional file 6). These results indicate that drought responsive genes in rice are highly dependent on the developmental stage and tissue type.

DEGs in all samples were further compared to screen the common up- or down-regulated genes by drought. We found a small set of genes commonly induced by drought stress in all tissues (Additional file 7). After removing the overlapping probes with the same gene accession number, 55 genes were identified (see Additional file 8), many of which also responded to other stresses, such as extreme temperature and salt [13,14,16]. There was a significant subset of common up-regulated genes (*n *= 7) identified as LEA proteins, four temperature-induced proteins (i.e., hsp70, lt101, cor14b, and another cold regulated protein), four dehydrin family proteins, and two protein phosphatase 2C family proteins, which were identified in a common induced gene set, involved in abiotic stress responsiveness. The others are related to amino acid and nucleotide metabolism, putative proteins, and proteins with unknown function.

Surprisingly, there was no gene detected as commonly repressed in all samples by the criterion of the five-fold change in our experiment. When the two-fold change criterion was adopted, only 20 genes were identified as commonly down-regulated in all tissues under drought stress (Additional file 9). Majority (12/20) of these sets of down-regulated genes were functionally classified into categories of cell wall extension or membrane metabolism, such as expansion, tubulin, transmembrane, and enzymes for the reorganization, division, and biosynthesis of cell wall.

### Unique functional categories of tissue-specific DEGs corresponding to their biological function

**Figure 3:**
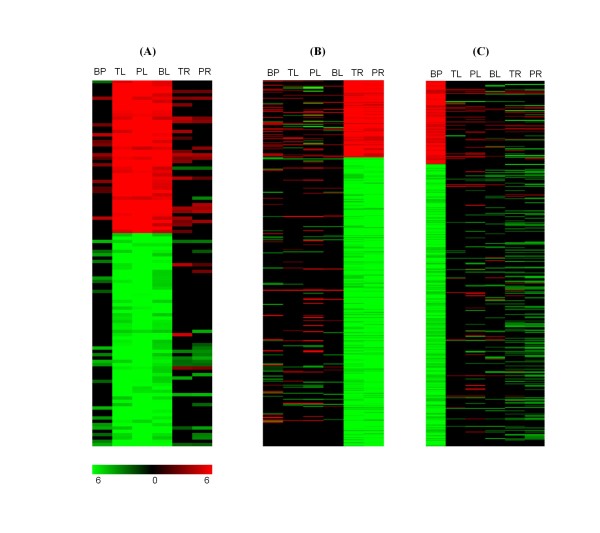
**Heat map view of the leaf- (A), root- (B), and panicle-specific (C) DEGs clusters**. The 110 genes in the leaves at all three stages (i.e., TL, PL, and BL), 363 genes in the roots at the tillering and panicle elongation stages (TL and PL), 448 genes at the young panicle at booting stage (BP) were detected to be specifically induced or repressed under drought stress.

To identify tissue- or stage-specific regulated genes from the 5,284 DEGs, the five-fold change in one surveyed target (tissue or stage) was required. However, this did not hold true in others. Tissue-specific DEGs were screened out using the following strategy: the genes with more than five-fold change only in the leaf tissues at the three development stages under drought were identified as the leaf specifically up- or down-regulated gene set; the specific DEGs in roots and panicle were also identified as those in leaves. Stage-specific DEGs were identified as genes with more than five-fold change in two tissues at one development stage only. After removing the redundancies of the probes, a total of 110, 363, and 448 tissue-specific DEGs were identified in leaf, root, and panicle, respectively (Figure 3, Table 2). More than three-quarters of the root-specific (286/363) and panicle-specific (346/448) DEGs were detected to be down-regulated under drought stress. We also detected 58 and 13 genes specifically induced by drought at the panicle elongation and booting stages, respectively, whereas two down-regulated specific genes were found at the panicle elongation stages. We could only identify three down-regulated genes under drought at the tillering stage, at which the rice plant is in the vegetative growth phase (Table 2).

**Table 2:** List of tissue- and stage-specific up- and down-regulated genes under drought stress

Tissue/Stage	Up-regulated	Down-regulated	Subtotal
Leaf	46	64	110
Root	77	286	363
Panicle	102	346	448
Tillering stage	0	3	3
Panicle elongation stage	58	2	60
Booting stage	13	0	13

**Figure 4:**
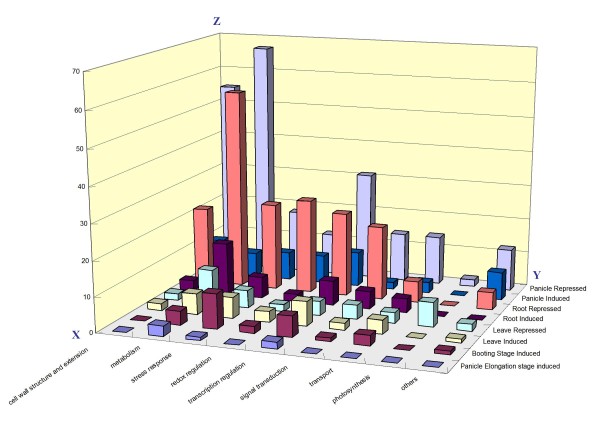
**Distribution diagram of the tissue- and stage-specific induced or repressed genes**. The x axis indicates the DEGs induced or repressed in specific tissue or stage. The y axis represents the function category, such as cell wall structure and extension, metabolism, stress response, redox regulation, transcription regulation, signal transduction, transport, photosynthesis, etc. The z axis indicates the total number of the specific DEGs with a certain function category in tissue or stage.

To classify functionally the tissue- and stage-specific DEGs, gene ontology analysis was performed. Figure 4 shows some unique features for the individual tissue-specific DEGs, especially for the down-regulated gene sets. However, we could not find any unique function cluster for the stage-specific DEGs.

In leaves, the dominant categories of these specific DEGs were involved in metabolism, stress response, and transcription regulation, except that the products of one-third of the specific genes were putative proteins with unknown function. The 47 genes were determined to be specifically induced by drought in all leaves. Among them, four phytohormone-related genes were identified: ACC synthase (Os01g0192900), SDR protein (Os07g0664600) involved in ABA biosynthesis [22], IAA26 (Os01g0741900) responsive to IAA, and Auxin hydrogen symporter (Os09g31478) involved in Auxin polar transport (Additional file 10). Seven photosynthesis-related genes were uniquely down-regulated in all leaves in a drought environment (Additional file 11): chlorophyll a/b-binding protein CP24, photosystem I reaction center subunit V, protochlorophyllide reductase A, peptidyl-prolyl cis-trans isomerase, and others functioning in the process of photosynthesis. ACC synthase gene encodes the enzyme to regulate the rate-limiting step in ethylene biosynthesis [23], and its expression regulates leaf performance and drought tolerance by increasing or decreasing the concentration of ethylene. The induced ACC synthase by drought resulted in retarded leaf growth under stress. The induced SDR, IAA 26, and auxin hydrogen symporter proteins could dynamically regulate the phytohormone (ABA, IAA, etc.) level to respond to drought stress. Seven genes involved in photosynthesis were specifically down-regulated in rice leaf. This result demonstrates that inhibition of photosynthesis is the major effect of drought responsiveness in rice leaf. Down-regulation of photosynthesis genes under a water deficit situation has been previously reported in rice and other crops [24,25]. The phytohormones of ABA, ethylene, and auxin are highly accumulated in leaf in a drought environment to maintain the homeostasis of plant biosynthesis.

A total of 76 genes were preferentially induced in the roots at the tillering and panicle elongation stages (Additional file 12), except for 35 genes with unknown function. A set of these genes were functionally involved in transcription regulation, such as those encoding one abscisic acid responsive elements-binding factor 1 (AREB1, Os06g0211200), one heat shock transcription factor 31 (HSP31, Os02g0527300), two heat shock proteins (HSPs), three myb transcription factors, one bZip TF, and three protein kinase including OsPK4 (Os01g0206300). All these were identified to be related to the upstream of gene regulation of drought or other abiotic stress responsiveness in plant. The gene *AREB1 *regulates novel ABRE-dependent ABA signaling that enhances plant drought tolerance in vegetative tissues [26].

A relatively large portion of the root-specific repressed genes is involved in metabolism, cell growth, cell wall modification, and phytohormone response (Additional file 13). Fourteen genes related to cell wall biogenesis and modification, such as the genes encoding three ENOD93 proteins, EXPA5, EXPA3, and one cell wall invertase (Os04g0664900), involved in the process of cell wall extension, were apparently down-regulated only in roots. Another root dependent repressed gene set is functionally related to phytohormone regulation. These genes encode four ethylene responsive transcription factors, namely, ER33, GAST1, Auxin efflux carrier protein (Os01g0802700), and GA2-oxidase (GA2ox, Os05g0560900), and two SDR family proteins (Os11g0499600, Os12g0260500). *GAST1 *gene was reported to be oppositely regulated by GA and ABA, with GA inducing and ABA inhibiting its expression at the transcription level in a shoot of tomato [27]; the enzyme GA 2-oxidase antagonizes GA activity by deactivating GAs that regulate leaf expansion, stem elongation, and flower induction [28-30]. Therefore, GA might be promoted due to the down-regulation of GA2ox, whereas ethylene and Auxin were inhibited in root under drought. This finding reveals that root growth under drought is spatially regulated, whereas the response of root cell expansion to water stress is independently regulated in longitudinal and radial directions [31]. From these results, we can speculate that root elongation is enhanced under drought stress so that the stressed rice root can reach deeper water. However, the root may become thinner due to the inhibition of cell wall extension to save more energy to protect the rice plant from drought stress.

Specific drought DEGs were mostly detected in panicle among the three tissues. A total of 102 and 346 genes were identified to be up- and down-regulated specifically in panicle under drought, respectively (Additional files 14 and 15). First, several pollen or anther development-related genes were identified to be particularly regulated by drought in rice panicle. Five genes encoding pollen allergen family proteins, profiling A, and tapetum-specific endoxylanase were up-regulated, whereas another set of genes, such as gelsolin, anther-specific protein YY2, meiotic serine proteinase (Os04g0543700), two allergen V5/Tpx-1 proteins, pistil-specific extension-like protein, plantacyanin, dioxygenase RAMOSUS1 (Os01g0566500), and CUT1 (Os01g0529800) were down-regulated by drought in the panicle only. Among these genes, gene encoding meiotic serine proteinase was significantly down-regulated more than 20 times by drought stress. This gene was found to be related to microsporogenesis. The gene *RAMOSUS1 *encodes dioxygenase and is involved in shoot branching [32], whereas the gene *CUT1 *encoding a very long chain of fatty acid condensing enzyme was identified to be related to cuticular wax biosynthesis and pollen development [33].

A total of 53 genes (53/346) related to membrane biogenesis and cell wall extension were confirmed to be exclusively down-regulated in young panicle (Additional file 15), although they belong to a different gene set from those down-regulated in root mentioned above. These genes encode cellulose synthase, lipid transfer proteins (LTPs), cell wall invertase, laccase, pectinesterase family proteins, and glycoside hydrolase family proteins. The cell wall invertases were involved in the panicle elongation, and their expression could be highly down-regulated by drought stress, resulting in the retardation of panicle elongation in rice [34]. LTPs, pectinesterases, and glycoside hydrolases are functionally related in membrane biogenesis and cell wall extension [35-37]. These results show that the panicle development of rice is greatly repressed when the rice plant is under the condition of water deficit.

Five histone genes were detected to be highly down-regulated by drought exclusively in young panicle: *Histone H2A *(Os03g0162200), *Histone H2A *(Os03g0279200), *Histone H3 *(Os06g0160100), *Histone H3 *(Os05g0438700), and *Histone H3 *(Os01g0866200). These genes are involved in cell division and are reported to be regulated by drought and development [38,39]. However, the effect of the repression of histone genes on cell cycle in rice panicle under drought needs to be further elucidated.

Several genes showing reciprocal expression patterns in two tissues at the same development stage under drought were also identified. After comparing the whole gene profiling between two tissues at the same development stage, several genes were detected to be reciprocally regulated in two tissues under drought. At the tillering stage, 5 and 6 genes were induced and repressed in leaves but repressed and induced in roots, respectively. At the panicle elongation and booting stages, 29 and 4 genes were identified to be reciprocally regulated by drought in two different tissues, respectively (Table 3).

**Table 3:** List of genes with reciprocal action between two tissues at different development stages

Gene ID	Annotation	BP	BL	PL	PR	TL	TR
*At Booting Stage*							
Os08g0547300	E-class P450, group I family protein	3.27	-3.94	-4.25			
Os07g0591700	Conserved hypothetical protein	2.50	-3.35				
Os07g0175600	Plant lipid transfer protein	-2.71	2.51	3.34			
Os03g0793800	Plant lipid transfer protein	-3.32	2.56				
*At Panicle Elongation Stage*							
Os05g0560900	Gibberellin 2-oxidase	2.75	5.02	3.07	-5.20	3.30	-4.82
Os11g0702100	Class III chitinase homologue			6.03	-3.05	2.52	-3.22
Os10g0158100	Senescence-associated protein 15			2.79	-3.37	2.34	-4.26
Os04g0652700	Nuclease I		3.61	3.98	-2.60		-2.99
Os04g0635100	Wound induced protein		4.06	3.29	-3.08		
Os03g0745200	Transferase family protein			3.03	-2.49		-2.64
Os01g0788400	Pectinesterase(Pectin methylesterase)			3.33	-2.71		-3.26
Os03g0830500	PGPS/D12	4.44		4.43	-2.54		-5.23
Os12g0592900	Hypothetical protein		2.98	5.15	-3.43		-2.91
Os10g0418100	Calcium-transporting ATPase 8			2.58	-2.66		-2.86
Os04g0460300	Amino acid/polyamine transporter II		3.01	3.62	-2.64		
Os06g0592400	Cytosolic aldehyde dehydrogenase RF2C			3.06	-3.05		-2.83
Os04g0268700	Eggshell protein family protein			2.62	-4.15		-4.36
Os10g0464000	Hypersensitive-induced response protein			2.37	-2.46		-3.28
Os02g0813100	Cyclin-like F-box domain containing protein			2.65	-3.19		-3.06
Os01g0104200	NAC-domain protein 5-8			3.23	-2.46		-2.52
Os10g0391400	ZIM domain containing protein			2.76	-3.89		-3.86
Os03g0100200	Conserved hypothetical protein		-2.33	-4.35	3.02		
Os09g0469300	Plastocyanin-like domain containing protein			-2.56	5.44		4.68
Os06g0136600	Enolase 1			-2.34	3.13		
Os03g0178500	Alpha/beta hydrolase family protein			-3.22	2.74	-2.31	
Os04g0538000	TPR repeat containing protein			-2.81	3.82		
Os01g0279400	Major facilitator superfamily antiporter		-2.77	-3.75	2.54	-3.35	
Os01g0866400	Fructose-1,6-bisphosphatase			-3.79	3.16		3.22
Os01g0102300	Conserved hypothetical protein			-3.64	4.00		3.32
Os03g0197100	Sugar transporter protein			-4.17	3.35		4.56
Os12g0575000	Protein of unknown function DUF1118			-2.59	3.84		3.93
Os01g0556700	Dicarboxylate transporter			-2.65	5.13		5.61
Os05g0568900	Protease Do-like 1			-2.39	3.10		
*At Tillering Stage*							
Os05g0560900	Gibberellin 2-oxidase	2.75	5.02	3.07	-5.20	3.30	-4.82
Os11g0702100	Class III chitinase homologue			6.03	-3.05	2.52	-3.22
Os10g0158100	Senescence-associated protein 15			2.79	-3.37	2.34	-4.26
Os07g0127600	Allergen V5/Tpx-1 related family protein				-3.04	2.50	-4.30
Os03g0322900	Late embryogenesis abundant protein	3.12	3.33	2.60	5.15	-4.35	3.44
Os04g0685700	Conserved hypothetical					-2.35	2.56
Os01g0946500	Glucan endo-1,3-beta-glucosidase GV	2.50			2.73	-2.42	2.56
Os03g0625300	Quinonprotein alcohol dehydrogenase				3.85	-2.46	3.70
Os01g0789400	Alpha subunit of RNA polymerase					-2.34	3.25
Os09g0402100	PF1 protein					-4.08	2.94

*Note*: Log2 transformed ratios of drought stress and control

Three genes, i.e., *GA2ox*, *Chitinase III*, and *Senescence-associated protein 15 *(*SAP15*), were highly induced in leaf but repressed in root at both the tillering and panicle elongation stages. *GA2ox *was highly down-regulated in all roots but up-regulated in all leaves and panicle. One gene encoding the LEA protein (Os03g0322900) was only down-regulated in tillering leaf but up-regulated in all other tissues under drought. These results provide further evidence that the genes are regulated under environment stress in a tissue-specific manner. *GA2ox *has an important role in the regulation of stem elongation and leaf growth, and participates in the phase transition from vegetative to reproductive growth [40]. Induction of *GA2ox *may result in a low level of GA in leaf under drought by feedback control and retardation of leaf growth, whereas the *GA2ox *is highly repressed by drought. Senescence-associated proteins are involved in leaf senescence in plant [39]. The reciprocal regulation of *SAP15 *in root and leaf indicates that leaf growth was inhibited, whereas root growth was promoted by drought stress. The gene of *Chitinase III *confers disease resistance by degrading chitin, a component of fugal cell wall [41]. The biological role of this gene differently regulated by drought in root and leaf remains unclear.

Organ-/tissue-specific gene regulation in response to abiotic stresses has been previously reported. The gene encoding glutamine synthetase in potato was found to have a differential response to drought and salt stresses in an organ-dependent manner [42,43]. A systematic comparison of gene expression in various rice organs revealed mostly organ-specific reprogramming of genome expression responding to drought and high salinity [15]. These results support that each plant organ has a unique strategy in dealing with environmental stress.

### Genome-wide expression profiling of transcription factor (TF) genes under drought stress

Among the 2384 known or annotated TF genes in the rice genome [44], 261 (10.9%) TF genes were differentially regulated by drought (Additional file 16), accounting for about 5% of total DEGs detected in this study. These TF genes belong to a diverse range of TF families classified by Gao et al. [44] including 35 MYB genes, 28 AP2/EREBP genes, 21 bHLH genes, 11 HSF genes, 27 NAC genes, 15 WRKY genes, etc. (Table 4). Among these TF genes, 153 were found to be differentially regulated by drought at the leaf of panicle elongation stage.

**Table 4:** Drought-induced expression patterns of tissue-specific regulated AP2/EREBP transcription factors

Gene ID	TF Family	TL	PL	BL	TR	PR	BP
Os08g0474000	AP2/EREBP family	4.16	4.05	3.81	3.44		4.24
Os02g0764700	AP2/EREBP family	2.87	6.32	4.24			2.42
Os06g0166400	AP2/EREBP family		3.12	3.34			3.14
Os02g0655200	AP2/EREBP family		3.74	2.48			
Os06g0127100	AP2/EREBP family		4.96	2.75			
Os01g0797600	AP2/EREBP family			2.34			
Os08g0537900	AP2/EREBP family			3.06			
Os04g0610400	AP2/EREBP family			2.97			
Os01g0165000	AP2/EREBP family		4.68				
Os09g0286600	AP2/EREBP family		2.57				
Os03g0183000	AP2/EREBP family		3.68				
Os04g0398000	AP2/EREBP family		2.42				
Os04g0546800	AP2/EREBP family						3.59
Os05g0361700	AP2/EREBP family				3.84	4.64	2.95
Os03g0182800	AP2/EREBP family				4.06	3.84	
Os10g0390800	AP2/EREBP family				2.64	2.74	
Os02g13710	AP2/EREBP family				2.74	2.47	
Os07g0674800	AP2/EREBP family				5.06	6.64	
Os04g0529100	AP2/EREBP family				2.47	2.40	
Os01g0313300	AP2/EREBP family				5.06	3.06	
Os03g0341000	AP2/EREBP family				6.64	5.06	
Os03g0191900	AP2/EREBP family					2.84	
Os09g0522000	AP2/EREBP family					2.74	
Os12g0582900	AP2/EREBP family				2.74		
Os07g0617000	AP2/EREBP family				2.56		
Os09g0287000	AP2/EREBP family				2.32		
Os04g0550200	AP2/EREBP family	2.64		2.74			

*Note*: Log2 transformed ratios of drought stress and control

After comparing the expression patterns of all TF genes, different sets of TFs genes with unique expression patterns were identified. Two TF genes encoding bZIP (*OsbZIP14*, Os01g0867300) and HB (Os02g0649300) proteins were commonly induced in all tissues at the three development stages, indicating that these two TF genes might be involved in the universal regulation of rice response to drought. *OsbZIP14 *was also found to be induced by drought in all tissues during panicle and seed development [45]. A total of 17 and 34 TF genes shared the same expression pattern between two roots and among three leaves under drought, respectively. A total of 15, 13, and 21 TF genes were commonly regulated between two tissues at the tillering stage, panicle elongation stage, and booting stage, respectively. These results imply that only several TF genes of rice were coincidently regulated by drought stress.

Several of the TF genes were tissue- or stage-specifically regulated, especially the plant-specific TF gene family member. The 10 identified HSF genes were highly up-regulated in at least one tissue, 9 of which were induced in the root at the panicle elongation stage. Five members of the GRAS family genes were down-regulated in root or leaf. The three auxin responsive or ARF genes were exclusively repressed in leaf at the panicle elongation stage. Most of the identified bHLH genes (18 out of 21) were found to be down-regulated in either at least one root tissue or one leaf tissue (Additional file 17). These differentially expressed bHLH TF genes were further functionally identified in an experiment of PEG-simulated drought stress and exogenous ABA treatment using quantitative RT-PCR analysis [46]. Result also show that most of the bHLH genes were repressed in seedling roots and leaves under PEG stress and ABA stress [46], implying that these bHLH genes might have negative roles in rice responding to osmotic stress.

As for stage-specific regulated TF genes, three of them, including one ZIP member and two MYB, were down-regulated only at the tillering stage. Nine TF genes were only differentially regulated (the same or different pattern) by drought at booting stage. A total of 25 TF genes belonging to bHLH, GRAP-G2-like, MYB, NAC, and ZIM families were only differentially regulated (the same or different pattern in two tissues) by drought at the panicle elongation stage. There were 7 and 7 MYB and NAC genes induced by drought exclusively in leaf at panicle elongation stage, respectively (Additional file 16). A number of NAC TF genes and several MYB TF genes have been previously found to be differentially expressed in a tissue-specific manner under various abiotic stresses, such high salinity, cold stress, and drought environment [47-49]. This stage-specific gene profiling of TF genes implies that drought stress responsiveness is under developmental control.

AP2/EREBP family proteins are unique to plants and share a highly conserved AP2 domain. Several AP2/EREBP TF genes were involved in transcriptional regulation pathway of ABA-dependent and ABA-independent response to drought stress [50,51]. Two distinct sets of AP2/EREBP transcription factor members were determined in this study. One set of the TFs, including 14 members, was specifically induced by drought in at least one leaf tissue or panicle, except for one, AP2/EREBP TF, which was repressed in TL and BL. Another set of 13 TF genes were repressed only in root, mostly in root samples from both stages, except for one gene that was reciprocally regulated in roots and panicle (Table 4). These results show that AP2/EREBP TF genes are highly under organ-specific regulation by drought. These results were also confirmed by the macroarray analysis of AP2/EREBP TF genes family and RT-PCR analysis of drought differentially regulated TF genes [52].

AP2/EREBP TF genes play an important role in regulating developmental processes. Several of these genes have been identified to be functionally involved in developmental control, such as *APETALA *in identity with the Arabidopsis flower [53], and *AINTEGUMENTA *and *AINTEGUMENTALIKE6 *in flower patterning [54], *PUCHI *affecting root morphogenesis [55]. These organ-dependent regulations of AP2/EREBP TF genes might be responsible for regulating organ-specific downstream genes in response to drought stress.

The rice genome has more than 2000 TF genes [44], majority of which are members of large families. In this study, 261 TF genes were found to be differentially regulated by drought, and most TF genes were tissue- or stage-specific regulated. This finding implies that these TF genes can play different roles in the regulation of rice plant response to drought stress and that the regulation may be under the control of development.

### Regulatory element analysis of commonly induced genes under drought stress

To identify common sequence motifs of drought-induced genes in rice, we examined the *cis*-regulatory elements in the 1 kb regions upstream of 55 commonly induced genes in all tissues using two approaches. First, all known *cis*-elements responsive to drought were used as targets to scan the upstream sequences. The other method used was not based on known elements, in that all possible 6-meric, 8-meric, 10-meric, and 12-meric sequences were evaluated for whether they are overrepresented in the scanned regions using the Weeder software.

Four ABA responsive elements (ABRE) containing an ACGT core sequence (i.e., RTACGTGGCR, ACGTSSSC, TACGTGTC, and ACGTGKC) were used to search the upstream of the 55 genes with the control upstream sequences of 360 genes showing no significant change in expression pattern under drought. We found four elements over-represented in the commonly induced 55 genes compared with the control. A total of 72.7% of the genes contain 1-8 copies of the 4 ABREs, whereas only 3.6% of the control genes harbor 1-3 copies of the elements. Another ABRE motif, S000278 with ACGT core sequence, was also significantly detected to be over-presented in the 55 genes. Around 31.5% of the genes have at least one copy of S000278, whereas only 0.83% of the control genes have the sequence(s).

All 1 kb sequences in the upstream of the 55 genes were submitted to a local installation of Weeder (version 1.3) and an extra mode search (6 bp long with 1 mismatch; 8 bp long with 3 mismatches; 10 bp long with 4 mismatches, and 12 bp long with 4 mismatches) was performed to look for the candidate motifs in a single strand. Four GC-rich sequences, namely, 6 bp of CCGCGC, 8 bp of CGCCGCGC, 10 bp of GCCGCGCGGC, and 12 bp of GCCGCGCCGCGC, were identified. The motif sequence logo is available in the supplementary material (Additional file 18). Most of these four motifs were located between -10 and -500 bp apart from the start codon, but majority of them were in the upstream region from -100 to -300 bp. In total, 48 and 41 out of the 55 genes have at least one or two copies of the four elements (Table 5). CGCG box was identified among these *cis*-elements after further analysis; this special CGCG box is regulated by calmodulin and involved in the transcription regulation of multiple abiotic stresses responsiveness [56]. A number of GC rich motifs with a core motif of CGCG have been identified to be significantly over-represented in the promoter of the *physcomitrella *ABA- and stress-induced gene set [57]. This special CGCG box combined with the ABRE element may be very important for the response of the rice plant to drought environment.

**Table 5:** Copy number of the four cis-elements identified in the upstream regions of 55 commonly induced genes

No	Gene ID	Annotation	S1	S2	S3	S4
1	Os08g0104400	expressed protein	1			
2	Os01g0705200	late embryogenesis abundant protein 3	8	3	4	
3	Os12g0478200	ABA-responsive protein	2			
4	Os03g0305600	expressed protein	3	1		
5	Os01g0225600	late embryogenesis abundant protein Lea14-A	2	1		
6	Os09g0109600	expressed protein	1			
7	Os05g0542500	late embryogenesis abundant protein 3	4	1		
8	Os11g0454300	water stress-inducible protein Rab21	8	3		1
9	Os02g0140800	expressed protein	1		1	
10	Os01g0214500	conserved hypothetical protein	5	2	2	
11	Os04g0266900	transketolase, chloroplast precursor	5	1	2	
12	Os01g0124400	Bowman-Birk type bran trypsin inhibitor precursor	1		1	
13	Os11g0582300	protein SEY1	7	1	1	1
14	Os07g0563400	fiber expressed protein, putative, expressed	7	5	2	
15	Os10g0548100	expressed protein	17	9	5	5
16	Os01g0226400	ATP binding protein, putative, expressed	8	7	3	2
17	Os06g0698300	protein phosphatase 2C	12	3		1
18	Os01g0867300	G-box-binding factor 4	4	2	3	1
19	Os01g0654400	seed maturation protein PM41	4	1	2	
20	Os10g0505900	expressed protein	5		1	
21	Os01g0743500	NADP-dependent malic enzyme	11	4	3	1
22	Os02g0649300	homeobox-leucine zipper protein ATHB-6	7	1	1	
23	Os01g0950900	HYP1	16	8	8	3
24	Os06g0324400	protein LEA25	1			
25	Os03g0133100	expressed protein	13	5	1	3
26	Os05g0572700	protein phosphatase 2C ABI1	21	9	3	2
27	Os11g0454200	dehydrin Rab16B	2	1	1	
28	Os12g0147200	expressed protein	7	3	2	2
29	Os03g0623100	expressed protein	4	3		
30	Os05g0373900	eukaryotic peptide chain release factor subunit 1-1	5	1		
31	Os11g0454000	dehydrin Rab16C	1			
32	Os08g0327700	seed maturation protein	5	2	1	
33	Os04g0610600	embryonic protein DC-8	10	4	3	2
34	Os06g0246500	pyruvate dehydrogenase E1 component alpha subunit	17	5	3	3
35	Os01g0844300	FK506-binding protein 4	4	2	1	
36	Os03g0723400	expressed protein	1			
37	Os06g0341300	late embryogenesis abundant protein D-34	7	1		
38	Os06g0681200	early nodulin 20 precursor	2	1		
39	Os08g0442900	fibroin heavy chain precursor	5	2	1	
40	Os05g0550600	nonspecific lipid-transfer protein AKCS9 precursor	14	4	5	1
41	Os03g0168100	embryonic protein DC-8	7	3	1	
42	Os03g0168000	expressed protein	9	5	2	1
43	Os04g0589800	seed maturation protein	6	5	2	3
44	Os01g0794400	protein disulfide isomerase, putative, expressed	2	1		1
45	Os06g0651200	expressed protein	6			
46	Os11g0453900	dehydrin Rab16D	9	3	1	1
47	Os01g0303300	stress-inducible membrane pore protein	13	1	1	
48	Os05g0468800	expressed protein	12	7	3	2
49	Os11g32890	expressed protein				
50	Os03g0277300	heat shock cognate 70 kDa protein				
51	Os05g0122700	expressed protein				
52	Os11g0181200	expressed protein				
53	Os11g43790	expressed protein				
54	Os03g0286900	expressed protein				
55	Os01g0743600	ATP-dependent peptidase				

*Note*: Log2 transformed ratios of drought stress and control

## Conclusion

A systematic study of gene profiling of rice genome in response to drought stress was carried out using the Affymetrix rice genome array containing 49,824 known or predicted genes. In this study, three main rice tissues including leaf, root, and young panicle from three development stages were sampled for array analysis. This is the first trial to explore comprehensively the genome-wide gene spatial and temporal expression patterns of drought responsiveness in rice. Results show that most drought-responsive genes were under tissue- and stage-specific regulations.

We detected 5,284 transcripts/genes differentially expressed under drought stress, accounting for around 10% of total transcripts on the rice array chips. Only a small amount of genes was identified to be commonly up-regulated by drought in all tissues at three development stages. Most of the DEGs were tissue-specific regulated by drought. Notably, the tissue-specific down-regulated genes showed distinct function categories. Several genes related to photosynthesis were identified to be specifically repressed by drought in leaf. However, a large amount of genes involved in cell membrane biogenesis and cell wall modification was determined to be highly down-regulated, specifically in root and young panicle. We may conclude that photosynthesis in the leaf, panicle elongation, and root growth is significantly inhibited by drought when rice plant is in a water deficit situation.

There was strong interaction between plant development and environment conditions. Plant stress responses often mimic certain normal developmental processes. There was also evidence that systematic regulation of gene expression drives developmental processes and stress response [58,59]. Some stage- or tissue-specific regulated genes are likely to be co-regulated by environment stresses and development cues, and the rice plant response to drought appears to be under developmental regulation.

Transcription regulation plays a central role in stress signal transduction pathways. In this study, we found 261 transcription factor genes differentially regulated at different levels in root, leaf, and young panicle at different development stages. Among these, 153 TF genes were found to be up- or down-regulated in the leaf at the panicle elongation stage. This finding reveals that a large amount of TF genes is involved in the transcription regulation in response to drought stress when rice plant is at the panicle elongation stage.

Numerous *cis-*elements have been previously reported to be important in plant response to drought stress, including ABRE and DRE-like elements, which are the binding sites for bZIP and AP2/EREBP-type transcription factors. Except for the prevalence of ABRE element in the upstream of commonly induced 55 genes in this study, a CGCG box motif was also found to be a probable candidate in *cis*-element for the transcription regulation of drought responsiveness in rice. However, whether this element functions as a transcription factor binding site needs to be further determined.

## Authors' contributions

BF and ZL designed the experiments and drafted the manuscript. DW, YP, XZ, and LZ performed the phenotypic experiment and the microarray experiment. BF designed the microarray experiments, performed the data analyses of microarray, and revised the manuscript. All authors have read and approved the final manuscript.

## Supplementary Material

Additional file 1 **Pedigree of the variety DK151**. A word file containing the BC breeding and intercross procedures for developing drought tolerant introgression lines and pyramiding line, DK151 using IR64 (the recipient) and two donors, BR24 and Binam. Click here for file

Additional file 2 **Total number of genes expressed in different samples under control and drought stressed conditions**. Excel file containing the summary result of expressed gene number in different samples. Click here for file

Additional file 3 **Hierarchical cluster analysis of six tissue types and all DEGs under drought stress**. PPT file containing the result of the hierarchical cluster analysis. Click here for file

Additional file 4 **List of primers for the RT-PCR**. Excel file containing all primer sequences used for the RT-PCR experiment. Click here for file

Additional file 5 **Semi-quantitative RT-PCR confirmation of microarray data**. Description: A ppt file containing semi-quantitative RT-PCR confirmation of microarray data. The microarray data are shown on the left side, and the RT-PCR results are shown on the right side. A total of 21 genes were differentially regulated by drought at the tillering stage, panicle elongation stage, and booting stage. TLC and TLS indicate leaves under control and under stress, and TRC and TRS indicate root under control and stress at the tillering stage, respectively. PLC and PLS indicate leaves under control and stress, and PRC and PRS indicate root under control and stress at the panicle elongation stage, respectively. BLC and BLS indicate leaves under control and stress, and BPC and BPS indicate panicle under control and stress at the booting stage, respectively. Click here for file

Additional file 6 **Comparative diagram of the tissue-specific DEGs under drought stress**. A ppt file containing a comparative diagram of the total number of up-, down-regulated, and common regulated genes between leaves and root at the tillering stage (TL, TR), leaves and root at the panicle elongation stage (PL, PR), and leaves and panicle at the booting stage (BL, BP) under drought stress. Click here for file

Additional file 7 **Venn diagram of all tissue up- and down-regulated DEGs under drought stress**. PPT file for the result of Venn Diagram of all tissue-specific DEGs. Click here for file

Additional file 8 **List of commonly induced genes by drought in all tissues at all development stages**. Excel file containing the list of the commonly induced genes in all samples Click here for file

Additional file 9 **List of commonly down-regulated genes by drought in all tissues at all development stages**. Excel file containing the list of the commonly down-regulated genes in all samples Click here for file

Additional file 10 **Leaf specific up-regulated genes under drought stress**. Excel file containing all specific up-regulated genes by drought in leaf. Click here for file

Additional file 11 **Leaf specific down-regulated genes under drought stress**. Excel file containing all specific down-regulated genes by drought in leaf. Click here for file

Additional file 12 **Root-specific up-regulated genes under drought stress**. Excel file containing all specific up-regulated genes by drought in roots. Click here for file

Additional file 13 **Root-specific down-regulated genes under drought stress**. Excel file containing all specific down-regulated genes by drought in roots Click here for file

Additional file 14 **Panicle-specific up-regulated genes under drought stress**. Excel file containing all specific up-regulated genes by drought in panicle Click here for file

Additional file 15 **Panicle-specific down-regulated genes under drought stress**. Excel file containing all specific down-regulated genes by drought in panicle Click here for file

Additional file 16 **List of TF genes differentially regulated under drought stress**. Excel file containing all transcription factor genes differentially regulated by drought stress in all samples Click here for file

Additional file 17 **List of TF gene stages specifically regulated by drought stress**. Excel file of the identified transcription factor genes specifically regulated by drought stress Click here for file

Additional file 18 **Sequence Logo of the core sequences of the identified four motifs**. A PPT file of the sequence logo of the core sequences of the identified motifs Click here for file
