# Supplementary material for: Genome-wide temporal-spatial gene expression profiling of drought responsiveness in rice
Source: BMC Genomics. 2011 Mar 16;12:149. doi: 10.1186/1471-2164-12-149 (PMC3070656; doi:10.1186/1471-2164-12-149)
Supplement: Additional file 1 — Pedigree of the variety DK151. A word file containing the BC breeding and intercross procedures for developing drought tolerant introgression lines and pyramiding line, DK151 using IR64 (the recipient) and two donors, BR24 and Binam. [file 1471-2164-12-149-S1.PPT]

## Slide 1
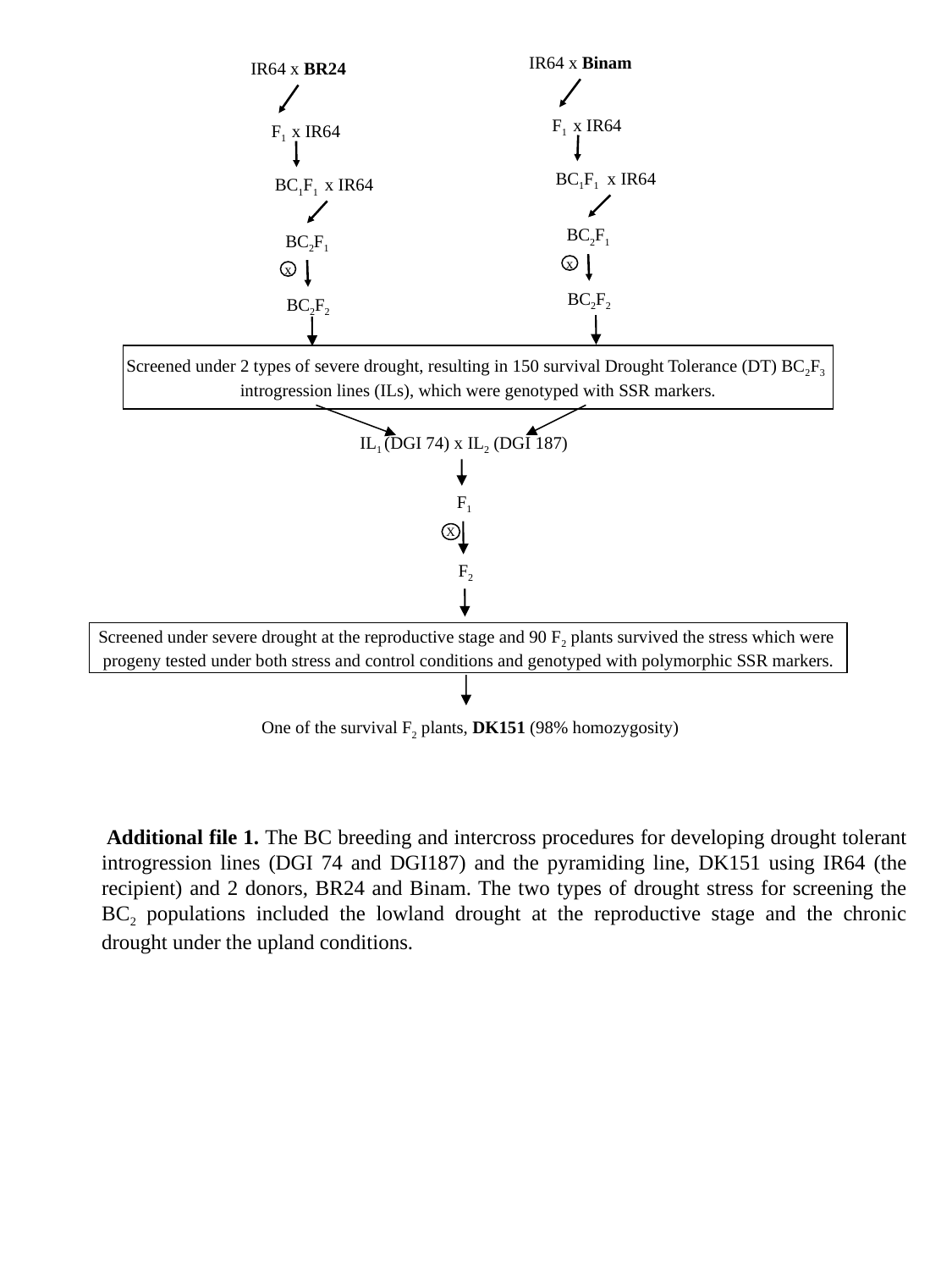

IR64 x Binam
F1
x IR64
BC1F1
x IR64
BC2F1
x
BC2F2
IR64 x BR24
F1
x IR64
BC1F1
x IR64
BC2F1
x
BC2F2
Screened under 2 types of severe drought, resulting in 150 survival Drought Tolerance (DT) BC2F3
introgression lines (ILs), which were genotyped with SSR markers.
IL1 (DGI 74) x IL2 (DGI 187)
F1
X
F2
Screened under severe drought at the reproductive stage and 90 F2 plants survived the stress which were
progeny tested under both stress and control conditions and genotyped with polymorphic SSR markers.
One of the survival F2 plants, DK151 (98% homozygosity)
 Additional file 1. The BC breeding and intercross procedures for developing drought tolerant introgression lines (DGI 74 and DGI187) and the pyramiding line, DK151 using IR64 (the recipient) and 2 donors, BR24 and Binam. The two types of drought stress for screening the BC2 populations included the lowland drought at the reproductive stage and the chronic drought under the upland conditions.
