# Supplementary material for: Genome-wide temporal-spatial gene expression profiling of drought responsiveness in rice
Source: BMC Genomics. 2011 Mar 16;12:149. doi: 10.1186/1471-2164-12-149 (PMC3070656; doi:10.1186/1471-2164-12-149)
Supplement: Additional file 2 — Total number of genes expressed in different samples under control and drought stressed conditions. Excel file containing the summary result of expressed gene number in different samples. [file 1471-2164-12-149-S2.DOC]

| Sample | Control | Percentage% | Drought Stressed | Percentage% |
| --- | --- | --- | --- | --- |
| Leave at tillering stage | 19511 | 39.2 | 20178 | 40.5 |
| Root at tillering stage | 21569 | 43.3 | 21596 | 43.3 |
| Leave at panicle elongation stage | 19272 | 38.7 | 19524 | 39.2 |
| Root at panicle elongation stage | 21663 | 43.5 | 20489 | 41.1 |
| Leave at booting stage | 18976 | 38.1 | 20911 | 41.9 |
| Panicle at booting stage | 23158 | 46.5 | 23068 | 46.3 |

**Additional file 2**. Total number of genes expressed in different sample under control and drought stressed condition
