# Supplementary material for: Genome-wide temporal-spatial gene expression profiling of drought responsiveness in rice
Source: BMC Genomics. 2011 Mar 16;12:149. doi: 10.1186/1471-2164-12-149 (PMC3070656; doi:10.1186/1471-2164-12-149)
Supplement: Additional file 3 — Hierarchical cluster analysis of six tissue types and all DEGs under drought stress. PPT file containing the result of the hierarchical cluster analysis. [file 1471-2164-12-149-S3.PPT]

## Slide 1
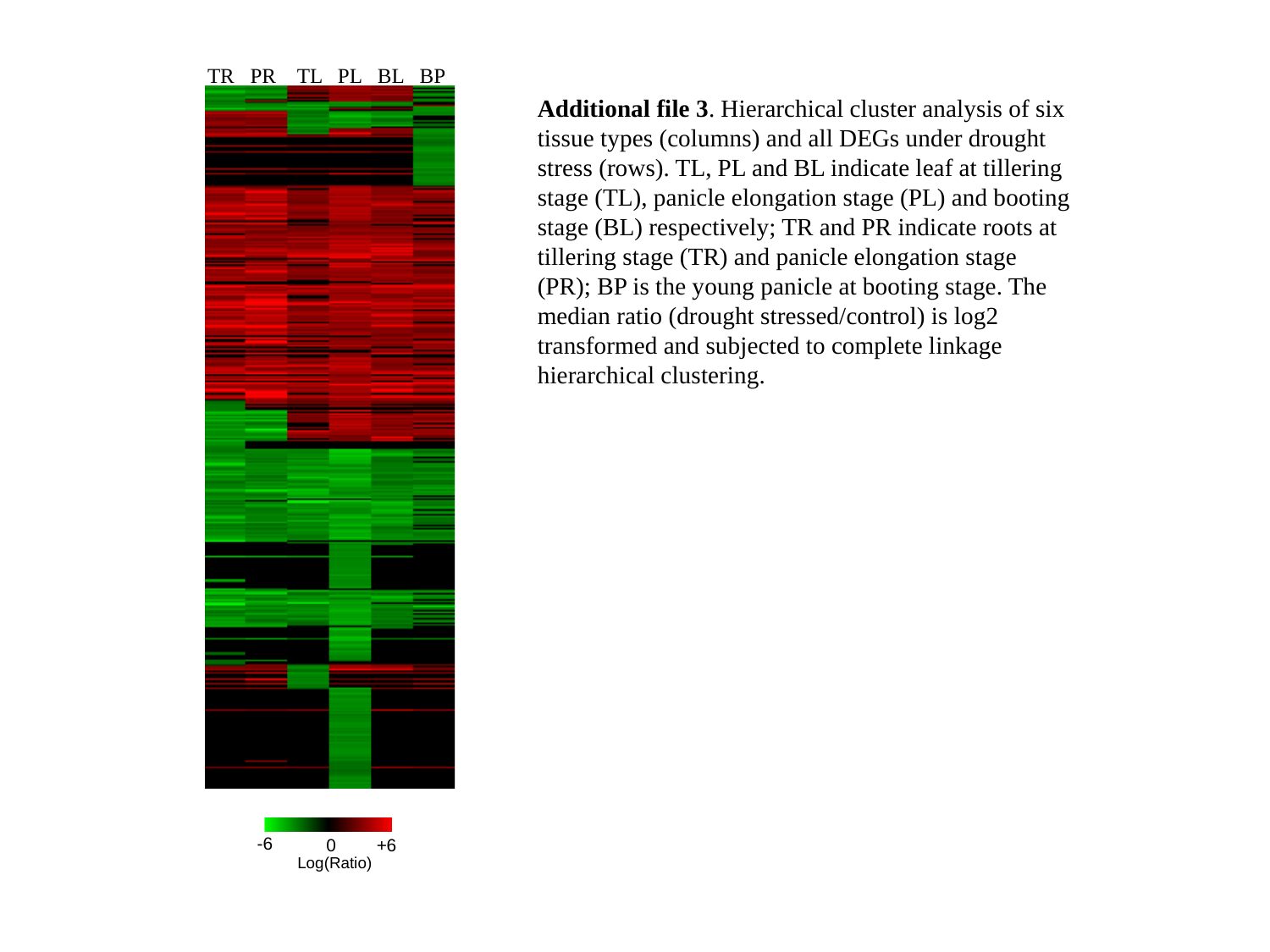

TR PR TL PL BL BP
 -6
0
+6
Log(Ratio)
Additional file 3. Hierarchical cluster analysis of six tissue types (columns) and all DEGs under drought stress (rows). TL, PL and BL indicate leaf at tillering stage (TL), panicle elongation stage (PL) and booting stage (BL) respectively; TR and PR indicate roots at tillering stage (TR) and panicle elongation stage (PR); BP is the young panicle at booting stage. The median ratio (drought stressed/control) is log2 transformed and subjected to complete linkage hierarchical clustering.
