# Supplementary material for: Genome-wide temporal-spatial gene expression profiling of drought responsiveness in rice
Source: BMC Genomics. 2011 Mar 16;12:149. doi: 10.1186/1471-2164-12-149 (PMC3070656; doi:10.1186/1471-2164-12-149)
Supplement: Additional file 4 — List of primers for the RT-PCR. Excel file containing all primer sequences used for the RT-PCR experiment. [file 1471-2164-12-149-S4.DOC]

| Primer Name | Forward Primer(5'-3') | Reverse Primer (5'-3') |
| --- | --- | --- |
| Os05g0560900 | TTCTTCAAGGCGACCAAC | CATGTCGAGCTTCTCCTG |
| Os05g03960 | GCTAACTTCTTGTTCGACG | TTGTGCTGGTCGACGTTC |
| Os11g0702100 | GACAAAGCCTACGATAAC | ATTGAAGCTCCGATGAAG |
| Os10g0158100 | ACGGCGGCAGTGATGGAG | AGCTTGACGTACTTGAGG |
| Os07g0127600 | ATCGTCAACATCCACAAC | TAGTTGTTGCGATCGGAG |
| Os03g0322900 | CTACACCGCCGACAAGAC | TTGTACTCGCCCAGCTTG |
| Os04g0685700 | CGCGAGTGGTACCTCATG | CAGTGAGCGGAATCCGAC |
| Os03g0100200 | ACCGTCAATGACTTCATC | ATGTGAATGAAGAATCAGG |
| Os09g0469300 | AGATTGGTCATCTGACATC | GCAACTCACGACGAGTAG |
| Os06g0136600 | GCCATCAATCTACTACTGC | TTACATATTGGTGCTCAAG |
| Os04g0538000 | CTCAGGCTACCGAACAATG | GTGTTGGATCGCCGTCTC |
| Os01g0279400 | CAATTCAGTCGTTGTTTCC | ATGGCACTGCACTGATAG |
| Os01g0102300 | ATACACCAACGGACAAGG | TTGGACGAGTTGTACTTG |
| Os03g0197100 | CTTCGACATCGTGTGTTG | AAGGCCAACAAGCTGATG |
| Os12g0575000 | ACGAGAGCATTGGTGTAC | CCTAATCAACATCAGCTAG |
| Os05g0568900 | AATCAGTTCAGCTGCAAC | CAACTGTATCAACTGGAATG |
| Os04g0652700 | CTCATCAAGTCCATATGATC | TACCAATGGACGATGATG |
| Os01g0788400 | AGTTGCCATGTTCGCATG | ATCTGCGCCATTAGCATG |
| Os03g0830500 | GCTAATTAAGGTACCAGC | ATCGATCTCCTACTTTCC |
| Os12g0592900 | AGAGTAGTAGACGATGACG | AACTGAAGCAAGTAGTAGC |
| Os10g0418100 | TTCGCCTACAAGCAAGTC | TTTACCTCTGGTCTGCAC |
| Os04g0460300 | TACACGACGCTGATCAAC | TTGACGGACCTCTTGTTG |
| Os06g0592400 | TTCTTTCTGGATCTCGTC | TCTGTAATAATCTTCCACC |
| Os02g0813100 | GAGAATGAATCGAGACGC | CCTCGTTGAAGAACTCAC |
| Os10g0391400 | TGATCAGTACACGTCAGC | TGACAGATAGGAATAATCG |
| Os03g0161400 | GTGAAGTTGCAAGCTCTG | TGTCCATCTCGTAGGATC |
| Os01g0733500 | GTTGATCGCTTCAGTAGG | CTACATCAACGGTTGTGC |
| Os09g0458900 | ATCGAGTACGCGCAGAAG | TAGTAGGCGACGATCCTG |

**Additional file 4**. Primers list for the RT-PCR
