# Supplementary material for: Genome-wide temporal-spatial gene expression profiling of drought responsiveness in rice
Source: BMC Genomics. 2011 Mar 16;12:149. doi: 10.1186/1471-2164-12-149 (PMC3070656; doi:10.1186/1471-2164-12-149)
Supplement: Additional file 5 — Semi-quantitative RT-PCR confirmation of microarray data. Description: A ppt file containing semi-quantitative RT-PCR confirmation of microarray data. The microarray data are shown on the left side, and the RT-PCR results are shown on the right side. A total of 21 genes were differentially regulated by drought at the tillering stage, panicle elongation stage, and booting stage. TLC and TLS indicate leaves under control and under stress, and TRC and TRS indicate root under control and stress at the tillering stage, respectively. PLC and PLS indicate leaves under control and stress, and PRC and PRS indicate root under control and stress at the panicle elongation stage, respectively. BLC and BLS indicate leaves under control and stress, and BPC and BPS indicate panicle under control and stress at the booting stage, respectively. [file 1471-2164-12-149-S5.PPT]

## Slide 1
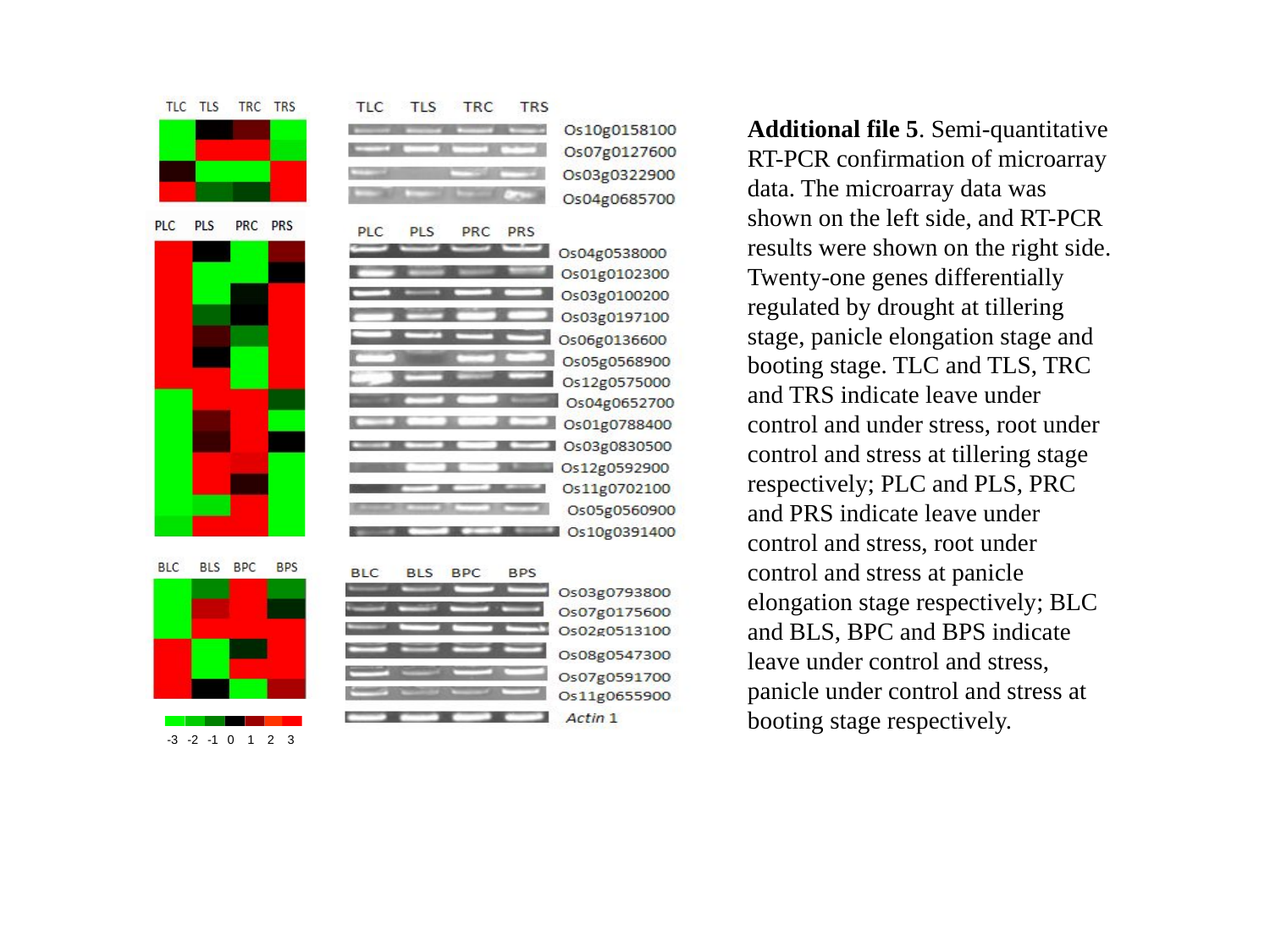

-3
-2
-1
0
1
2
3
Additional file 5. Semi-quantitative RT-PCR confirmation of microarray data. The microarray data was shown on the left side, and RT-PCR results were shown on the right side. Twenty-one genes differentially regulated by drought at tillering stage, panicle elongation stage and booting stage. TLC and TLS, TRC and TRS indicate leave under control and under stress, root under control and stress at tillering stage respectively; PLC and PLS, PRC and PRS indicate leave under control and stress, root under control and stress at panicle elongation stage respectively; BLC and BLS, BPC and BPS indicate leave under control and stress, panicle under control and stress at booting stage respectively.
