# Supplementary material for: Genome-wide temporal-spatial gene expression profiling of drought responsiveness in rice
Source: BMC Genomics. 2011 Mar 16;12:149. doi: 10.1186/1471-2164-12-149 (PMC3070656; doi:10.1186/1471-2164-12-149)
Supplement: Additional file 6 — Comparative diagram of the tissue-specific DEGs under drought stress. A ppt file containing a comparative diagram of the total number of up-, down-regulated, and common regulated genes between leaves and root at the tillering stage (TL, TR), leaves and root at the panicle elongation stage (PL, PR), and leaves and panicle at the booting stage (BL, BP) under drought stress. [file 1471-2164-12-149-S6.PPT]

## Slide 1
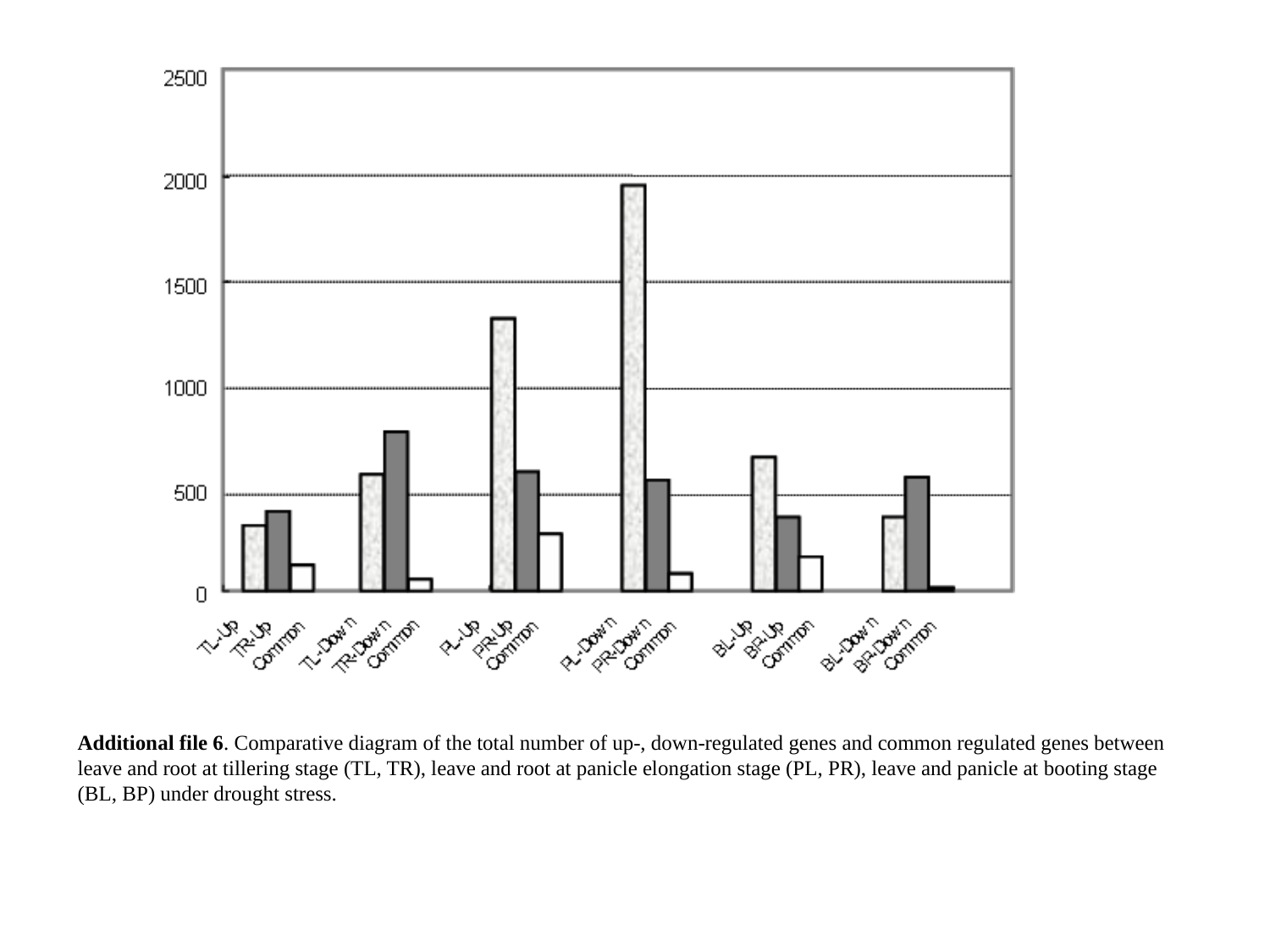

Additional file 6. Comparative diagram of the total number of up-, down-regulated genes and common regulated genes between leave and root at tillering stage (TL, TR), leave and root at panicle elongation stage (PL, PR), leave and panicle at booting stage (BL, BP) under drought stress.
