# Supplementary material for: Genome-wide temporal-spatial gene expression profiling of drought responsiveness in rice
Source: BMC Genomics. 2011 Mar 16;12:149. doi: 10.1186/1471-2164-12-149 (PMC3070656; doi:10.1186/1471-2164-12-149)
Supplement: Additional file 7 — Venn diagram of all tissue up- and down-regulated DEGs under drought stress. PPT file for the result of Venn Diagram of all tissue-specific DEGs. [file 1471-2164-12-149-S7.PPT]

## Slide 1
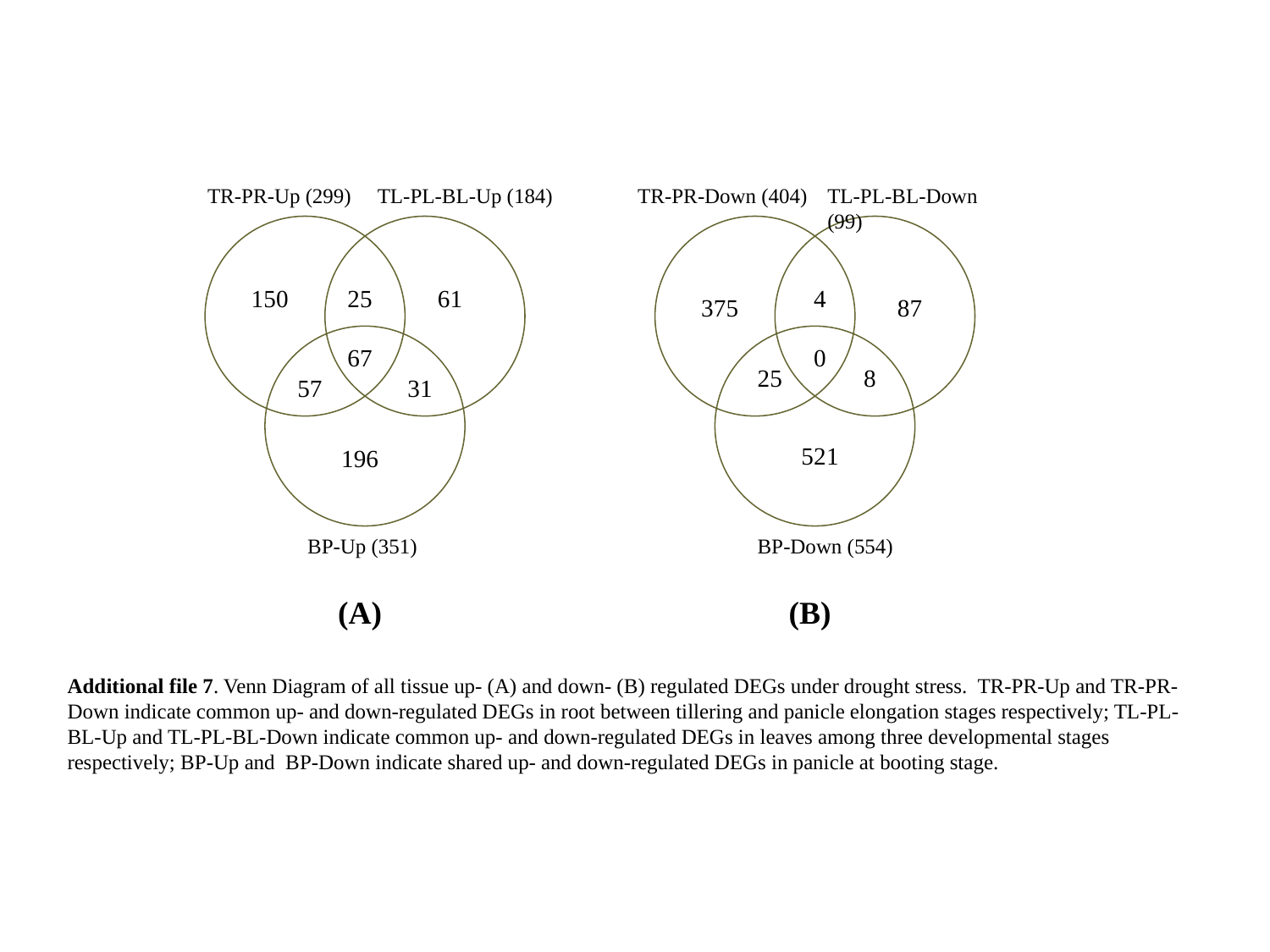

TR-PR-Up (299)
TL-PL-BL-Up (184)
TR-PR-Down (404)
TL-PL-BL-Down (99)
150
25
61
4
375
87
67
0
25
8
57
31
521
196
BP-Up (351)
BP-Down (554)
(A)
(B)
Additional file 7. Venn Diagram of all tissue up- (A) and down- (B) regulated DEGs under drought stress. TR-PR-Up and TR-PR-Down indicate common up- and down-regulated DEGs in root between tillering and panicle elongation stages respectively; TL-PL-BL-Up and TL-PL-BL-Down indicate common up- and down-regulated DEGs in leaves among three developmental stages respectively; BP-Up and BP-Down indicate shared up- and down-regulated DEGs in panicle at booting stage.
