# Supplementary material for: Genome-wide temporal-spatial gene expression profiling of drought responsiveness in rice
Source: BMC Genomics. 2011 Mar 16;12:149. doi: 10.1186/1471-2164-12-149 (PMC3070656; doi:10.1186/1471-2164-12-149)
Supplement: Additional file 8 — List of commonly induced genes by drought in all tissues at all development stages. Excel file containing the list of the commonly induced genes in all samples [file 1471-2164-12-149-S8.DOC]

**Additional file 8.The list of commonly induced genes in all tissues at all development stages**

| Gene ID | Annotation | BP | BL | PL | TL | PR | **TR** |
| --- | --- | --- | --- | --- | --- | --- | --- |
| Os01g0225600 | Late embryogenesis abundant protein Lea14-A | 15.89 | 22.26 | 71.59 | 7.98 | 107.46 | 82.40 |
| Os06g0324400 | Late embryogenesis abundant (LEA) group 1 family protein | 126.80 | 420.45 | 1358.43 | 58.91 | 193.97 | 183.11 |
| Os08g0327700 | Late embryogenesis abundant (LEA) group 1 family protein | 51.95 | 73.57 | 399.69 | 12.48 | 103.65 | 97.33 |
| Os05g0542500 | Late embryogenesis abundant (LEA) group 3 family protein | 5.02 | 76.50 | 237.26 | 189.51 | 12.80 | 65.88 |
| Os04g0589800 | Late embryogenesis abundant (LEA) group 1 family protein | 15.77 | 68.82 | 253.63 | 30.99 | 31.27 | 31.79 |
| Os01g0705200 | Late embryogenesis abundant protein | 25.27 | 116.97 | 178.93 | 33.52 | 59.85 | 27.34 |
| Os03g0168100 | Late embryogenesis abundant protein | 12.09 | 42.79 | 220.60 | 24.65 | 98.94 | 95.46 |
| Os03g0277300 | Heat shock protein 70 | 5.17 | 26.23 | 118.88 | 72.24 | 78.27 | 54.08 |
| Os01g0743600 | Peptidase S16, lon N-terminal domain containing protein | 10.79 | 46.36 | 171.50 | 33.63 | 174.22 | 116.85 |
| Os11g0453900 | Dehydrin RAB 16D | 14.81 | 39.09 | 157.59 | 36.45 | 19.77 | 122.43 |
| Os11g0454000 | Dehydrin family protein | 9.31 | 34.95 | 64.32 | 13.08 | 34.94 | 44.28 |
| Os11g0454200 | Dehydrin RAB 16B | 42.51 | 50.33 | 189.79 | 266.78 | 48.32 | 109.00 |
| Os11g0454300 | Dehydrin family protein | 6.81 | 22.14 | 72.19 | 90.75 | 19.57 | 71.39 |
| Os03g0286900 | Low-temperature induced protein lt101.2 | 8.31 | 62.60 | 120.38 | 28.43 | 18.03 | 5.31 |
| Os04g0610600 | Cor14b protein precursor | 23.98 | 57.21 | 215.80 | 12.56 | 96.73 | 79.61 |
| Os05g0468800 | Cold-regulated protein | 37.99 | 27.07 | 101.64 | 24.14 | 96.14 | 72.43 |
| Os06g0341300 | Seed maturation protein domain containing protein | 82.70 | 64.99 | 84.15 | 36.01 | 100.99 | 111.14 |
| Os01g0743500 | NADP-dependent malic enzyme | 9.26 | 35.38 | 137.16 | 11.28 | 102.79 | 63.52 |
| Os06g0246500 | Pyruvate dehydrogenase E1 alpha subunit | 15.12 | 36.93 | 32.14 | 22.98 | 33.88 | 58.30 |
| Os05g0122700 | Small hydrophobic protein 2 | 38.37 | 70.36 | 128.67 | 20.50 | 81.82 | 47.61 |
| Os11g0582300 | Root hair defective 3 GTP-binding family protein | 5.98 | 9.39 | 7.60 | 5.44 | 18.69 | 28.92 |
| Os07g0563400 | Cotton fibre expressed family protein | 5.59 | 16.60 | 13.70 | 8.26 | 19.91 | 16.27 |
| Os01g0844300 | Peptidylprolyl isomerase | 19.07 | 14.96 | 35.64 | 24.94 | 101.78 | 143.20 |
| Os01g0226400 | AAA ATPase, central region domain containing protein | 21.36 | 53.63 | 79.25 | 39.47 | 87.19 | 92.47 |
| Os01g0124400 | Proteinase inhibitor I12, Bowman-Birk family protein | 7.05 | 12.19 | 15.39 | 6.33 | 7.17 | 5.98 |
| Os01g0794400 | Thioredoxin domain 2 containing protein | 5.15 | 21.99 | 11.16 | 10.68 | 21.98 | 31.61 |
| Os05g0572700 | Protein phosphatase 2C | 5.45 | 43.81 | 112.43 | 16.48 | 24.58 | 10.11 |
| Os06g0698300 | Protein phosphatase 2C family protein | 15.72 | 34.68 | 57.18 | 23.86 | 43.41 | 29.75 |
| Os12g0478200 | GRAM domain containing protein | 13.09 | 18.85 | 72.66 | 7.76 | 18.82 | 19.86 |
| Os05g0373900 | Eukaryotic peptide chain release factor subunit 1 | 7.74 | 23.56 | 76.38 | 25.72 | 52.91 | 49.93 |
| Os01g0867300 | OSE2-like protein | 10.57 | 27.69 | 26.22 | 22.27 | 12.09 | 14.62 |
| Os06g0681200 | Plastocyanin-like domain containing protein | 19.27 | 43.26 | 50.76 | 10.96 | 58.72 | 54.26 |
| Os01g0303300 | Mitochondrial import inner membrane translocase | 12.74 | 21.07 | 76.22 | 10.46 | 55.03 | 34.11 |
| Os03g0305600 | Mitochondrial import inner membrane translocase | 13.57 | 41.72 | 19.45 | 20.57 | 54.88 | 57.22 |
| LOC_Os11g32890 | Expressed protein | 54.97 | 59.14 | 145.00 | 67.66 | 58.81 | 129.40 |
| LOC_Os11g43790 | Expressed protein | 46.92 | 48.11 | 15.77 | 35.60 | 7.19 | 5.51 |
| Os01g0214500 | Conserved hypothetical protein | 11.93 | 25.71 | 22.15 | 10.09 | 5.69 | 6.46 |
| Os01g0654400 | Conserved hypothetical protein. | 84.95 | 11.01 | 20.22 | 7.42 | 126.02 | 106.63 |
| Os01g0950900 | Protein of unknown function DUF221 | 5.02 | 9.19 | 9.72 | 9.61 | 15.89 | 13.35 |
| Os02g0140800 | Conserved hypothetical protein | 26.27 | 62.39 | 101.09 | 50.38 | 48.84 | 84.37 |
| Os02g0649300 | Short highly repeated, interspersed DNA | 24.51 | 67.21 | 106.02 | 9.76 | 9.90 | 16.15 |
| Os03g0133100 | Hypothetical protein | 23.15 | 113.22 | 429.27 | 14.61 | 163.08 | 113.08 |
| Os03g0623100 | Conserved hypothetical protein | 46.80 | 154.69 | 433.72 | 21.77 | 75.33 | 64.63 |
| Os03g0723400 | Conserved hypothetical protein. | 34.51 | 28.77 | 74.24 | 12.35 | 42.26 | 38.07 |
| Os04g0266900 | Bacterial transketolase family protein | 7.55 | 14.01 | 26.41 | 13.55 | 16.28 | 14.50 |
| Os05g0550600 | Nonspecific lipid transfer protein | 17.51 | 23.18 | 102.30 | 7.42 | 60.51 | 43.26 |
| Os06g0651200 | Hypothetical protein | 11.94 | 26.97 | 28.52 | 7.15 | 22.51 | 17.59 |
| Os08g0104400 | Conserved hypothetical protein. | 35.22 | 32.00 | 174.78 | 75.16 | 200.83 | 233.12 |
| Os08g0442900 | Conserved hypothetical protein. | 9.56 | 23.04 | 57.22 | 20.92 | 67.23 | 51.89 |
| Os09g0109600 | Conserved hypothetical protein. | 25.23 | 107.96 | 158.12 | 135.64 | 63.34 | 124.03 |
| Os10g0505900 | Conserved hypothetical protein. | 38.42 | 44.20 | 70.78 | 29.48 | 41.22 | 36.25 |
| Os10g0548100 | Conserved hypothetical protein. | 15.43 | 25.99 | 40.49 | 14.60 | 20.34 | 36.56 |
| Os11g0181200 | Conserved hypothetical protein. | 71.13 | 37.71 | 185.04 | 8.60 | 122.50 | 89.83 |
| Os12g0147200 | Conserved hypothetical protein. | 24.34 | 12.95 | 19.68 | 15.67 | 123.74 | 84.20 |
| Os.39000.1.S1_at | Unknown | 9.14 | 11.12 | 22.31 | 13.25 | 44.24 | 29.10 |
| Os.55227.1.S1_at | Unknown | 12.97 | 46.36 | 133.62 | 24.50 | 57.65 | 70.22 |
