# Supplementary material for: Genome-wide temporal-spatial gene expression profiling of drought responsiveness in rice
Source: BMC Genomics. 2011 Mar 16;12:149. doi: 10.1186/1471-2164-12-149 (PMC3070656; doi:10.1186/1471-2164-12-149)
Supplement: Additional file 9 — List of commonly down-regulated genes by drought in all tissues at all development stages. Excel file containing the list of the commonly down-regulated genes in all samples [file 1471-2164-12-149-S9.DOC]

**Additional file 9.The list of commonly down-regulated genes in all tissues at all development stages**

| **Gene ID** | **Annotation** | **BP** | **BL** | **PL** | **TL** | **PR** | **TR** |
| --- | --- | --- | --- | --- | --- | --- | --- |
| Os.32108.1.S1_s_at | Os01g0312500 Pectin methylesterase isoform alpha (cell wall metabolism) | 0.09 | 0.43 | 0.18 | 0.08 | 0.29 | 0.36 |
| Os.4619.1.S1_at | Unknown | 0.17 | 0.25 | 0.21 | 0.07 | 0.36 | 0.20 |
| Os.54563.1.S1_at | Os03g0605300 Subtilisin-like protease | 0.11 | 0.36 | 0.36 | 0.25 | 0.49 | 0.45 |
| Os.18556.1.S1_x_at | Os03g0857500 Conserved hypothetical protein. | 0.14 | 0.15 | 0.05 | 0.15 | 0.25 | 0.27 |
| Os.12288.1.S1_at | Os08g0237000 End-xyloglucan transferase (cell division and expansion). | 0.20 | 0.46 | 0.32 | 0.06 | 0.46 | 0.39 |
| OsAffx.4214.1.S1_x_at | Os04g0689500 Conserved hypothetical protein. | 0.15 | 0.34 | 0.13 | 0.08 | 0.23 | 0.23 |
| Os.20702.1.S1_at | Os04g0623800 Aminomethyltransferase, mitochondrial precursor (EC 2.1.2.10) | 0.16 | 0.27 | 0.03 | 0.39 | 0.24 | 0.27 |
| Os.27524.2.S1_at | Os09g0338500 Desaturase/cytochrome b5 protein (amphipathic mobile membrane protein). | 0.16 | 0.37 | 0.23 | 0.49 | 0.40 | 0.39 |
| Os.20580.1.S1_at | Os02g0802200 Glycoside hydrolase, family 79, N-terminal domain containing protein (cell wall biosynthesis). | 0.17 | 0.23 | 0.13 | 0.15 | 0.43 | 0.27 |
| Os.8597.1.S1_at | Os03g0111200 Remorin (transmembrane protein). | 0.18 | 0.31 | 0.38 | 0.14 | 0.49 | 0.23 |
| Os.11155.1.S1_at | Os03g0661300 Beta-tubulin (membrane complexes). | 0.15 | 0.24 | 0.23 | 0.16 | 0.33 | 0.18 |
| Os.7940.1.S1_at | Os06g0531900 Lipolytic enzyme, G-D-S-L family protein (Lipid metabolism). | 0.16 | 0.31 | 0.18 | 0.16 | 0.37 | 0.25 |
| Os.17301.1.S1_at | Os10g0534900 Homothorax protein (Fragment). | 0.19 | 0.34 | 0.06 | 0.48 | 0.21 | 0.28 |
| Os.16282.1.A1_at | Os06g0567200 Multicopper oxidase, type 1 family protein. | 0.17 | 0.17 | 0.07 | 0.47 | 0.48 | 0.45 |
| Os.9524.1.S1_at | Os01g0652100 Protein of unknown function DUF231 domain containing protein. | 0.16 | 0.34 | 0.42 | 0.16 | 0.25 | 0.23 |
| Os.8615.1.S1_at | Os08g0492000 Oligopeptide transporter OPT superfamily protein. | 0.13 | 0.19 | 0.05 | 0.19 | 0.26 | 0.21 |
| Os.6786.1.S1_a_at | Os10g0555900 Beta-expansin precursor. | 0.12 | 0.05 | 0.02 | 0.02 | 0.41 | 0.19 |
| Os.50799.1.S1_at | Os04g0569300 Membrane protein. | 0.11 | 0.32 | 0.08 | 0.11 | 0.12 | 0.13 |
| Os.4644.1.S1_at | Os01g0823100 Alpha-expansin OsEXPA2. | 0.08 | 0.10 | 0.03 | 0.03 | 0.23 | 0.37 |
| Os.6092.1.S1_at | Os02g0661100 Trehalose-6-phosphate phosphatase. | 0.13 | 0.16 | 0.21 | 0.18 | 0.07 | 0.06 |
