# Supplementary material for: Genome-wide temporal-spatial gene expression profiling of drought responsiveness in rice
Source: BMC Genomics. 2011 Mar 16;12:149. doi: 10.1186/1471-2164-12-149 (PMC3070656; doi:10.1186/1471-2164-12-149)
Supplement: Additional file 10 — Leaf specific up-regulated genes under drought stress. Excel file containing all specific up-regulated genes by drought in leaf. [file 1471-2164-12-149-S10.DOC]

**Additional file 10**. Leave specific up-regulated genes

| **Gene ID** | **Annotation** | **BP** | **BL** | **PL** | **TL** | **PR** | **TR** |
| --- | --- | --- | --- | --- | --- | --- | --- |
| OsAffx.24138.1.S1_s_at | Os02g0162600 Conserved hypothetical protein. |  | 110.99 | 9.26 | 26.77 |  |  |
| Os.11666.1.S1_at | Os09g0532000 Conserved hypothetical protein. | 2.05 | 66.68 | 31.03 | 10.73 |  |  |
| Os.11327.1.S1_at | Os03g0291200 Protein of unknown function DUF231 domain containing protein. | 2.01 | 51.02 | 62.56 | 5.99 |  |  |
| Os.38812.1.S1_at | Unknown |  | 34.82 | 92.54 | 15.46 | 3.87 | 3.71 |
| Os.26565.3.S1_at | Os01g0200300 Hypothetical protein. |  | 34.72 | 9.69 | 12.88 |  |  |
| Os.51752.2.S1_x_at | Os05g0170200 Conserved hypothetical protein. | 4.91 | 22.79 | 54.20 | 5.31 | 3.73 |  |
| Os.52555.1.S1_at | Os06g0683100 Hypothetical protein. |  | 21.08 | 30.86 | 9.23 | 2.65 | 3.85 |
| Os.12995.1.S1_at | Os04g0690500 Conserved hypothetical protein. |  | 15.34 | 11.30 | 5.68 |  |  |
| Os.30528.1.S1_at | Os08g0412800 Protein of unknown function DUF1262 family protein. |  | 14.64 | 16.47 | 11.46 |  | 4.32 |
| Os.10682.1.S1_at | Os08g05960 expressed protein |  | 13.80 | 55.24 | 6.24 |  |  |
| Os.49485.1.S1_at | Os09g0426000 Protein of unknown function DUF6 domain containing protein. |  | 11.44 | 26.41 | 12.38 | 3.26 | 4.54 |
| Os.57476.1.A1_x_at | Unknown |  | 7.96 | 15.94 | 5.07 | 3.00 |  |
| Os.4963.1.S1_at | Os10g0471000 Protein of unknown function DUF810 family protein. |  | 7.18 | 11.23 | 7.62 |  |  |
| Os.23172.1.S1_x_at | Os07g02940 expressed protein |  | 6.11 | 5.54 | 6.87 |  | 0.33 |
| OsAffx.3147.1.S1_s_at | Os03g0158600 Conserved hypothetical protein. |  | 5.88 | 8.52 | 5.28 | 2.31 |  |
| Os.41841.1.S1_at | Os01g0719300 Sulfate transporter 3.1 (AST12) (AtST1). | 3.19 | 12.24 | 11.45 | 6.40 |  |  |
| Os.33968.1.S1_at | Os01g0385400 C4-dicarboxylate transporter/malic acid transport protein family protein. |  | 8.55 | 42.91 | 7.17 | 4.36 | 2.27 |
| Os.23143.1.S1_at | Os09g31478 auxin hydrogen symporter, putative, expressed |  | 10.34 | 20.43 | 5.57 |  |  |
| Os.52648.1.S1_at | Os04g0435100 Amino acid/polyamine transporter I |  | 6.22 | 7.51 | 6.74 |  |  |
| Os.11608.1.S1_at | Os01g0741900 Auxin-responsive protein IAA26 |  | 7.09 | 14.21 | 5.56 | 0.39 | 0.41 |
| Os.14052.1.S1_at | Os03g0654700 RmlC-like cupin family protein. | 2.93 | 19.86 | 26.48 | 7.80 |  |  |
| Os.17286.1.S1_at | Os03g0327800 NAC-domain containing protein 29 |  | 15.93 | 51.80 | 9.11 | 2.54 |  |
| Os.17446.2.S1_at | Os04g0571300 Cyclin-like F-box domain containing protein. | 3.50 | 14.55 | 23.68 | 9.09 | 4.67 | 4.12 |
| Os.49023.1.S1_x_at | Os03g0348900 CHY zinc finger domain containing protein. |  | 12.34 | 22.90 | 8.20 | 3.11 |  |
| Os.24913.1.A1_at | Os04g0571200 Zn-finger, RING domain containing protein. | 2.03 | 7.29 | 6.50 | 9.75 | 2.80 | 3.18 |
| Os.11786.2.S1_x_at | Os01g0264000 Zn-finger, Dof type domain containing protein. |  | 7.06 | 5.24 | 6.42 |  | 3.29 |
| Os.27875.1.S1_at | Os10g0416800 Class III chitinase. | 2.77 | 15.21 | 33.00 | 8.46 |  |  |
| Os.25497.1.S1_at | Os11g0700900 Class III chitinase homologue (OsChib3H-b). | 2.36 | 9.10 | 42.05 | 6.03 |  |  |
| Os.11935.1.S1_at | Os02g0526400 ERD1 protein, chloroplast precursor. |  | 28.67 | 34.81 | 7.95 | 2.03 |  |
| OsAffx.12547.1.S1_at | Os02g0712700 Legume lectin, beta domain containing protein. | 4.84 | 9.57 | 14.73 | 7.16 |  |  |
| Os.28139.1.S1_at | Os01g0839900 Osmotin-like protein precursor. |  | 15.05 | 13.96 | 9.30 |  |  |
| Os.459.1.S1_at | Os03g0663500 Thaumatin |  | 5.75 | 9.66 | 20.40 | 4.00 |  |
| Os.32357.1.S1_at | Os07g0664600 Short-chain dehydrogenase family protein | 2.68 | 39.80 | 52.15 | 25.05 |  |  |
| s.45997.1.S1_x_at | Os01g0644000 Twin-arginine translocation pathway signal domain containing protein. |  | 18.57 | 35.11 | 16.82 | 4.11 | 3.26 |
| Os.34139.1.S1_at | Os08g0141400 FAD-dependent pyridine nucleotide-disulphide oxidoreductase domain containing protein. |  | 6.09 | 10.14 | 5.16 | 4.65 | 2.73 |
| Os.39617.1.S1_at | Os09g0472100 ABC transporter. |  | 6.92 | 7.42 | 6.91 |  |  |
| Os.28435.1.S1_a_at | Os10g0558700 2OG-Fe(II) oxygenase domain containing protein. |  | 7.50 | 16.38 | 7.27 |  |  |
| Os.11682.1.S1_at | Os01g0800500 Metallophosphoesterase domain containing protein. |  | 12.17 | 13.77 | 26.35 |  |  |
| Os.52005.1.S1_at | Os02g0783700 Lysine-ketoglutarate reductase/saccharopine dehydrogenase bifunctional enzyme. |  | 47.45 | 87.46 | 11.60 | 3.04 |  |
| Os.22169.4.S1_x_at | Os06g0726400 Branching enzyme-I precursor (Starch-branching enzyme I). |  | 8.26 | 9.05 | 5.60 |  |  |
| Os.38110.1.S1_at | Os10g0521000 Glycoside hydrolase, family 37 protein. |  | 43.29 | 41.94 | 8.62 | 2.56 | 2.47 |
| OsAffx.14113.1.S1_at | Os04g0409900 Plant neutral invertase family protein. | 3.59 | 11.75 | 5.93 | 7.08 | 3.01 | 3.17 |
| Os.50953.1.S1_at | Os06g0713800 Alpha-amylase isozyme 2A precursor (EC 3.2.1.1) |  | 9.04 | 9.60 | 5.72 |  |  |
| Os.6034.1.S1_at | Os01g0192900 ACC synthase (EC 4.1.1.14) (Fragment). |  | 29.04 | 88.05 | 24.50 |  |  |
| Os.23468.1.A1_at | Os09g0452900 Glycosyl transferase, family 31 protein. |  | 15.18 | 19.08 | 6.62 |  |  |
| Os.51929.1.S1_at | Os10g0450900 Glycine-rich cell wall structural protein 2 precursor. | 0.42 | 6.19 | 14.80 | 6.70 |  |  |
