# Supplementary material for: Genome-wide temporal-spatial gene expression profiling of drought responsiveness in rice
Source: BMC Genomics. 2011 Mar 16;12:149. doi: 10.1186/1471-2164-12-149 (PMC3070656; doi:10.1186/1471-2164-12-149)
Supplement: Additional file 11 — Leaf specific down-regulated genes under drought stress. Excel file containing all specific down-regulated genes by drought in leaf. [file 1471-2164-12-149-S11.DOC]

**Additional file 11**. Leave specific down-regulated genes under drought stress

| **Gene ID** | **Annotation** | **BP** | **BL** | **PL** | **TL** | **PR** | **TR** |
| --- | --- | --- | --- | --- | --- | --- | --- |
| Os.7016.1.S1_at | Os03g0304800 Conserved hypothetical protein. |  | 0.20 | 0.07 | 0.11 |  | 0.43 |
| Os.32482.2.S1_x_at | Os01g0805300 Conserved hypothetical protein. |  | 0.20 | 0.03 | 0.17 |  |  |
| Os.9093.1.S1_at | Os06g0152600 Non-protein coding transcript, uncharacterized transcript. | 0.24 | 0.19 | 0.05 | 0.06 | 0.47 | 0.26 |
| Os.34118.1.S1_at | Os10g0522800 Conserved hypothetical protein. |  | 0.18 | 0.11 | 0.19 |  | 0.33 |
| Os.39297.1.S1_at | Os10g0206500 Conserved hypothetical protein. | 0.42 | 0.17 | 0.05 | 0.16 |  | 0.35 |
| Os.2376.1.S1_at | Os03g0283200 IN2-1 protein. |  | 0.15 | 0.13 | 0.18 | 3.13 | 2.82 |
| Os.16805.1.S1_x_at | Os05g0516100 Conserved hypothetical protein. |  | 0.14 | 0.17 | 0.06 |  |  |
| Os.27602.1.S1_at | Os03g0118200 Conserved hypothetical protein. |  | 0.13 | 0.13 | 0.13 |  |  |
| OsAffx.28164.1.S1_at | Unknown |  | 0.11 | 0.16 | 0.07 |  |  |
| Os.28216.3.S1_x_at | Unknown |  | 0.11 | 0.04 | 0.18 | 0.24 |  |
| Os.11330.1.S2_at | Unknown |  | 0.11 | 0.07 | 0.14 |  |  |
| Os.20516.1.S1_at | Unknown | 0.45 | 0.11 | 0.13 | 0.18 |  |  |
| Os.53467.1.S1_at | Os03g0342100 Hypothetical protein. | 0.22 | 0.10 | 0.11 | 0.08 |  |  |
| Os.51753.1.A1_at | Os12g0538700 AT.I.24-1 protein (Fragment). |  | 0.10 | 0.07 | 0.19 |  |  |
| Os.35815.1.S1_at | Os01g0111100 Peptidyl-prolyl cis-trans isomerase | 0.29 | 0.09 | 0.02 | 0.16 | 0.42 | 0.43 |
| Os.5516.1.S1_at | Unknown |  | 0.08 | 0.05 | 0.14 |  | 0.25 |
| OsAffx.25846.1.S1_s_at | Os04g0120100 Conserved hypothetical protein. |  | 0.07 | 0.08 | 0.10 |  |  |
| Os.12410.3.S1_x_at | Unknown |  | 0.07 | 0.15 | 0.17 |  |  |
| Os.6767.1.S1_at | Os08g0269700 Conserved hypothetical protein. | 0.35 | 0.07 | 0.02 | 0.13 |  |  |
| Os.56918.1.S1_at | Os03g0839800 Conserved hypothetical protein. |  | 0.07 | 0.08 | 0.14 |  |  |
| Os.10522.2.S1_at | Os05g0291700 Conserved hypothetical protein. | 0.29 | 0.06 | 0.04 | 0.15 |  |  |
| Os.17196.1.A1_s_at | Os11g0118600 Protein of unknown function DUF1191 family protein | 0.24 | 0.06 | 0.04 | 0.09 | 0.43 | 0.46 |
| Os.33661.1.S1_at | Os06g0174700 Hypothetical protein. |  | 0.06 | 0.03 | 0.10 |  |  |
| Os.4683.2.S1_at | Os01g0611000 Protein of unknown function DUF642 family protein. |  | 0.05 | 0.02 | 0.03 | 0.23 | 0.21 |
| Os.4404.1.S1_s_at | Os06g0109200 Conserved hypothetical protein. | 0.28 | 0.04 | 0.09 | 0.16 |  |  |
| Os.2759.1.S1_s_at | Os10g0573700 Mitochondrial substrate carrier family protein | 0.22 | 0.18 | 0.02 | 0.14 | 0.43 | 0.38 |
| Os.34767.2.S1_s_at | Os03g0226400 Cation diffusion facilitator 8. |  | 0.10 | 0.06 | 0.15 | 0.36 | 0.24 |
| Os.50789.1.S1_x_at | Os07g0134000 Amino acid permease 6 (celluar component). | 0.34 | 0.07 | 0.09 | 0.20 |  | 0.35 |
| Os.12018.1.S1_at | Os07g0619400 Calcium-binding EF-hand domain containing protein. |  | 0.14 | 0.04 | 0.20 |  |  |
| Os.35718.1.S1_at | Os12g0581900 Zn-finger, C2H2 type domain containing protein. |  | 0.13 | 0.10 | 0.18 |  | 0.49 |
| Os.10830.1.S1_at | Os08g0524800 TA1 protein (Fragment, similar to HLH). |  | 0.12 | 0.12 | 0.13 | 0.34 | 0.31 |
| Os.31716.2.A1_at | Os06g0105200 GRAS transcription factor family protein. | 0.21 | 0.08 | 0.04 | 0.18 |  | 0.38 |
| Os.52264.1.S1_at | Os05g0317900 Resistance protein candidate (Fragment). |  | 0.03 | 0.02 | 0.04 |  |  |
| Os.3121.1.S1_at | Os10g0416100 Class III chitinase RCB4 (EC 3.2.1.14). |  | 0.19 | 0.17 | 0.19 |  | 0.24 |
| Os.17416.1.S1_at | Os01g0279400 Major facilitator superfamily antiporter. |  | 0.15 | 0.07 | 0.10 | 5.82 |  |
| OsAffx.3354.1.S1_at | Os03g0400700 Late embryogenesis abundant protein 3 family protein. |  | 0.14 | 0.07 | 0.15 |  |  |
| Os.11611.1.S1_at | Os02g13350 nudix hydrolase 8, putative, expressed |  | 0.14 | 0.06 | 0.15 |  |  |
| Os.15729.2.S1_at | Os04g0585200 Glutamate receptor 3.3 precursor (Ligand-gated ion channel 3.3). |  | 0.18 | 0.02 | 0.15 |  |  |
| OsAffx.27482.2.S1_s_at | Os06g0165500 Protein kinase family protein. |  | 0.17 | 0.07 | 0.19 |  |  |
| Os.27580.1.S1_at | Os07g0558300 Inositol monophosphatase family protein. |  | 0.15 | 0.02 | 0.09 |  |  |
| Os.49827.1.S1_at | Os11g0208900 Leucine rich repeat containing protein kinase. |  | 0.11 | 0.07 | 0.11 |  |  |
| Os.54602.1.S1_s_at | Os04g0401000 Heavy metal transport/detoxification protein domain containing protein. |  | 0.18 | 0.14 | 0.03 |  |  |
| Os.14324.1.S1_s_at | Os04g0400000 Heavy metal transport/detoxification protein domain containing protein. |  | 0.11 | 0.01 | 0.09 |  |  |
| Os.15355.1.S1_x_at | Os02g0240300 Plant peroxidase family protein. |  | 0.09 | 0.13 | 0.11 |  |  |
| Os.22884.1.A1_at | Os01g0378100 Peroxidase precursor (EC 1.11.1.7). | 0.48 | 0.08 | 0.07 | 0.07 |  |  |
| Os.14368.1.S1_at | Os08g0382400 Peptidyl-prolyl cis-trans isomerase | 0.43 | 0.15 | 0.02 | 0.18 |  |  |
| Os.12127.1.S1_at | Os09g0481200 Photosystem I reaction center subunit V (PSI-G) |  | 0.12 | 0.03 | 0.19 |  |  |
| OsAffx.6488.1.S1_at | Os09g0528700 Cytochrome p450 (CYP78A9). |  | 0.09 | 0.12 | 0.17 |  |  |
| Os.27609.1.A1_x_at | Os04g0457000 Chlorophyll a/b-binding protein CP24 |  | 0.07 | 0.02 | 0.18 |  |  |
| Os.12363.1.S1_at | Os04g0678700 Protochlorophyllide reductase A | 0.28 | 0.06 | 0.04 | 0.18 |  |  |
| Os.17880.1.S1_a_at | Os08g0509200 Amygdalin hydrolase isoform AH I precursor (EC 3.2.1.117). |  | 0.07 | 0.07 | 0.19 |  | 2.71 |
| Os.56245.1.S1_at | Os09g0373200 Strictosidine synthase family protein. |  | 0.15 | 0.02 | 0.08 |  |  |
| Os.35510.1.S1_at | Os02g0102400 Ribosomal protein S5, C-terminal domain containing protein |  | 0.15 | 0.09 | 0.18 |  |  |
| Os.26871.1.S1_at | Os09g0564000 Peptidase C1A, papain family protein. |  | 0.05 | 0.05 | 0.08 |  |  |
| Os.19118.2.S1_x_at | Os06g0531600 Lipolytic enzyme, G-D-S-L family protein. |  | 0.15 | 0.10 | 0.07 | 4.53 | 2.13 |
| Os.56997.1.S1_at | Os08g0485900 Haloacid dehalogenase-like hydrolase domain containing protein. | 0.36 | 0.12 | 0.01 | 0.14 |  |  |
| Os.8382.1.S1_x_at | Os02g0647900 Aldehyde dehydrogenase domain containing protein. |  | 0.11 | 0.02 | 0.08 |  |  |
| Os.11714.1.S1_at | Os02g0771700 Glycoside hydrolase, family 17 protein. | 0.41 | 0.18 | 0.07 | 0.14 |  | 0.47 |
| Os.8390.1.S1_s_at | Os07g0575500 Glycoside hydrolase, family 20 protein. | 0.27 | 0.06 | 0.04 | 0.19 |  |  |
| Os.28983.1.S1_at | Os07g0162700 Esterase/lipase/thioesterase domain containing protein. |  | 0.08 | 0.10 | 0.02 |  |  |
| Os.12410.1.S1_a_at | Os01g0974200 RicMT (Metallothionein-like protein). |  | 0.04 | 0.15 | 0.15 |  |  |
| Os.50280.1.S1_at | Os04g0530900 Glycosyl transferase, family 8 protein. | 0.22 | 0.17 | 0.10 | 0.13 | 0.38 | 0.37 |
| Os.50825.1.S1_s_at | Os05g0477600 Alpha-expansin OsEXPA4. |  | 0.07 | 0.07 | 0.03 |  |  |
| Os.22783.1.S1_s_at | Os05g0382600 Annexin family protein. |  | 0.19 | 0.03 | 0.19 |  |  |
