# Supplementary material for: Genome-wide temporal-spatial gene expression profiling of drought responsiveness in rice
Source: BMC Genomics. 2011 Mar 16;12:149. doi: 10.1186/1471-2164-12-149 (PMC3070656; doi:10.1186/1471-2164-12-149)
Supplement: Additional file 12 — Root-specific up-regulated genes under drought stress. Excel file containing all specific up-regulated genes by drought in roots. [file 1471-2164-12-149-S12.DOC]

**Additional file 12. Roots specific up-regulated genes under drought stress**

| **Gene ID** | **Annotation** | **BP** | **BL** | **PL** | **TL** | **PR** | **TR** |
| --- | --- | --- | --- | --- | --- | --- | --- |
| Os.51749.1.S1_at | unknown |  |  | 4.69 |  | 71.05 | 6.00 |
| Os.54420.1.S1_x_at | Os01g0276900 Conserved hypothetical protein. |  |  |  |  | 44.75 | 13.20 |
| Os.38205.1.S1_at | Os01g0786800 Conserved hypothetical protein. | 3.56 | 0.35 | 0.34 |  | 28.93 | 23.16 |
| Os.52136.1.S1_at | Os03g0808900 Seed imbibition protein. | 4.74 |  |  |  | 19.94 | 9.64 |
| OsAffx.25534.1.S1_at | unknown |  |  |  |  | 17.70 | 11.36 |
| Os.7527.1.S1_at | Os01g0102300 Conserved hypothetical protein. |  | 0.41 | 0.08 |  | 16.02 | 9.97 |
| Os.26490.1.S1_at | Os10g0499600 Conserved hypothetical protein. | 2.32 |  |  |  | 15.00 | 10.66 |
| Os.54736.1.S1_at | Os02g0768000 Conserved hypothetical protein. |  |  |  |  | 14.75 | 9.23 |
| OsAffx.25498.1.S1_at | Os03g0625300 Quinonprotein alcohol dehydrogenase-like domain containing protein. |  |  |  | 0.18 | 14.38 | 13.01 |
| Os.5518.1.S1_at | Os12g0575000 Protein of unknown function DUF1118 family protein. |  |  | 0.17 |  | 14.36 | 15.26 |
| OsAffx.23143.1.S1_x_at | unknown |  |  |  |  | 13.90 | 13.12 |
| Os.5403.1.S1_at | unknown |  |  |  |  | 12.77 | 9.06 |
| Os.46649.1.S1_x_at | Os10g0499800 Fertility restorer.; Os10g0499600 Conserved hypothetical protein. | 2.35 |  |  |  | 11.73 | 9.68 |
| Os.46498.1.S1_at | Os10g0190500 Protein of unknown function DUF594 family protein. |  | 0.39 |  |  | 11.41 | 9.31 |
| Os.54595.1.S1_at | Os06g0302000 Hypothetical protein. |  |  |  |  | 11.03 | 29.12 |
| Os.23101.1.A1_at | Os04g0630300 Conserved hypothetical protein. |  | 4.07 |  |  | 10.54 | 10.17 |
| Os.19366.1.S1_a_at | Os03g0578300 Hypothetical protein. | 4.03 | 2.88 |  | 2.20 | 9.51 | 6.40 |
| Os.16218.1.S1_at | Os06g0498800 MOTHER of FT and TF1 protein. | 3.32 |  | 2.06 | 2.78 | 9.38 | 13.53 |
| Os.38283.1.S1_a_at | Os05g0495300 Hypothetical protein. | 3.32 | 4.78 | 2.65 |  | 9.26 | 5.97 |
| Os.7343.1.S1_at | Os07g0118300 Conserved hypothetical protein. | 2.58 |  | 3.36 |  | 9.04 | 5.16 |
| OsAffx.12600.1.S1_at | Os02g0753900 Conserved hypothetical protein. |  |  |  |  | 8.74 | 9.13 |
| Os.53108.1.S1_at | Os02g0828100 Conserved hypothetical protein. |  |  |  | 0.48 | 8.19 | 5.41 |
| Os.57197.1.S1_at | unknown | 4.82 |  |  |  | 7.78 | 5.09 |
| Os.23695.2.S1_at | unknown | 3.71 | 2.13 | 2.31 |  | 7.76 | 5.71 |
| OsAffx.3968.1.S1_at | Os04g0386700 Conserved hypothetical protein. | 2.64 |  | 3.04 |  | 7.67 | 6.70 |
| Os.49964.1.S2_at | unknown | 4.19 |  | 3.81 | 2.91 | 7.67 | 5.13 |
| Os.4509.1.S1_x_at | Os06g0114400 Conserved hypothetical protein. | 2.35 |  |  |  | 7.46 | 5.94 |
| Os.11668.1.S1_at | Os03g0306900 TENA/THI-4 protein domain containing protein. | 2.97 | 2.67 | 2.64 | 3.98 | 7.16 | 6.36 |
| Os.20333.1.S1_at | Os02g0799600 Conserved hypothetical protein. | 4.23 |  |  |  | 6.26 | 7.90 |
| Os.48545.1.S1_at | Os04g0630400 Conserved hypothetical protein. |  |  | 0.48 |  | 6.23 | 6.26 |
| Os.56345.1.S1_at | Os05g0339300 Hypothetical protein. |  |  |  |  | 5.85 | 5.02 |
| Os.27482.1.A1_at | Os05g0519300 Uncharacterized plant-specific protein DUF506 domain containing protein. |  | 4.73 |  |  | 5.82 | 5.11 |
| OsAffx.24317.1.S1_x_at | unknown |  | 4.69 |  | 4.06 | 5.74 | 7.34 |
| Os.28399.1.S3_at | Os02g0129700 Hypothetical protein. | 4.53 | 3.79 | 2.20 | 2.07 | 5.19 | 5.47 |
| Os.27640.1.A1_s_at | Os12g0552800 Hypothetical protein. | 2.79 | 4.77 | 2.33 | 2.89 | 5.15 | 5.73 |
| Os.14537.1.S1_at | Os09g0469300 Plastocyanin-like domain containing protein. | 0.45 |  | 0.17 | 0.37 | 43.53 | 25.60 |
| Os.8593.1.S1_at | Os04g0631100 General substrate transporter family protein. |  |  | 2.61 |  | 11.68 | 15.69 |
| Os.56004.1.S1_at | Os03g0347500 MtN3-like protein. |  |  |  |  | 128.30 | 135.19 |
| Os.10031.1.S1_at | Os05g0426000 MtN3 and saliva related transmembrane protein family protein. |  |  |  |  | 7.72 | 6.92 |
| Os.23778.1.S1_at | Os02g0527300 Heat shock transcription factor 31 (Fragment). | 3.79 |  | 2.58 |  | 6.90 | 6.96 |
| Os.54299.1.S1_at | Os06g0211200 AREB1 |  |  |  |  | 19.35 | 13.86 |
| Os.55096.1.S1_at | Os06g0258000 Typical P-type R2R3 Myb protein (Fragment). |  | 4.78 | 4.79 | 4.64 | 33.49 | 15.39 |
| Os.47717.1.A1_s_at | Os01g0866500 Soluble inorganic pyrophosphatase (EC 3.6.1.1) (Pyrophosphate phospho- hydrolase) (PPase). |  | 0.47 | 0.24 |  | 29.26 | 28.11 |
| Os.2235.1.S1_a_at | Os12g0156200 DNA-binding factor of bZIP class. | 4.23 |  | 4.43 |  | 20.85 | 6.56 |
| OsAffx.3135.1.S1_at | Os03g0142600 Myb, DNA-binding domain containing protein. |  |  |  |  | 12.97 | 9.30 |
| Os.49829.1.S1_at | Os02g0618400 MYB8 protein. |  |  | 4.72 |  | 8.32 | 6.37 |
| Os.35808.1.S1_at | Os01g0606900 Heat shock protein DnaJ, N-terminal domain containing protein. | 2.18 |  |  |  | 91.75 | 39.37 |
| Os.12234.2.S1_s_at | Os06g0219500 Low molecular weight heat shock protein precursor. | 3.12 |  | 2.01 | 4.56 | 35.36 | 7.20 |
| OsAffx.31356.1.S1_at | Os11g37880 stripe rust resistance protein Yr10, putative, expressed |  |  |  |  | 8.21 | 10.69 |
| Os.15989.1.S1_at | Os03g0749900 Conserved hypothetical protein.; Os03g0750000 Universal stress protein (Usp) family protein. | 3.68 |  |  |  | 7.31 | 6.04 |
| Os.55637.1.S1_at | Os05g0526700 Harpin-induced 1 domain containing protein. |  |  |  |  | 7.88 | 9.75 |
| Os.7992.1.S1_a_at | Os01g0200700 Metallothionein-like protein type 3 (MT-3). |  |  |  |  | 5.37 | 5.73 |
| Os.51460.1.S1_at | Os02g0537000 Pectinesterase inhibitor domain containing protein. | 3.01 |  |  |  | 20.61 | 5.20 |
| OsAffx.11003.2.S1_at | Os01g0206300 OsPK4. |  |  |  |  | 20.26 | 11.62 |
| OsAffx.18298.1.S1_at | Os10g0337400 Protein kinase family protein. |  |  |  |  | 19.18 | 18.62 |
| Os.9303.1.S1_at | Os02g0689900 TGF-beta receptor, type I/II extracellular region family protein. | 0.43 |  | 0.26 |  | 7.08 | 9.48 |
| Os.56528.1.S1_at | Os05g0501400 Receptor-like protein kinase 5. |  |  | 4.54 | 4.38 | 6.21 | 5.53 |
| OsAffx.25764.1.S1_at | Os03g0805800 Rieske [2Fe-2S] region domain containing protein. |  |  |  |  | 6.67 | 7.34 |
| OsAffx.10756.1.S1_x_at | Os02g0700400 Alternative oxidase AOX1. | 3.26 | 3.01 |  |  | 8.96 | 9.95 |
| OsAffx.25182.1.S1_s_at | Os03g0332500 60S ribosomal protein L10 (QM protein homolog). |  |  |  |  | 43.18 | 8.51 |
| Os.13735.1.S1_at | Os02g0828200 TPR-like domain containing protein. |  |  |  |  | 6.89 | 14.28 |
| Os.4759.1.S1_at | Os09g0367700 GST6 protein (EC 2.5.1.18). |  |  |  |  | 6.71 | 7.14 |
| Os.21842.1.S1_at | Os10g0530200 Glutathione S-transferase, C-terminal domain containing protein. |  |  |  |  | 5.74 | 5.57 |
| Os.25325.1.A1_at | Os06g0705500 Ubiquitin system component Cue domain containing protein. |  | 2.12 | 2.28 | 2.48 | 5.12 | 7.04 |
| Os.3408.2.S1_at | Os01g0866500 Soluble inorganic pyrophosphatase | 2.26 |  |  |  | 91.51 | 79.34 |
| Os.5739.1.S1_at | Os05g0568100 Nitrogen-fixing NifU-like, N-terminal domain containing protein. | 3.26 | 2.72 | 2.04 |  | 36.23 | 37.43 |
| Os.45923.1.S1_at | Os01g0556700 Dicarboxylate transporter. |  |  | 0.16 |  | 34.96 | 48.91 |
| Os.11851.1.S1_at | Os03g0218400 Hexose transporter. |  | 2.79 | 2.79 | 3.39 | 13.58 | 10.19 |
| Os.24545.2.S1_x_at | Os03g0416500 Glucosamine/galactosamine-6-phosphate isomerase domain containing protein. | 4.65 |  |  |  | 12.46 | 7.25 |
| Os.12703.1.S1_at | Os03g0197100 Sugar transporter protein. |  | 0.49 | 0.06 |  | 10.19 | 23.67 |
| Os.3408.1.A2_a_at | Os01g0866400 Fructose-1,6-bisphosphatase (EC 3.1.3.11) (Fragment). |  | 0.35 | 0.07 |  | 8.95 | 9.34 |
| OsAffx.11499.1.S1_at | Os01g0660900 Phosphoglycerate/bisphosphoglycerate mutase family protein. | 3.40 |  |  |  | 8.50 | 7.57 |
| Os.46695.1.S1_at | Os10g0361900 Lipase/lipooxygenase, PLAT/LH2 domain containing protein. |  |  |  |  | 12.18 | 33.61 |
| Os.53400.1.S1_at | Os11g0609600 14-3-3 protein (carbohydrate and nitrogen metabolism). |  |  |  |  | 12.25 | 13.33 |
| Os.1853.1.S1_at | Os08g0189100 Germin-like protein precursor. |  |  |  |  | 7.35 | 21.06 |
| Os.6297.1.S1_at | Os02g0681000 MscS Mechanosensitive ion channel family protein | 4.70 | 2.95 | 2.15 |  | 12.87 | 14.91 |
| Os.4223.1.S1_s_at | Os06g0168700 Prolin rich protein. |  |  |  |  | 8.04 | 7.17 |
