# Supplementary material for: Genome-wide temporal-spatial gene expression profiling of drought responsiveness in rice
Source: BMC Genomics. 2011 Mar 16;12:149. doi: 10.1186/1471-2164-12-149 (PMC3070656; doi:10.1186/1471-2164-12-149)
Supplement: Additional file 13 — Root-specific down-regulated genes under drought stress. Excel file containing all specific down-regulated genes by drought in roots [file 1471-2164-12-149-S13.DOC]

**Additional file 13. Roots specific down-regulated genes under drought stress.**

| **Gene ID** | **Annotation** | **BP** | **BL** | **PL** | **TL** | **PR** | **TR** |
| --- | --- | --- | --- | --- | --- | --- | --- |
| OsAffx.20160.1.S1_x_at | unknown |  |  |  |  | 0.20 | 0.13 |
| Os.26894.1.A1_x_at | unknown |  |  |  |  | 0.20 | 0.19 |
| OsAffx.31010.1.S1_at | Os11g16970 carnitine racemase like protein, putative, expressed |  |  |  |  | 0.20 | 0.17 |
| Os.17985.1.S1_s_at | Os03g0776400 Conserved hypothetical protein. |  |  |  |  | 0.20 | 0.19 |
| OsAffx.13670.1.S1_at | Os04g0134900 Hypothetical protein. |  |  |  |  | 0.19 | 0.13 |
| Os.54275.1.S1_at | Os06g0178700 Isopenicillin N synthase family protein. |  |  |  |  | 0.19 | 0.15 |
| Os.27713.1.A1_at | Os11g0591200 Beta-ketoacyl-CoA-synthase. | 0.24 | 2.21 |  |  | 0.19 | 0.15 |
| OsAffx.20160.1.S1_at | unknown |  |  |  |  | 0.18 | 0.13 |
| Os.9700.1.S1_at | Os08g0377500 Protein of unknown function |  |  |  |  | 0.17 | 0.14 |
| Os.55754.1.S1_at | Os12g0149000 Hypothetical protein. | 0.37 | 3.11 |  |  | 0.17 | 0.09 |
| Os.34496.1.S1_at | Os03g0830500 PGPS/D12. | 21.72 |  | 21.56 |  | 0.17 | 0.03 |
| Os.10091.1.S1_at | Os03g0302800 Conserved hypothetical protein. | 0.43 |  |  |  | 0.17 | 0.19 |
| Os.8682.1.S1_at | Os10g0169200 Conserved hypothetical protein. |  |  |  |  | 0.17 | 0.09 |
| OsAffx.27693.1.S1_at | Os06g18850 expressed protein |  |  |  |  | 0.17 | 0.17 |
| Os.55386.1.A1_at | unknown |  |  |  |  | 0.16 | 0.10 |
| Os.28778.1.S1_at | Os01g0826800 Conserved hypothetical protein. |  | 0.34 | 0.24 | 0.40 | 0.16 | 0.08 |
| OsAffx.2803.1.S1_at | unknown |  |  |  |  | 0.16 | 0.13 |
| Os.11231.1.S1_at | Os05g0337200 Conserved hypothetical protein. |  |  |  |  | 0.16 | 0.08 |
| Os.9072.1.S1_at | Os11g0496500 AT.I.24-5 protein (Fragment). | 0.24 |  |  | 0.35 | 0.16 | 0.12 |
| Os.34624.2.S1_s_at | Os03g0582000 Conserved hypothetical protein. |  | 0.43 |  |  | 0.16 | 0.15 |
| OsAffx.30934.1.S1_at | unknown |  |  |  |  | 0.16 | 0.14 |
| Os.34873.1.S1_at | Os07g0659300 Conserved hypothetical protein. |  |  | 0.23 | 0.31 | 0.16 | 0.14 |
| Os.36346.2.S1_at | Os01g0895300 Conserved hypothetical protein. |  |  |  |  | 0.15 | 0.02 |
| Os.52778.1.S1_at | Os02g0741700 Conserved hypothetical protein. |  |  | 0.34 |  | 0.15 | 0.07 |
| Os.20292.1.S1_at | Os04g0587400 Protein of unknown function | 4.16 | 0.34 | 0.33 |  | 0.15 | 0.19 |
| Os.20242.1.S1_at | Os11g0482200 Hypothetical protein. |  |  |  |  | 0.15 | 0.11 |
| Os.46476.1.S1_at | Os10g0454300 Eggshell protein family protein. |  |  |  |  | 0.15 | 0.11 |
| Os.8503.1.S1_at | Os06g0591200 Conserved hypothetical protein. |  |  | 0.46 |  | 0.15 | 0.11 |
| OsAffx.14165.1.S1_at | Os04g35750 expressed protein |  |  |  |  | 0.15 | 0.15 |
| OsAffx.31409.1.S1_s_at | unknown | 10.31 | 3.32 | 4.58 |  | 0.15 | 0.19 |
| Os.37062.1.S1_at | Os01g0389200 Protein of unknown function |  |  |  |  | 0.14 | 0.07 |
| Os.56891.1.S1_at | Os03g0412400 Conserved hypothetical protein. |  |  |  | 2.07 | 0.14 | 0.14 |
| OsAffx.30129.1.S1_at | unknown |  |  |  |  | 0.13 | 0.13 |
| OsAffx.6894.1.S1_x_at | Os10g0518000 Protein of unknown function | 0.25 |  |  |  | 0.13 | 0.10 |
| Os.6152.1.S1_at | Os05g0181700 Hypothetical protein. |  |  |  |  | 0.13 | 0.18 |
| Os.8576.1.S1_at | Os12g0623600 Conserved hypothetical protein. | 2.10 |  |  | 0.48 | 0.13 | 0.16 |
| Os.7095.1.S1_at | Os04g0581100 Isopenicillin N synthase family protein. |  |  |  |  | 0.13 | 0.15 |
| Os.51420.1.S1_at | Os04g0645500 Conserved hypothetical protein. |  |  |  |  | 0.13 | 0.17 |
| OsAffx.28981.1.S1_at | Os08g0108700 Conserved hypothetical protein. |  |  |  |  | 0.13 | 0.07 |
| Os.11337.1.S1_at | Os10g0454200 Hypothetical protein. |  |  |  |  | 0.13 | 0.08 |
| OsAffx.17472.1.S1_at | unknown |  |  |  |  | 0.13 | 0.07 |
| Os.56048.1.S1_s_at | Os01g0916100 Conserved hypothetical protein. |  |  |  |  | 0.13 | 0.04 |
| Os.54416.1.S1_at | Os02g0623300 Conserved hypothetical protein. | 6.83 |  |  |  | 0.13 | 0.06 |
| Os.53581.1.S1_at | Os09g0542900 Conserved hypothetical protein. |  | 4.12 | 3.92 |  | 0.12 | 0.12 |
| OsAffx.28946.1.S1_at | Os07g47660 expressed protein |  |  |  |  | 0.12 | 0.11 |
| Os.9686.1.S1_at | Os06g0643700 Hly-III related proteins family protein. | 0.42 |  |  | 0.35 | 0.12 | 0.14 |
| Os.51345.1.S1_at | Os05g0181300 Hypothetical protein. | 0.44 |  |  |  | 0.12 | 0.09 |
| OsAffx.13593.1.S1_at | unknown |  |  |  |  | 0.12 | 0.12 |
| Os.4453.1.S1_at | Os06g0125200 Protein of unknown function | 2.29 | 0.43 | 0.40 |  | 0.11 | 0.12 |
| Os.47598.1.S1_at | Os01g12190 expressed protein |  |  | 0.41 |  | 0.11 | 0.07 |
| Os.46565.1.S1_at | Os10g0450000 Plant protein of unknown function | 0.27 |  |  |  | 0.11 | 0.11 |
| Os.54950.1.S1_at | Os03g0219100 Cellular retinaldehyde-binding |  |  |  |  | 0.11 | 0.15 |
| OsAffx.26878.1.S1_at | unknown |  |  |  |  | 0.10 | 0.11 |
| Os.7539.1.S1_at | Os03g0124500 Hypothetical protein. | 0.42 | 0.40 | 0.21 | 2.25 | 0.10 | 0.05 |
| Os.23059.1.S1_s_at | unknown |  |  |  |  | 0.10 | 0.08 |
| Os.23606.1.S1_at | Os01g0389700 Protein of unknown function |  |  | 3.38 |  | 0.10 | 0.06 |
| Os.12788.1.S1_at | Os01g0210600 Protein of unknown function |  |  |  |  | 0.10 | 0.17 |
| Os.11150.1.S1_at | Os03g0439700 Protein of unknown function |  | 0.38 | 0.32 | 0.25 | 0.10 | 0.04 |
| Os.50198.1.S1_at | Os12g0592900 Hypothetical protein. |  | 7.91 | 35.50 |  | 0.09 | 0.13 |
| Os.56329.1.S1_at | unknown |  |  |  |  | 0.09 | 0.07 |
| Os.53438.1.S1_at | unknown |  |  |  |  | 0.09 | 0.14 |
| Os.27440.1.S1_at | Os01g0522400 ARM repeat fold domain containing protein. |  |  |  |  | 0.09 | 0.10 |
| Os.31233.2.S1_at | Os01g0266400 Conserved hypothetical protein. | 0.42 |  |  |  | 0.09 | 0.11 |
| OsAffx.30442.1.S1_at | unknown |  |  |  |  | 0.09 | 0.02 |
| Os.10441.1.S1_at | unknown |  |  |  |  | 0.09 | 0.12 |
| Os.9180.1.S1_at | Os10g0454500 Eggshell protein family protein. |  |  |  |  | 0.09 | 0.12 |
| OsAffx.30127.1.S1_at | Os09g33650 expressed protein |  |  |  |  | 0.08 | 0.11 |
| Os.27765.1.S1_at | Os06g0714800 Protein of unknown function |  | 0.39 | 0.25 |  | 0.08 | 0.12 |
| Os.50858.1.S1_at | unknown | 0.26 |  |  |  | 0.08 | 0.07 |
| Os.54229.1.S1_at | Os04g0444300 Conserved hypothetical protein. | 3.04 |  |  |  | 0.08 | 0.12 |
| OsAffx.16746.1.S1_at | unknown |  |  |  |  | 0.07 | 0.07 |
| Os.10013.1.S1_at | Os10g0453900 Eggshell protein family protein. |  |  |  |  | 0.07 | 0.10 |
| Os.6764.1.S1_at | Os04g0635400 Conserved hypothetical protein |  |  |  | 0.25 | 0.07 | 0.04 |
| Os.15791.1.S1_at | Os04g0545700 Conserved hypothetical protein. | 0.33 |  |  |  | 0.07 | 0.02 |
| OsAffx.14006.1.S1_at | unknown |  |  |  |  | 0.07 | 0.10 |
| Os.31171.1.S1_at | Os01g0585200 Conserved hypothetical protein. |  |  |  |  | 0.06 | 0.06 |
| Os.9827.1.S1_at | Os01g0550800 Protein of unknown function | 33.87 |  |  |  | 0.06 | 0.02 |
| Os.18378.1.S1_s_at | Os10g0335000 Conserved hypothetical protein. |  |  |  |  | 0.06 | 0.02 |
| Os.42124.1.S1_at | Os01g0748300 Protein of unknown function |  |  |  |  | 0.06 | 0.04 |
| Os.11147.1.S1_at | unknown | 3.32 |  |  |  | 0.06 | 0.06 |
| Os.27908.1.A1_at | unknown |  |  |  |  | 0.06 | 0.18 |
| Os.57309.1.S1_at | Os04g0268700 Eggshell protein family protein. |  |  | 6.15 |  | 0.06 | 0.05 |
| Os.8510.1.S1_at | Os10g0333700 Hypothetical protein. |  |  |  |  | 0.05 | 0.02 |
| Os.46669.1.A1_at | Os10g0455300 Conserved hypothetical protein. |  |  |  |  | 0.05 | 0.03 |
| Os.54453.1.S1_at | Os08g0556300 Conserved hypothetical protein. |  |  |  |  | 0.05 | 0.03 |
| Os.6653.1.S1_at | unknown |  |  |  |  | 0.05 | 0.01 |
| Os.32298.1.S1_at | Os03g0187800 Protein of unknown function |  |  |  |  | 0.04 | 0.04 |
| Os.46829.1.S1_at | Os10g0454700 Eggshell protein family protein. |  |  |  |  | 0.04 | 0.04 |
| OsAffx.30199.1.S1_at | unknown |  |  |  |  | 0.04 | 0.02 |
| Os.50536.1.S1_at | Os10g0452300 Eggshell protein family protein. |  |  |  |  | 0.04 | 0.01 |
| OsAffx.28795.1.S1_at | unknown |  |  |  |  | 0.03 | 0.03 |
| Os.21894.1.S1_at | Os10g0455100 Eggshell protein family protein. |  |  |  |  | 0.03 | 0.04 |
| OsAffx.12386.1.S1_at | Os02g0587300 Hypothetical protein. |  |  |  |  | 0.03 | 0.02 |
| Os.54362.1.S1_at | unknown |  |  |  |  | 0.03 | 0.02 |
| OsAffx.19837.1.S1_at | unknown |  |  |  |  | 0.02 | 0.03 |
| OsAffx.11145.1.S1_s_at | Os01g0318400 Hypothetical protein. | 3.63 |  |  |  | 0.02 | 0.05 |
| Os.428.1.S1_at | Os03g0766600 Hypothetical protein. |  |  | 0.27 | 2.17 | 0.02 | 0.04 |
| OsAffx.16673.1.S1_s_at | Os07g0604700 B12D family protein. |  |  |  |  | 0.01 | 0.02 |
| Os.51451.1.S1_at | Os07g0604600 B12D family protein. |  |  |  |  | 0.00 | 0.00 |
| Os.49765.1.S1_at | Os04g0542200 Oligopeptide transporter |  | 0.32 | 0.24 |  | 0.16 | 0.12 |
| Os.54520.1.S1_at | Os04g0445000 Potassium channel SKOR |  |  |  |  | 0.14 | 0.11 |
| Os.12191.1.S1_at | Os03g0233900 Non-symbiotic hemoglobin 1 |  |  |  |  | 0.03 | 0.02 |
| Os.12163.1.S1_at | Os03g0226200 Non-symbiotic hemoglobin 2 |  |  |  |  | 0.01 | 0.01 |
| Os.6682.1.A1_s_at | Os03g0745200 Transferase family protein. | 0.48 | 3.12 | 8.16 |  | 0.18 | 0.16 |
| Os.29859.1.S1_at | Os07g0244200 Transferase family protein. |  |  |  |  | 0.15 | 0.09 |
| Os.56665.1.A1_at | Os10g0390800 ERF domain containing protein |  |  |  |  | 0.15 | 0.16 |
| Os.8031.1.S1_at | Os01g0313300 Transcription factor EREBP1. |  |  |  |  | 0.12 | 0.03 |
| Os.11120.1.S1_at | Os03g0182800 Ethylene responsive element binding factor3 |  |  |  |  | 0.07 | 0.06 |
| Os.4893.1.S1_at | Os05g0361700 Ethylene responsive element binding factor3 | 7.72 |  |  |  | 0.04 | 0.07 |
| Os.6539.1.S1_at | Os04g0529100 AP2 domain containing protein RAP2.4 |  |  |  |  | 0.19 | 0.18 |
| OsAffx.24232.2.S1_s_at | Os02g13710 transcriptional factor TINY, putative, expressed |  |  |  | 0.34 | 0.18 | 0.15 |
| Os.57381.1.S1_at | Os03g0341000 AP2 domain containing protein RAP2.2 |  |  |  |  | 0.03 | 0.01 |
| Os.8920.1.S1_at | Os07g0674800 AP2 domain containing protein RAP2.2 |  |  |  |  | 0.01 | 0.03 |
| Os.802.1.S1_at | Os01g0104200 NAC-domain protein 5-8. |  |  | 9.38 |  | 0.18 | 0.17 |
| Os.27420.2.A1_s_at | Os05g0261700 Leucine-rich repeat |  |  |  |  | 0.17 | 0.10 |
| Os.31303.1.S1_at | Os03g0188400 Basic helix-loop-helix dimerisation region |  |  |  |  | 0.17 | 0.13 |
| Os.52845.1.S1_at | Os03g0171300 DNA-binding protein-like. |  |  |  |  | 0.17 | 0.13 |
| Os.51307.1.S1_at | Os11g0702400 Zn-finger, C2H2 type domain containing protein. |  |  |  |  | 0.14 | 0.09 |
| Os.5860.1.S1_at | Os03g0135700 Transcriptional activator Rb homolog (Fragment). |  |  |  |  | 0.13 | 0.11 |
| OsAffx.12674.1.S1_at | Os02g0813100 Cyclin-like F-box domain containing protein. |  |  | 6.29 |  | 0.11 | 0.12 |
| Os.36672.1.S1_at | Os06g0107800 RAV-like protein. |  |  |  |  | 0.10 | 0.08 |
| Os.54467.1.S1_at | Os03g0335200 WRKY DNA binding protein. |  |  |  |  | 0.09 | 0.08 |
| Os.36651.1.S1_at | Os04g0515900 NAM / CUC2-like protein. |  |  |  |  | 0.09 | 0.03 |
| Os.7694.1.S1_at | Os04g0489600 Basic helix-loop-helix dimerisation region bHLH domain containing protein. |  |  | 2.78 | 2.08 | 0.07 | 0.06 |
| Os.46849.1.S1_at | Os10g0391400 ZIM domain containing protein. |  |  | 6.77 |  | 0.07 | 0.07 |
| Os.24417.3.S1_at | Os08g0396700 DC1 domain containing protein. |  | 2.19 |  |  | 0.06 | 0.10 |
| Os.22577.1.S1_x_at | Os01g0511000 LOB domain protein 40. |  |  |  |  | 0.06 | 0.17 |
| Os.53575.1.S1_s_at | Os03g0338400 Basic helix-loop-helix dimerisation region bHLH domain containing protein. |  |  |  |  | 0.03 | 0.01 |
| Os.51658.1.S1_at | Os06g0707300 Cyclin-like F-box domain containing protein. |  |  |  |  | 0.03 | 0.08 |
| Os.37718.1.S1_at | Os10g0409400 BURP domain containing protein. | 8.79 |  | 2.78 |  | 0.04 | 0.03 |
| Os.21893.1.S1_x_at | Os04g0688200 Anionic peroxidase precursor. |  |  |  |  | 0.20 | 0.17 |
| Os.48028.1.S1_x_at | Os10g0498100 Epoxide hydrolase family protein. |  |  |  |  | 0.20 | 0.14 |
| Os.32630.1.S1_at | Os10g0464000 Hypersensitive-induced response protein. |  |  | 5.16 |  | 0.18 | 0.10 |
| Os.53348.2.S1_at | Os11g0270500 Disease resistance protein family protein. |  |  |  |  | 0.16 | 0.20 |
| Os.11024.1.S1_at | Os10g0161400 Disease resistance protein family protein. |  |  |  |  | 0.15 | 0.12 |
| OsAffx.2166.1.S1_at | Os01g36560 nodulin-like protein, putative, expressed |  |  |  |  | 0.13 | 0.04 |
| Os.18707.1.S1_at | Os11g0134900 Major facilitator superfamily antiporter. |  |  |  |  | 0.13 | 0.05 |
| Os.46616.1.S1_at | Os10g0569600 RIR1b protein precursor. |  |  |  |  | 0.13 | 0.07 |
| Os.32890.1.S1_at | Os11g0702100 Class III chitinase homologue |  |  | 65.45 | 5.74 | 0.12 | 0.11 |
| Os.27755.1.S1_at | Os07g0539900 Beta-1,3-glucanase-like protein. |  |  |  |  | 0.11 | 0.04 |
| Os.11344.1.S1_s_at | Os05g0554000 Multi antimicrobial extrusion protein MatE family protein. |  |  |  |  | 0.11 | 0.13 |
| Os.54195.1.S1_at | Os11g0151500 Major facilitator superfamily protein. |  |  |  |  | 0.10 | 0.13 |
| Os.40417.1.A1_at | Os07g0251200 Harpin-induced 1 domain containing protein. |  |  |  |  | 0.08 | 0.09 |
| Os.50837.1.S1_at | Os11g0701500 Class III chitinase homologue |  |  | 2.67 |  | 0.05 | 0.08 |
| Os.55340.1.S1_at | Os04g0666800 Plant disease resistance response protein | 0.24 |  |  |  | 0.05 | 0.04 |
| Os.47802.1.A1_at | Os12g36840 pathogenesis-related protein 10, putative, expressed |  |  |  |  | 0.02 | 0.01 |
| Os.6645.1.S1_at | Os07g25050 thionin-like peptide, putative, expressed |  |  |  |  | 0.02 | 0.01 |
| Os.56924.1.S1_at | Os05g0493800 MtN21 nodulin protein-like. |  |  |  |  | 0.16 | 0.16 |
| Os.47601.1.A1_at | Os12g0228700 Beta-glucosidase aggregating factor. |  |  |  |  | 0.14 | 0.15 |
| Os.7108.1.S1_at | Os02g0582900 Conotoxin family protein. |  |  |  |  | 0.06 | 0.01 |
| Os.51641.1.S1_at | Os07g0127600 Allergen V5/Tpx-1 related family protein. |  |  |  |  | 0.05 | 0.01 |
| Os.15706.1.S1_at | Os01g0666000 Lipid phosphate phosphatase 2 |  |  |  |  | 0.03 | 0.02 |
| Os.4159.1.S1_at | Os01g0713200 Beta-1,3-glucanase precursor. |  |  | 0.35 |  | 0.02 | 0.02 |
| OsAffx.2917.1.S1_at | Os02g0609200 ATOZI1 protein |  |  |  |  | 0.01 | 0.01 |
| Os.51835.1.S1_a_at | Os05g0560900 Gibberellin 2-beta-dioxygenase | 6.73 | 32.39 | 8.41 | 9.84 | 0.03 | 0.04 |
| Os.17474.1.A1_at | Os02g11790 protein kinase Kelch repeat |  |  | 2.13 |  | 0.03 | 0.08 |
| Os.18598.1.S1_at | Os03g0699700 Lipoxygenase (EC 1.13.11.12) |  |  |  |  | 0.19 | 0.17 |
| Os.53604.1.S1_at | Os03g0738600 Lipoxygenase L-2 (EC 1.13.11.12). |  |  |  |  | 0.11 | 0.14 |
| Os.54257.1.S1_at | Os11g0555600 Protein kinase domain containing protein. |  |  |  |  | 0.20 | 0.12 |
| Os.48695.1.S1_s_at | Os07g0538400 Receptor-like protein kinase 4. |  |  |  |  | 0.20 | 0.07 |
| OsAffx.26118.1.S1_at | Os04g0303500 Protein kinase domain containing protein. |  |  |  |  | 0.18 | 0.12 |
| Os.51241.1.S1_at | Os11g0557000 Phytosulfokine family protein. | 0.21 | 0.40 |  |  | 0.18 | 0.09 |
| Os.27431.1.A1_at | Os07g0537500 Protein kinase domain containing protein. |  |  |  |  | 0.17 | 0.09 |
| OsAffx.5047.1.S1_at | Os06g38590 receptor-like protein kinase precursor |  |  |  |  | 0.16 | 0.15 |
| Os.33145.1.S1_at | Os07g0550900 Receptor-like protein kinase 6. |  |  |  |  | 0.16 | 0.14 |
| Os.18692.1.S1_at | Os02g0648100 Protein kinase domain containing protein. |  |  |  |  | 0.16 | 0.18 |
| Os.46749.1.S1_x_at | Os09g0453300 Annexin family protein. | 0.41 |  |  |  | 0.16 | 0.20 |
| Os.9585.1.S1_s_at | Os12g0603800 Calmodulin NtCaM13. |  |  |  |  | 0.13 | 0.04 |
| Os.12664.1.S1_at | Os07g0542600 Protein kinase family protein. |  |  |  |  | 0.09 | 0.11 |
| Os.27185.1.S1_x_at | Os07g0541900 Protein kinase domain containing protein. |  |  |  |  | 0.09 | 0.04 |
| OsAffx.985.7.S1_x_at | Os01g0810500 Protein kinase domain containing protein. |  |  |  |  | 0.08 | 0.11 |
| Os.53676.1.S1_at | Os08g0372900 Annexin A7 (Annexin VII) |  |  |  |  | 0.06 | 0.10 |
| OsAffx.985.1.S1_x_at | Os01g0810900 Protein kinase domain containing protein. |  |  |  |  | 0.04 | 0.06 |
| Os.18744.1.S1_at | Os01g0810800 Receptor protein kinase-like protein. |  |  |  |  | 0.03 | 0.04 |
| Os.32686.1.S1_at | Os01g0871600 TGF-beta receptor, type I/II extracellular region family protein. |  |  |  |  | 0.03 | 0.02 |
| Os.27934.2.S1_x_at | Os03g0245700 Senescence-associated protein 15. | 0.25 | 2.53 |  |  | 0.14 | 0.09 |
| OsAffx.20724.2.S1_s_at | Os10g0158100 Senescence-associated protein 15. |  |  | 6.89 | 5.07 | 0.10 | 0.05 |
| Os.35425.1.S1_at | Os01g0836600 ABC transporter related domain containing protein. |  |  |  |  | 0.13 | 0.05 |
| Os.6125.1.S1_at | Os03g0405500 PDI-like protein. |  | 2.07 |  |  | 0.18 | 0.16 |
| Os.11554.1.S1_at | Os06g0521900 Haem peroxidase |  |  |  |  | 0.16 | 0.13 |
| Os.2965.1.S1_at | Os03g0368000 Peroxidase 1. |  |  |  |  | 0.11 | 0.02 |
| Os.5905.1.S1_at | Os06g0521500 Haem peroxidase |  |  |  |  | 0.10 | 0.14 |
| Os.2961.1.S1_at | Os03g0369000 Peroxidase. |  |  |  |  | 0.08 | 0.04 |
| Os.19737.1.S1_at | Os03g0368300 Peroxidase. |  |  |  |  | 0.08 | 0.04 |
| Os.47413.1.S1_x_at | Os06g0522300 Haem peroxidase |  |  |  |  | 0.04 | 0.04 |
| Os.47413.1.S1_at | Os06g0522300 Haem peroxidase |  |  |  |  | 0.04 | 0.03 |
| Os.8815.1.S1_at | Os11g0210500 Alcohol dehydrogenase 2. |  |  |  |  | 0.07 | 0.06 |
| Os.5185.1.S1_at | Os07g0682000 Heavy metal transport |  |  |  |  | 0.19 | 0.12 |
| Os.56899.1.S1_at | Os07g0639400 Peroxidase 1. |  |  |  |  | 0.17 | 0.15 |
| Os.11556.1.S1_at | Os07g0677300 Peroxidase. |  |  |  |  | 0.15 | 0.04 |
| Os.22086.1.S1_at | Os07g0677200 Peroxidase. |  |  |  |  | 0.06 | 0.03 |
| OsAffx.27946.1.S1_at | Os06g0542300 Heavy metal transport |  |  |  |  | 0.20 | 0.16 |
| Os.11309.1.S1_x_at | Os05g0135400 Plant peroxidase family protein. |  |  |  |  | 0.16 | 0.09 |
| Os.23290.1.S1_at | Os10g0558900 2OG-Fe(II) oxygenase domain containing protein. |  | 0.33 | 0.31 |  | 0.16 | 0.20 |
| Os.15894.1.A1_a_at | Os01g0962700 Peroxidase 12 precursor | 2.43 |  |  |  | 0.16 | 0.12 |
| Os.50961.1.S1_at | Os03g0803500 2OG-Fe(II) oxygenase domain containing protein. |  |  | 2.20 |  | 0.14 | 0.07 |
| OsAffx.4250.1.S1_s_at | Os05g0134800 Haem peroxidase |  |  |  |  | 0.14 | 0.04 |
| Os.56907.1.S1_at | Os09g0447500 Cytochrome P450 family protein. |  |  |  |  | 0.10 | 0.08 |
| Os.11333.1.S1_s_at | Os02g0585100 Heavy metal transport |  |  |  |  | 0.09 | 0.09 |
| Os.50554.1.S1_at | Os07g0194500 2OG-Fe(II) oxygenase domain containing protein. |  | 2.02 |  |  | 0.08 | 0.05 |
| OsAffx.7566.1.S1_at | Os12g0225900 Allyl alcohol dehydrogenase. | 0.35 |  |  |  | 0.07 | 0.07 |
| Os.53667.1.S1_at | Os06g0546500 Peroxidase. |  |  |  |  | 0.04 | 0.03 |
| Os.51432.1.S1_at | Os02g0791400 Cytochrome oxidase c, subunit VIb family protein. |  |  |  |  | 0.01 | 0.01 |
| Os.47977.1.A1_at | Os06g0592400 Cytosolic aldehyde dehydrogenase RF2C. |  |  | 8.36 |  | 0.12 | 0.14 |
| Os.7579.1.S1_at | Os11g0499600 Short-chain dehydrogenase | 0.36 |  |  |  | 0.19 | 0.20 |
| Os.12839.1.S1_at | Os04g0483500 B-keto acyl reductase. | 0.38 |  | 0.41 | 0.31 | 0.16 | 0.09 |
| Os.9724.1.S1_at | Os03g0220100 Very-long-chain fatty acid condensing enzyme CUT1 | 0.45 |  | 0.40 | 0.48 | 0.11 | 0.06 |
| Os.5037.1.S1_s_at | Os12g0260500 Short-chain dehydrogenase |  |  |  |  | 0.10 | 0.04 |
| Os.4863.1.S1_at | Os01g0631200 Uroporphyrinogen III methyltransferase. |  |  |  | 0.50 | 0.16 | 0.17 |
| OsAffx.13256.1.S1_at | Os03g0595600 Glutathione S-transferase GST 29 (EC 2.5.1.18). |  |  |  |  | 0.12 | 0.12 |
| Os.5028.1.S1_at | Os02g0720600 Peptidase A1, pepsin family protein. |  |  | 0.33 | 0.33 | 0.19 | 0.10 |
| Os.24061.1.A1_at | Os02g0721100 Ubiquitin-conjugating enzyme |  |  |  |  | 0.16 | 0.19 |
| OsAffx.23634.2.S1_x_at | Os01g0608300 Peptidase A1, pepsin family protein. |  |  |  |  | 0.15 | 0.11 |
| Os.49973.1.S1_at | Os03g0317300 Peptidase A1, pepsin family protein. |  |  |  |  | 0.12 | 0.11 |
| Os.57406.1.S1_at | Os05g0280500 Phospholipid/glycerol acyltransferase family protein. | 0.48 |  | 3.99 |  | 0.11 | 0.05 |
| OsAffx.16561.1.S1_at | Os07g0532800 Peptidase A1, pepsin family protein. |  |  |  |  | 0.08 | 0.04 |
| Os.54672.1.S1_at | Os03g0318400 Peptidase A1, pepsin family protein |  |  |  |  | 0.07 | 0.04 |
| Os.10765.1.S1_at | Os11g0182200 Transferase family protein. | 4.04 |  |  |  | 0.06 | 0.05 |
| Os.48328.1.S1_at | Os01g0347600 Ervatamin B (EC 3.4.22.-) (ERV-B). |  |  |  |  | 0.05 | 0.02 |
| Os.7989.1.S1_at | Os02g20360 tyrosine aminotransferase, putative, expressed |  |  |  |  | 0.05 | 0.06 |
| OsAffx.30196.1.S1_at | Os09g0564200 Peptidase C1A, papain family protein. |  |  |  |  | 0.04 | 0.03 |
| Os.8360.1.S1_at | Os01g0347500 Peptidase C1A, papain family protein. |  |  |  |  | 0.03 | 0.02 |
| Os.20153.1.S1_x_at | Os01g0185500 RmlC-like cupin family protein. |  |  |  |  | 0.02 | 0.02 |
| Os.5086.1.S1_at | Os03g0150800 High affinity phosphate transporter 2 |  |  |  |  | 0.11 | 0.07 |
| Os.11391.1.S1_at | Os10g0116800 Purple acid phosphatase. |  |  |  |  | 0.02 | 0.04 |
| Os.26483.1.S1_at | Os04g0652700 Nuclease I. | 0.43 | 12.22 | 15.74 | 2.65 | 0.17 | 0.13 |
| Os.6651.1.S1_at | Os07g0630400 Ribonuclease T2 family protein. |  |  |  |  | 0.14 | 0.05 |
| OsAffx.20014.1.S1_at | Os12g38770 nucleotide pyrophosphatase/phosphodiesterase |  |  |  |  | 0.11 | 0.08 |
| Os.39043.1.S1_s_at | Os10g0580400 Urea active transporter-like protein. |  |  |  | 2.18 | 0.08 | 0.14 |
| Os.49093.1.S1_a_at | Os02g0112600 Membrane transporter |  |  |  |  | 0.07 | 0.08 |
| Os.55301.1.S1_x_at | Os09g0471000 RmlC-like cupin family protein. |  |  |  |  | 0.03 | 0.03 |
| OsAffx.12433.1.S1_at | Os02g0620500 High affinity ammonium transporter. |  |  |  |  | 0.02 | 0.03 |
| Os.49093.1.S1_at | Os02g0112100 BCH2. |  |  |  |  | 0.02 | 0.02 |
| Os.7269.1.S1_s_at | Os01g0636400 Alpha/beta hydrolase family protein. |  |  |  |  | 0.19 | 0.17 |
| OsAffx.28337.1.S1_at | Os07g0174900 Plant lipid transfer protein |  |  |  |  | 0.06 | 0.03 |
| Os.55417.1.S1_at | Os08g0253800 Glycosyl transferase |  |  |  |  | 0.15 | 0.20 |
| Os.50392.1.S1_at | Os07g0421300 Glycoside hydrolase, family 31 protein. |  |  |  |  | 0.15 | 0.07 |
| Os.6776.1.S1_at | Os07g0539100 Glycoside hydrolase, family 17 protein. |  |  |  |  | 0.13 | 0.12 |
| Os.23174.1.S1_s_at | Os08g0240500 Glycoside hydrolase, family 16 protein. |  |  |  |  | 0.09 | 0.06 |
| Os.56341.1.S1_at | Os04g0480900 Glycoside hydrolase, family 5 protein. | 2.31 |  |  |  | 0.04 | 0.03 |
| Os.7583.1.S1_x_at | Os04g0529700 Alpha 1,4-glycosyltransferase conserved region family protein |  |  |  |  | 0.20 | 0.09 |
| Os.51664.1.S1_at | Os06g0220500 UDP-glucuronosyl/UDP-glucosyltransferase family protein. |  |  |  |  | 0.20 | 0.11 |
| Os.54760.1.S1_at | Os05g0574100 Lipase, class 3 family protein. |  |  |  |  | 0.19 | 0.04 |
| Os.53148.1.S1_at | Os03g0737300 Magnesium-dependent phosphatase-1 family protein. |  |  |  |  | 0.15 | 0.14 |
| Os.9333.1.A1_s_at | Os06g0229400 Lipolytic enzyme, G-D-S-L family protein. |  |  |  |  | 0.15 | 0.09 |
| Os.10215.1.S1_at | Os02g0105400 L-lactate dehydrogenase A | 0.22 |  |  | 0.39 | 0.12 | 0.08 |
| Os.12528.1.S1_x_at | Os03g0432100 Pyruvate, phosphate dikinase |  |  | 2.79 | 2.62 | 0.09 | 0.05 |
| Os.37622.1.S1_at | Os01g0895200 Family 9 carbohydrate-binding module/cellobiose dehydrogenase |  |  |  |  | 0.07 | 0.04 |
| Os.6838.1.S1_at | Os10g0552600 Plant lipid transfer/seed storage/trypsin-alpha amylase inhibitor domain containing protein. |  |  |  |  | 0.05 | 0.01 |
| Os.9534.1.S1_at | Os01g0880800 Acyl-[acyl-carrier-protein] desaturase, chloroplast precursor |  |  |  |  | 0.02 | 0.02 |
| Os.4683.1.S1_at | Os05g0469600 Pyruvate decarboxylase isozyme 1 |  |  |  |  | 0.02 | 0.03 |
| Os.4683.3.S1_at | Os05g0469800 Pyruvate decarboxylase (EC 4.1.1.1) (Fragment). |  |  |  |  | 0.02 | 0.03 |
| Os.31913.1.S1_at | Os06g0104900 L-lactate dehydrogenase B |  |  |  |  | 0.01 | 0.01 |
| Os.27638.1.S1_at | Os05g0555600 NADH dependent Glutamate Synthase precursor | 2.76 |  |  |  | 0.01 | 0.01 |
| Os.8805.1.S1_at | Os10g0204400 Phosphoenolpyruvate carboxykinase. |  |  |  |  | 0.01 | 0.01 |
| Os.10972.1.S1_at | Os01g0878700 Amino acid/polyamine transporter II family protein. |  |  | 2.33 | 2.30 | 0.14 | 0.11 |
| OsAffx.7934.1.S1_at | Os10g39390 aspartic proteinase nepenthesin-2 precursor |  |  |  |  | 0.07 | 0.06 |
| OsAffx.30198.1.S1_at | Os09g39110 vignain precursor, putative, expressed |  |  |  |  | 0.02 | 0.01 |
| OsAffx.24544.1.S1_at | Os02g0529800 Cytochrome P450 family protein. |  |  |  |  | 0.14 | 0.19 |
| Os.54318.1.S1_at | Os11g0138300 E-class P450, group I family protein. |  |  |  |  | 0.11 | 0.11 |
| Os.53289.1.S1_at | Os12g0197100 Phosphoribosylamine--glycine ligase |  |  |  |  | 0.03 | 0.04 |
| Os.37330.1.S1_at | Os01g0802700 Auxin Efflux Carrier family protein. |  |  |  |  | 0.07 | 0.02 |
| Os.51847.1.S1_x_at | Os03g0759700 ER33 protein (Fragment). |  |  |  |  | 0.14 | 0.11 |
| Os.56016.1.S1_at | Os03g0853200 CD9/CD37/CD63 antigen family protein. |  |  |  |  | 0.19 | 0.13 |
| Os.46832.1.S1_at | Os10g0418100 Calcium-transporting ATPase 8 |  | 2.54 | 5.99 |  | 0.16 | 0.14 |
| OsAffx.28364.1.S1_at | Os07g0193000 HIPL1 protein precursor. |  |  |  |  | 0.13 | 0.10 |
| Os.28773.1.S1_at | Os01g0248900 Expansin EXPA5. |  |  |  |  | 0.20 | 0.12 |
| OsAffx.14448.1.S1_at | Os04g0664900 Cell wall invertase (EC 3.2.1.26). |  |  |  |  | 0.19 | 0.13 |
| Os.410.1.S1_at | Os05g0276500 Expansin Os-EXPA3. |  |  |  |  | 0.17 | 0.14 |
| Os.51562.1.S1_at | Os04g0445300 Plant invertase/pectin methylesterase inhibitor domain containing protein. |  |  |  |  | 0.16 | 0.10 |
| Os.26933.1.S1_x_at | Os10g0490100 Barwin-related endoglucanase domain containing protein. |  |  |  |  | 0.13 | 0.10 |
| Os.38638.3.S1_x_at | Os06g0142400 Early nodulin 93 ENOD93 protein family protein |  |  |  |  | 0.13 | 0.15 |
| OsAffx.27459.2.S1_s_at | Os06g0142300 Early nodulin 93 ENOD93 protein family protein. |  |  |  |  | 0.10 | 0.14 |
| Os.38638.1.S1_at | Os06g0142200 Early nodulin 93 ENOD93 protein family protein. | 8.18 |  |  |  | 0.03 | 0.04 |
| Os.27505.1.S1_at | Os01g0788400 Pectinesterase |  | 3.84 | 10.07 | 2.66 | 0.15 | 0.10 |
| Os.5588.1.S1_s_at | Os03g0196000 Sulfate transporter ST1. | 0.38 |  |  |  | 0.06 | 0.03 |
| Os.46777.1.S1_at | Os04g0365100 Wall-associated kinase-like protein. |  |  |  |  | 0.17 | 0.11 |
| Os.56033.1.S1_at | Os04g0481800 Membrane bound O-acyl transferase |  |  |  |  | 0.07 | 0.14 |
| Os.10930.1.S1_at | Os02g0626600 Phenylalanine ammonia-lyase. |  |  |  |  | 0.12 | 0.05 |
| Os.54653.1.S1_at | Os06g0199000 Glycine-rich cell wall structural protein 2 precursor. |  |  |  |  | 0.11 | 0.01 |
| OsAffx.19698.1.S1_at | Os12g0259800 Multicopper oxidase |  |  |  |  | 0.13 | 0.11 |
| Os.45894.1.S1_x_at | Os01g0283600 Cinnamoyl-CoA reductase (EC 1.2.1.44). |  |  | 0.29 |  | 0.09 | 0.08 |
| Os.11417.1.S1_at | Os02g0467600 Cinnamate 4-hydroxylase CYP73. |  |  | 3.33 |  | 0.05 | 0.02 |
| Os.31788.1.S1_at | Os01g0127000 Multicopper oxidase, type 1 domain containing protein. |  |  |  |  | 0.04 | 0.04 |
| Os.49631.2.S1_x_at | Os07g0517100 Seed maturation protein PM31. | 0.28 | 0.21 | 0.26 | 0.23 | 0.16 | 0.16 |
| Os.18395.1.S1_s_at | Os06g0266800 GAST1 protein precursor. | 0.19 |  |  |  | 0.10 | 0.09 |
