# Supplementary material for: Genome-wide temporal-spatial gene expression profiling of drought responsiveness in rice
Source: BMC Genomics. 2011 Mar 16;12:149. doi: 10.1186/1471-2164-12-149 (PMC3070656; doi:10.1186/1471-2164-12-149)
Supplement: Additional file 14 — Panicle-specific up-regulated genes under drought stress. Excel file containing all specific up-regulated genes by drought in panicle [file 1471-2164-12-149-S14.DOC]

**Additional file 14. Panicle specific up-regulated genes under drought stress.**

| **Gene ID** | **Annotation** | **BP** | **BL** | **PL** | **TL** | **PR** | **TR** |
| --- | --- | --- | --- | --- | --- | --- | --- |
| Os.37927.1.S1_at | Os01g0919200 Cell division protein FtsZ family protein. | 29.96 |  |  |  |  |  |
| Os.22396.1.S1_at | Os05g0283200 Plant invertase/pectin methylesterase inhibitor domain containing protein. | 6.32 |  |  |  |  |  |
| Os.54919.1.S1_at | Os05g0543000 Plant invertase/pectin methylesterase inhibitor domain containing protein. | 50.96 |  |  |  |  |  |
| Os.4957.1.S1_at | Os07g0247000 Plant invertase/pectin methylesterase inhibitor domain containing protein. | 17.41 |  |  |  |  |  |
| Os.38638.1.S1_at | Os06g0142200 Early nodulin 93 ENOD93 protein family protein. | 8.18 |  |  |  | 0.03 | 0.04 |
| Os.11812.1.S1_at | Os02g0719600 SAM dependent carboxyl methyltransferase family protein. | 7.22 |  |  | 0.26 |  |  |
| OsAffx.29770.1.S1_at | Os09g0262000 Cinnamoyl-CoA reductase (involved in lignin specific biosynthetic pathway). | 6.80 |  |  | 0.17 |  |  |
| Os.4956.1.S1_at | Os12g0420100 Beta-glucosidase. | 16.17 |  |  |  |  |  |
| Os.2402.1.S1_at | Os03g0106500 Beta-expansin precursor (Beta-expansin 1) | 14.15 |  |  |  |  |  |
| Os.12697.1.S1_at | Os10g0548600 Beta-expansin precursor. | 26.21 |  |  |  |  |  |
| Os.11335.3.S1_x_at | Os01g0720700 Serine acetyltransferase. | 30.75 | 4.42 | 3.10 |  | 0.44 | 0.33 |
| Os.1440.2.S1_x_at | Os01g0159400 Acyl-CoA dehydrogenase family protein. | 5.17 |  | 2.90 |  |  | 0.42 |
| Os.33696.1.S1_at | Os01g0695300 Farnesyl pyrophosphate synthetase (FPP synthetase) (FPS) | 5.24 | 3.59 |  | 2.35 | 4.14 | 3.63 |
| Os.26829.2.S1_s_at | Os05g0180000 UDP-glucuronosyl/UDP-glucosyltransferase family protein. | 5.20 |  | 3.44 | 0.19 |  |  |
| Os.4518.1.S1_a_at | Os06g0115300 Acyl-CoA-binding protein. | 5.42 |  |  |  | 2.63 | 2.65 |
| Os.15696.1.S1_a_at | Os02g0644000 C13 endopeptidase NP1 (Fragment). | 5.61 | 3.62 | 4.74 | 4.02 | 3.53 | 3.19 |
| Os.7935.1.S1_at | Os07g0663800 Short-chain dehydrogenase/reductase SDR family protein. | 42.29 |  |  | 0.32 | 0.24 | 0.36 |
| Os.12777.1.S1_a_at | Os10g0323900 Profilin A.; Os10g0323600 Profilin A. | 10.70 |  |  |  |  |  |
| Os.25407.1.S1_at | Os04g0317500 Pollen allergen Lol p2 family protein. | 14.77 |  |  |  |  |  |
| Os.9665.1.S1_at | Os04g0317800 Pollen allergen Lol p2 family protein. | 42.68 |  |  |  |  |  |
| Os.53412.1.S1_at | Os06g0556600 Pollen Ole e 1 allergen and extensin domain containing protein. | 24.78 |  |  |  |  |  |
| Os.7428.1.S1_at | Os06g0655200 Pollen allergen Lol p2 family protein. | 24.75 |  |  |  |  |  |
| Os.12324.1.S1_at | Os08g0560700 Polcalcin Phl p 7 (Calcium-binding pollen allergen Phl p 7) (P7). | 6.51 |  |  |  |  |  |
| Os.8140.1.S1_at | Os10g0351700 Tapetum specific protein (Tapetum-specific endoxylanase). | 8.73 |  |  |  |  |  |
| Os.12300.1.S1_at | Os01g0816700 L-ascorbate oxidase homolog precursor (EC 1.10.3.3) (Ascorbase). | 19.02 |  |  |  |  |  |
| Os.17180.1.S1_at | Os08g0547300 E-class P450, group I family protein. | 9.64 | 0.07 | 0.05 | 0.31 | 0.36 |  |
| Os.9103.1.S1_at | Os09g0272000 Heavy metal transport/detoxification protein domain containing protein. | 10.06 | 2.99 |  |  |  |  |
| Os.55402.1.S1_at | Os11g0655900 Glutaredoxin domain containing protein. | 7.84 | 0.40 |  |  |  |  |
| Os.2371.1.S1_at | Os12g0448900 Animal haem peroxidase family protein. | 18.13 | 4.70 |  |  |  |  |
| Os.11550.1.S1_at | Os03g0152300 Haem peroxidase, plant/fungal/bacterial family protein. | 53.02 |  |  |  |  |  |
| Os.313.1.S1_a_at | Os02g0730000 Mitochondrial aldehyde dehydrogenase ALDH2a. | 5.32 |  |  |  | 0.28 | 0.15 |
| Os.9279.1.S1_at | Os12g0570700 Metallothionein-like protein. | 5.28 |  | 2.69 |  |  |  |
| OsAffx.27444.1.S1_at | Os06g0127500 MEG5. | 5.45 | 2.83 | 2.19 |  | 2.32 | 2.51 |
| Os.25589.3.S1_x_at | Os11g0444900 Octicosapeptide/Phox/Bem1p domain containing protein. | 11.85 | 2.42 |  |  | 2.24 | 4.84 |
| Os.10636.2.A1_x_at | Os06g0159600 U box domain containing protein. | 6.56 | 2.45 | 2.99 | 2.28 |  |  |
| Os.47654.1.S1_at | Os05g0562200 Drought induced 19 family protein. | 5.11 |  |  | 2.20 |  |  |
| Os.37718.1.S1_at | Os10g0409400 BURP domain containing protein (drought related). | 8.79 |  | 2.78 |  | 0.04 | 0.03 |
| Os.3729.1.S1_at | Os01g0132000 Bowman-Birk type wound induced proteinase inhibitor WIP1 precursor. | 22.60 | 3.16 | 2.27 |  | 2.03 |  |
| OsAffx.3569.1.S1_at | Os03g0718800 Physical impedance induced protein. | 5.38 |  |  |  | 4.69 | 4.50 |
| Os.27383.1.S1_at | Os04g0189400 Gamma Purothionin family protein. | 7.44 |  |  |  |  |  |
| Os.10166.1.S1_at | Os04g0494100 Endochitinase A precursor (EC 3.2.1.14) (Seed chitinase A). | 6.18 |  |  |  |  |  |
| Os.22058.1.S1_at | Os10g0542900 Chitinase (EC 3.2.1.14) (Fragment). | 7.64 |  |  |  |  | 0.39 |
| Os.28044.1.A1_at | Os04g0493600 Chitin-binding, type 1 domain containing protein. | 5.42 |  |  |  |  |  |
| Os.49294.1.S1_at | Os09g0509700 Hd1. | 6.91 |  |  |  | 2.47 | 3.39 |
| Os.13615.4.S1_x_at | Os01g0661500 Mov34/MPN/PAD-1 family protein | 6.58 | 2.91 | 3.69 |  |  | 2.55 |
| Os.22665.1.S1_at | Os03g0109400 Homeobox domain containing protein. | 11.70 |  |  |  |  |  |
| Os.6271.1.S1_at | Os07g0581700 Homeodomain leucine zipper protein CPHB-4. | 10.78 |  |  |  |  |  |
| Os.11094.1.A1_at | Os04g0546800 Ethylene responsive element binding factor 5 (AtERF5). | 12.01 |  | 2.39 |  |  |  |
| Os.4893.1.S1_at | Os05g0361700 Ethylene responsive element binding factor3 (OsERF3). | 7.72 |  |  |  | 0.04 | 0.07 |
| Os.12691.3.S1_x_at | Os03g0324300 EIL3.; Os03g0324200 EIL3. | 7.98 |  |  |  |  |  |
| Os.7362.1.S1_at | Os07g0684800 GRAB2 protein. | 7.40 | 3.95 | 4.02 | 2.10 |  | 0.48 |
| Os.11773.1.S1_at | Os05g0343400 WRKY transcription factor 53 (Transcription factor WRKY12). | 5.69 |  |  |  |  |  |
| Os.11080.1.S1_at | Os12g0542000 RALF. | 9.63 |  |  |  |  |  |
| Os.2971.1.S1_at | Os03g0369100 Plant lipid transfer | 5.27 |  |  |  |  |  |
| Os.27618.1.S1_at | Os05g0533900 ClpX, ATPase regulatory subunit family protein. | 6.51 | 2.64 |  | 2.95 | 3.06 | 2.03 |
| Os.19230.2.S1_at | Os06g0602700 Mitochondrial brown fat uncoupling protein family protein. | 5.56 |  |  |  | 2.67 | 2.95 |
| Os.52968.1.S1_at | Os11g0297300 Beta-D-xylosidase. | 5.58 |  |  |  |  |  |
| Os.18225.1.S1_at | Os11g0657400 Atrophin family protein. | 22.76 |  |  |  |  |  |
| Os.18664.2.S1_x_at | Os01g0946500 Glucan endo-1,3-beta-glucosidase GV | 6.84 |  |  |  | 4.56 | 3.84 |
| OsAffx.23673.1.S1_at | LOC_Os01g45670 expressed protein | 5.89 | 2.33 |  | 2.80 | 2.06 | 2.72 |
| OsAffx.20370.1.S1_at | LOC_Os10g25990 retrotransposon protein, putative, unclassified, expressed | 6.05 |  |  |  |  |  |
| Os.12321.1.S1_at | Os05g0597700 Hypothetical protein. | 21.62 |  |  |  |  |  |
| Os.10736.1.S1_at | Os06g0147100 Conserved hypothetical protein. | 5.23 |  | 3.11 |  | 2.32 | 2.11 |
| Os.52964.1.S1_at | Os06g0242700 Conserved hypothetical protein. | 6.25 |  | 2.93 |  |  |  |
| OsAffx.15474.1.S1_s_at | Os06g0284900 Hypothetical protein. | 12.15 |  |  |  |  |  |
| Os.56057.1.S1_at | Os06g0609400 Hypothetical protein. | 5.63 |  |  |  |  |  |
| OsAffx.10198.2.A1_at | Os07g0237200 Conserved hypothetical protein. | 19.94 |  |  |  |  |  |
| Os.18406.1.S1_at | Os07g0591700 Conserved hypothetical protein. | 5.66 | 0.10 |  |  |  | 0.38 |
| Os.8166.1.S1_at | Os08g0222100 Conserved hypothetical protein. | 30.27 |  |  |  |  |  |
| Os.12779.1.S1_at | Os09g0477100 Hypothetical protein. | 7.78 |  |  |  |  |  |
| Os.50366.1.S1_x_at | Os10g0401000 Conserved hypothetical protein. | 6.04 |  |  | 0.49 |  |  |
| Os.11299.1.S1_x_at | Os10g0560500 Hypothetical protein. | 5.91 |  | 2.33 |  | 2.83 | 2.23 |
| Os.17356.1.A1_a_at | Os11g0256900 Hypothetical protein. | 5.66 |  | 3.52 | 3.41 | 0.26 | 0.23 |
| Os.6591.1.S1_at | Os02g0798300 Conserved hypothetical protein. | 7.16 |  |  |  |  |  |
| Os.51147.1.S1_at | Os03g0277700 Protein of unknown function DUF26 domain containing protein. | 5.74 |  |  |  |  |  |
| Os.53796.1.S1_at | Os04g0413900 Conserved hypothetical protein. | 5.39 |  |  |  |  |  |
| Os.53497.1.S1_at | Os04g0472100 Hypothetical protein. | 11.72 |  |  |  |  |  |
| Os.12273.1.S1_at | Os04g0668300 Conserved hypothetical protein. | 10.96 |  |  |  |  |  |
| Os.12275.1.S1_at | Os04g0668400 Conserved hypothetical protein. | 32.40 |  |  |  |  |  |
| Os.55257.1.S1_at | Os05g0203000 Conserved hypothetical protein. | 5.82 |  |  |  |  |  |
| Os.11961.1.S1_at | Os05g0454500 Hypothetical protein. | 7.11 |  |  | 0.34 | 0.47 |  |
| Os.22621.1.S1_at | LOC_Os12g11990 expressed protein | 16.63 |  | 3.22 | 4.80 |  |  |
| Os.30428.1.S1_at | Os01g0288800 Conserved hypothetical protein. | 26.12 |  |  |  |  |  |
| Os.9827.1.S1_at | Os01g0550800 Protein of unknown function DUF239 domain containing protein. | 33.87 |  |  |  | 0.06 | 0.02 |
| Os.46029.1.A1_at | Os01g0681700 Conserved hypothetical protein. | 7.63 |  |  |  |  |  |
| Os.55436.1.S1_at | Os01g0800800 Hypothetical protein. | 6.06 |  |  |  | 4.14 |  |
| Os.11067.1.S1_at | Os02g0127400 Conserved hypothetical protein. | 18.98 |  |  |  |  |  |
| Os.9952.1.S1_at | Os02g0188600 Conserved hypothetical protein. | 12.32 |  |  |  |  |  |
| Os.52377.1.S1_s_at | Os02g0227100 Conserved hypothetical protein. | 8.93 |  | 4.49 |  |  |  |
| Os.50831.1.S1_at | Os02g0527200 Conserved hypothetical protein. | 9.14 | 3.35 | 2.42 |  | 0.36 |  |
| Os.9283.1.S1_at | Os02g0533800 Conserved hypothetical protein. | 12.78 |  | 3.19 |  |  |  |
| Os.54416.1.S1_at | Os02g0623300 Conserved hypothetical protein. | 6.83 |  |  |  | 0.13 | 0.06 |
| Os.38164.1.S1_at | Os12g0569700 Hypothetical protein. | 5.61 | 3.81 | 3.48 | 2.22 |  |  |
| OsAffx.31409.1.S1_s_at | Unknown | 10.31 | 3.32 | 4.58 |  | 0.15 | 0.19 |
| OsAffx.23673.1.A1_at | Unknown | 5.23 |  |  |  |  | 2.50 |
| Os.27430.1.S1_at | Unknown | 6.73 | 3.90 |  |  |  | 4.10 |
| Os.25748.1.S1_at | Unknown | 5.23 |  |  |  |  |  |
| Os.17958.1.S1_at | Unknown | 38.51 |  |  |  |  |  |
| Os.12764.1.S1_at | Unknown | 21.05 |  |  |  |  |  |
| OsAffx.28114.2.A1_at | Unknown | 18.23 |  |  |  |  |  |
| OsAffx.28114.3.A1_at | Unknown | 27.83 |  |  |  |  |  |
| Os.10255.1.S1_s_at | Unknown | 6.31 |  |  |  |  |  |
