# Supplementary material for: Genome-wide temporal-spatial gene expression profiling of drought responsiveness in rice
Source: BMC Genomics. 2011 Mar 16;12:149. doi: 10.1186/1471-2164-12-149 (PMC3070656; doi:10.1186/1471-2164-12-149)
Supplement: Additional file 15 — Panicle-specific down-regulated genes under drought stress. Excel file containing all specific down-regulated genes by drought in panicle [file 1471-2164-12-149-S15.DOC]

**Additional file 15. Panicle specific down-regulated genes under drought stress.**

| **Gene ID** | **Annotation** | **BP** | **BL** | **PL** | **TL** | **PR** | **TR** |
| --- | --- | --- | --- | --- | --- | --- | --- |
| OsAffx.27652.1.S1_x_at | Os06g0266800 GAST1 protein precursor. | 0.19 |  |  |  | 0.10 | 0.09 |
| Os.5079.1.S1_at | Os05g0153000 Gelsolin family protein. | 0.11 |  |  |  |  |  |
| Os.168.1.S1_at | Os10g0484800 Anther-specific protein YY2. | 0.11 |  |  |  |  |  |
| Os.18533.1.S1_at | Os04g0543700 Meiotic serine proteinase (related to anther development). | 0.05 |  |  |  |  |  |
| Os.7098.1.S1_at | Os05g0108600 Cysteine proteinase (related apoptosis). | 0.13 |  |  |  |  |  |
| Os.7622.1.S1_at | Os04g0527000 GRAM domain containing protein. | 0.18 |  |  |  |  |  |
| Os.46852.1.S1_at | Os10g0563900 Cyclin, N-terminal domain containing protein. | 0.09 |  |  |  |  | 0.39 |
| Os.17189.1.S1_at | Os05g0516800 Ras-related protein RIC2. | 0.14 |  |  | 0.27 | 0.36 | 0.47 |
| Os.49604.1.S1_at | Os05g0543100 Clathrin adaptor complex, medium chain family protein. | 0.17 |  |  |  | 0.33 | 0.46 |
| Os.55671.1.S1_at | Os06g0652200 Hly-III related proteins family protein. | 0.10 |  | 5.96 |  |  | 0.22 |
| Os.9420.1.S1_at | Os07g0257200 OsNramp1 (Integral membrane protein). | 0.06 |  |  | 0.30 | 0.26 | 0.26 |
| OsAffx.20496.1.S1_s_at | LOC_Os10g19960 beta-galactosidase precursor, putative, expressed | 0.12 |  |  |  |  |  |
| Os.14358.1.S1_at | Os02g0219200 Beta-galactosidase precursor (EC 3.2.1.23) (Lactase). | 0.12 |  |  |  |  | 0.39 |
| Os.10206.1.S1_at | Os09g0422500 Cellulose synthase. | 0.17 |  |  | 0.36 | 0.46 | 0.46 |
| Os.10926.1.S1_at | Os07g0252400 Cellulose synthase-8. | 0.18 |  | 0.31 | 0.32 | 0.36 | 0.28 |
| Os.18724.1.S1_a_at | Os01g0750300 Cellulose synthase CesA-2 (Fragment). | 0.19 |  |  | 0.32 |  |  |
| Os.53812.1.S1_at | Os03g0413400 Glycosyl transferase, family 8 protein. | 0.13 |  |  |  | 0.34 | 0.48 |
| Os.25298.1.S1_at | Os07g0681700 Glycosyl transferase, family 8 protein. | 0.19 |  |  |  |  |  |
| Os.51101.1.S1_at | Os07g0694400 Glycosyl transferase, family 43 protein. | 0.18 | 0.37 | 0.38 | 0.27 |  | 0.30 |
| Os.52870.1.S1_at | LOC_Os11g34710 tartrate-resistant acid phosphatase type 5 precursor, putative, expressed | 0.10 |  |  |  |  |  |
| Os.20313.1.S1_s_at | Os01g0952000 Rhicadhesin receptor precursor (Germin-like protein). | 0.09 |  | 0.49 | 0.32 |  |  |
| Os.8482.1.S1_s_at | Os04g0644400 Proline-rich-like protein. | 0.05 |  |  |  |  |  |
| Os.21524.2.S1_x_at | LOC_Os10g05860 proline-rich protein, putative, expressed; LOC_Os10g05840 expressed protein | 0.08 |  |  |  |  |  |
| Os.19281.1.S1_at | Os02g0552000 Transmembrane protein TM9SF3 (Fragment). | 0.19 |  |  |  | 0.40 |  |
| Os.47560.1.S1_at | Os12g0163700 Actin 7 (Actin 2). | 0.04 |  |  |  |  | 0.31 |
| Os.7242.1.S1_at | Os07g0175600 Plant lipid transfer/seed storage/trypsin-alpha amylase inhibitor domain containing protein. | 0.15 | 5.69 | 10.10 | 3.78 |  | 0.24 |
| OsAffx.26492.1.S1_at | Os04g0554800 Plant lipid transfer/seed storage/trypsin-alpha amylase inhibitor domain containing protein. | 0.06 |  |  |  |  |  |
| Os.18599.1.S1_at | Os03g0793800 Plant lipid transfer/seed storage/trypsin-alpha amylase inhibitor domain containing protein. | 0.10 | 5.89 | 4.06 |  | 0.37 | 0.28 |
| Os.55275.1.S1_s_at | Os03g0357500 Plant lipid transfer/seed storage/trypsin-alpha amylase inhibitor domain containing protein. | 0.08 |  |  |  |  |  |
| Os.13246.1.S1_at | Os01g0814100 Plant lipid transfer/seed storage/trypsin-alpha amylase inhibitor domain containing protein. | 0.12 |  |  |  |  |  |
| Os.18388.3.S1_at | Os01g0691300 Plant lipid transfer/seed storage/trypsin-alpha amylase inhibitor domain containing protein. | 0.04 |  |  |  |  |  |
| Os.6727.1.S1_at | Os05g0277500 Rhicadhesin receptor precursor (Germin-like protein,cell wall extensibility). | 0.15 |  |  |  |  |  |
| OsAffx.16482.1.S1_x_at | Os07g0480800 Cellulase (EC 3.2.1.4). | 0.16 |  | 0.49 |  | 0.41 |  |
| Os.18421.1.S1_a_at | Os03g0108300 Cellulase (EC 3.2.1.4). | 0.07 |  |  |  |  |  |
| Os.50640.1.S1_at | Os04g0413500 Cell wall invertase 2. | 0.20 |  |  |  |  |  |
| Os.16138.1.S1_s_at | Os03g0112900 Aldehyde 5-hydroxylase (Lignin biosynthesis). | 0.04 |  |  |  |  |  |
| Os.5638.1.S1_at | Os03g0273200 Laccase (EC 1.10.3.2). | 0.09 |  |  |  |  |  |
| Os.9615.1.S1_at | Os01g0842500 Laccase (EC 1.10.3.2). | 0.05 |  |  |  | 2.63 | 2.22 |
| Os.51049.1.S1_at | Os05g0458600 Laccase (EC 1.10.3.2). | 0.08 |  |  |  |  | 2.58 |
| Os.37295.1.S1_at | Os11g0116300 Chalcone-flavanone isomerase family protein. | 0.12 |  |  | 0.41 | 3.53 |  |
| Os.52445.1.S1_at | Os04g0553500 Pectinesterase family protein. | 0.20 |  |  |  |  |  |
| Os.47317.1.S1_at | Os04g0613200 Pectin lyase-like family protein. | 0.12 |  |  | 0.35 |  | 0.41 |
| Os.7637.1.S1_at | Os07g0119400 Pectinesterase like protein. | 0.08 |  |  |  | 0.41 | 0.24 |
| Os.11456.1.S1_at | Os07g0675000 Acyl-CoA dehydrogenase, middle and N-terminal domain containing protein.; Os07g0675100 Pectinesterase (EC 3.1.1.11) (Pectin methylesterase) (PE). | 0.19 |  |  |  |  |  |
| Os.20317.1.S1_at | Os08g0450100 Pectinesterase (EC 3.1.1.11) (Fragment). | 0.19 |  |  |  |  |  |
| Os.37692.1.S1_at | Os07g0634600 Pectinacetylesterase precursor. | 0.18 |  |  |  |  |  |
| Os.52091.1.S1_at | Os10g0550500 Pectinacetylesterase. | 0.17 |  |  |  |  |  |
| Os.46472.1.S1_at | Os10g0160100 Glycoside hydrolase, family 17 protein. | 0.17 |  |  |  |  | 0.23 |
| Os.51053.1.S1_at | Os11g0462100 Glycoside hydrolase, family 18 protein. | 0.11 |  |  |  |  |  |
| OsAffx.31336.1.S1_at | Os11g0577800 Glycoside hydrolase, family 17 protein. | 0.06 |  |  |  |  |  |
| Os.9759.2.S1_at | Os05g0542800 Glycoside hydrolase, family 28 protein. | 0.13 |  | 17.06 |  |  |  |
| Os.26716.1.S1_at | Os05g0415700 Glycoside hydrolase, family 20 protein. | 0.11 |  |  |  |  |  |
| Os.51174.1.S1_at | Os03g0239000 Glycoside hydrolase, family 16 domain containing protein. | 0.04 |  |  |  |  |  |
| OsAffx.3061.1.S1_x_at | Os02g0781000 Glycoside hydrolase, family 28 protein. | 0.18 |  |  | 0.37 |  |  |
| Os.29747.2.S1_at | Os02g0596200 Glycoside hydrolase, family 5 protein. | 0.08 |  |  |  |  |  |
| Os.27198.1.S1_at | Os02g0256100 Glycoside hydrolase, family 28 protein. | 0.17 |  |  |  |  |  |
| Os.23472.1.A1_at | Os01g0891000 Glycoside hydrolase, family 20 protein. | 0.14 |  |  |  |  |  |
| Os.2075.1.S1_at | Os01g0296700 Glycoside hydrolase, family 3, C-terminal domain containing protein. | 0.04 |  |  |  | 0.29 | 0.21 |
| Os.46743.3.S1_a_at | Os03g0747900 Myosin heavy chain class XI E1 protein. | 0.18 |  |  |  |  |  |
| OsAffx.13955.1.S1_at | Os04g0310800 AMP-dependent synthetase and ligase domain containing protein. | 0.09 |  |  |  |  |  |
| Os.10399.1.S1_at | Os05g0114000 PRLI-interacting factor F (Fragment). | 0.07 |  |  | 0.32 | 0.23 | 0.35 |
| Os.11867.2.S1_at | Os05g0410200 Esterase/lipase/thioesterase domain containing protein. | 0.17 |  |  |  |  | 0.29 |
| Os.21839.1.S1_at | Os06g0214800 Esterase/lipase/thioesterase domain containing protein. | 0.09 |  | 0.35 | 0.36 |  | 0.49 |
| Os.56189.1.S1_at | Os07g0606800 Esterase/lipase/thioesterase domain containing protein. | 0.12 |  |  |  |  |  |
| Os.22819.1.S1_at | Os08g0475400 Esterase/lipase/thioesterase domain containing protein. | 0.11 |  |  |  | 0.35 |  |
| Os.50916.1.S1_at | Os11g0138900 Esterase/lipase/thioesterase domain containing protein. | 0.17 |  |  |  |  |  |
| Os.26922.1.S1_at | Os02g0134400 L-aspartate oxidase family protein. | 0.17 |  |  |  |  | 0.31 |
| Os.51228.2.A1_at | Os03g0245100 Dihydrodipicolinate reductase family protein | 0.05 |  |  |  |  |  |
| Os.47792.1.S1_at | Os02g0106100 Fructosyltransferase. | 0.13 |  |  |  |  |  |
| Os.12866.1.S1_at | Os02g0714200 Pyrophosphate--fructose 6-phosphate 1-phosphotransferase alpha subunit | 0.20 |  |  |  | 0.49 |  |
| Os.38849.1.S1_at | Os02g0752200 Beta-D-xylosidase. | 0.14 |  |  |  |  |  |
| Os.49640.1.S1_at | Os02g0820400 Transferase family protein. | 0.19 | 0.46 | 0.26 |  | 0.45 | 0.38 |
| Os.13559.1.S1_at | Os03g0165400 Relative to SR12 protein (Fragment). | 0.08 |  |  |  | 0.47 |  |
| Os.25677.1.S1_at | Os03g0401300 Sucrose synthase 2 (EC 2.4.1.13) (Sucrose-UDP glucosyltransferase 2). | 0.14 |  |  | 0.34 | 0.30 | 0.34 |
| Os.32141.1.S1_at | Os03g0703100 Beta-glucosidase. | 0.14 |  |  |  | 0.31 | 0.37 |
| Os.5016.1.S1_at | Os03g0757900 UDP-glucose 6-dehydrogenase (EC 1.1.1.22) (UDP-Glc dehydrogenase) (UDP-GlcDH) (UDPGDH). | 0.12 |  |  |  | 0.39 | 0.31 |
| OsAffx.25765.1.S1_at | Os03g0808000 Polygalacturonase PG1. | 0.03 |  |  |  |  |  |
| Os.27231.1.S1_at | Os03g0833100 Glucose/ribitol dehydrogenase family protein. | 0.10 |  |  |  |  |  |
| Os.9860.1.S1_at | Os07g0616800 Sucrose synthase 3 (EC 2.4.1.13) (Sucrose-UDP glucosyltransferase 3). | 0.13 |  |  |  |  | 0.31 |
| Os.53946.1.S1_at | Os08g0526100 Nucleotide sugar epimerase family protein. | 0.17 |  |  |  | 0.30 |  |
| Os.9389.1.A1_a_at | Os10g0524500 Mandelonitrile lyase-like protein. | 0.02 |  |  |  |  |  |
| Os.54779.1.S1_s_at | Os11g0637200 Sugar transporter protein. | 0.04 |  |  |  |  |  |
| Os.10473.1.S1_at | Os12g0443500 UDP-glucose 6-dehydrogenase (EC 1.1.1.22) (UDP-Glc dehydrogenase) (UDP-GlcDH) (UDPGDH). | 0.12 |  | 0.28 |  | 0.45 | 0.38 |
| Os.20425.1.S1_at | Os12g0443600 UDP-glucose 6-dehydrogenase (EC 1.1.1.22) (UDP-Glc dehydrogenase) (UDP-GlcDH) (UDPGDH). | 0.16 |  |  |  |  |  |
| Os.48724.1.S1_at | Os08g0401500 Mandelonitrile lyase-like protein. | 0.11 |  |  |  |  |  |
| Os.28229.1.S1_at | Os01g0728100 Lipolytic enzyme, G-D-S-L family protein. | 0.10 |  |  | 0.49 |  |  |
| Os.8707.2.A1_at | Os03g0844600 Lipolytic enzyme, G-D-S-L family protein. | 0.19 |  |  |  |  |  |
| Os.5735.1.S1_at | Os10g0463200 Lipolytic enzyme, G-D-S-L family protein. | 0.07 |  |  |  |  |  |
| Os.51006.1.S1_at | Os09g0132200 Lipolytic enzyme, G-D-S-L family protein. | 0.15 |  |  |  |  |  |
| Os.20237.1.S1_at | Os09g0132900 Lipolytic enzyme, G-D-S-L family protein. | 0.14 |  |  |  |  |  |
| Os.10772.1.S1_at | Os06g0156600 Lipolytic enzyme, G-D-S-L family protein. | 0.15 |  | 0.50 |  |  |  |
| Os.5270.1.S1_at | Os05g0518300 Lipolytic enzyme, G-D-S-L family protein. | 0.10 |  |  |  |  |  |
| Os.49174.1.S1_at | Os03g0859100 Lipolytic enzyme, G-D-S-L family protein. | 0.17 |  |  |  |  |  |
| Os.8666.1.S1_at | Os02g0816200 Lipolytic enzyme, G-D-S-L family protein. | 0.08 |  |  |  |  |  |
| Os.24700.1.A1_at | Os02g0110000 Lipolytic enzyme, G-D-S-L family protein. | 0.05 |  |  |  |  |  |
| Os.14101.3.S1_at | Os01g0827700 Lipolytic enzyme, G-D-S-L family protein. | 0.14 |  |  |  |  | 0.28 |
| Os.14770.1.S1_at | Os03g0286500 RNA-binding region containing protein 1 (HSRNASEB) | 0.12 |  |  |  | 0.22 | 0.24 |
| Os.6313.1.S1_at | Os04g0613700 UTP--glucose-1-phosphate uridylyltransferase family protein. | 0.09 |  |  | 0.26 | 0.30 | 0.30 |
| Os.10034.1.S1_at | Os07g0624700 UMP/CMP kinase a (EC 2.7.1.48). | 0.14 |  |  |  | 0.40 | 0.41 |
| OsAffx.30792.2.S1_at | Os11g0126400 Nucleoside phosphatase GDA1/CD39 family protein. | 0.10 |  |  |  |  |  |
| Os.10695.1.S1_at | Os02g0778400 UMP/CMP kinase a (EC 2.7.1.48). | 0.12 |  |  |  |  |  |
| Os.14134.1.S1_at | Os03g0178400 Alpha/beta hydrolase family protein. | 0.10 |  | 0.23 | 0.32 |  |  |
| Os.1678.1.S1_at | Os01g0168800 Alpha/beta hydrolase family protein. | 0.19 |  |  | 0.36 | 0.46 |  |
| Os.52940.1.S1_at | Os12g0152100 Alpha/beta hydrolase fold domain containing protein. | 0.17 |  |  |  |  |  |
| OsAffx.23414.1.S1_at | Os01g0370200 Glutathione-S-transferase 19E50. | 0.20 |  |  |  |  |  |
| Os.6442.1.S1_at | Os03g0186900 Peptidase A1, pepsin family protein. | 0.16 |  |  |  | 0.25 | 0.22 |
| OsAffx.13633.1.S1_s_at | Os04g0121100 Peptidase S8 and S53, subtilisin, kexin, sedolisin domain containing protein. | 0.14 | 0.36 |  |  |  |  |
| Os.49108.2.S1_x_at | Os04g0396800 Peptidase S10, serine carboxypeptidase family protein. | 0.17 |  |  |  |  |  |
| Os.47386.1.S1_at | Os09g0423500 Peptidase A1, pepsin family protein. | 0.15 | 0.43 |  |  | 0.38 | 0.43 |
| Os.54534.1.S1_at | Os11g0215400 Peptidase A1, pepsin family protein. | 0.12 |  |  |  |  |  |
| Os.17663.1.S1_at | Os01g0868900 Proteinase inhibitor I9, subtilisin propeptide domain containing protein. | 0.06 |  |  |  |  |  |
| OsAffx.24631.1.S1_at | Os02g0597700 Ubiquitin domain containing protein. | 0.18 |  |  |  |  |  |
| Os.24364.1.A1_at | Os02g0755000 Putative methyltransferase DUF248 family protein. | 0.17 |  |  |  | 0.40 | 0.43 |
| Os.30518.1.S1_at | Os03g0114400 AAA ATPase, central region domain containing protein. | 0.20 |  |  |  | 0.38 | 0.44 |
| Os.25227.1.S1_s_at | Os01g0736400 Aminotransferase, class I and II domain containing protein.; Os01g0736300 UDP-glucuronosyl/UDP-glucosyltransferase family protein. | 0.18 |  |  | 3.06 |  |  |
| Os.54563.1.S1_at | Os03g0605300 Subtilisin-like protease (Fragment). | 0.11 | 0.36 | 0.36 | 0.25 | 0.49 | 0.45 |
| Os.49086.1.S1_at | Os03g0687700 Ribosome-inactivating protein family protein. | 0.06 |  |  |  |  |  |
| Os.22408.1.S1_at | Os07g0556800 Ribosome-inactivating protein family protein. | 0.06 |  |  |  |  |  |
| Os.12835.1.S1_at | Os07g0614500 Elongation factor 1-beta (EF-1-beta).; Os09g0525500 YY1 protein precursor. | 0.08 |  |  | 5.37 |  |  |
| Os.37067.1.S1_at | Os10g0521900 Rhomboid-like protein family protein. | 0.14 |  |  |  | 0.21 | 0.32 |
| Os.49268.1.S1_at | Os02g0575200 DNA/pantothenate metabolism flavoprotein, C-terminal domain containing protein. | 0.08 |  |  |  |  |  |
| Os.49872.1.S1_at | Os03g0140100 Cytochrome P450 family protein. | 0.19 |  | 0.22 |  |  |  |
| Os.48829.1.A1_at | Os03g0140200 Cytochrome P450 86A1 | 0.20 |  | 0.30 |  |  |  |
| Os.5752.1.S1_s_at | Os03g0168600 Cytochrome P451 family protein. | 0.10 |  |  |  |  |  |
| Os.54780.1.S1_at | Os04g0570600 Cytochrome P450 family protein. | 0.08 |  |  |  |  |  |
| Os.49633.1.S1_at | Os08g0131100 Cytochrome P450. | 0.03 |  |  |  |  |  |
| OsAffx.15160.1.S1_at | LOC_Os05g48890 fasciclin-like arabinogalactan protein 7 precursor, putative, expressed | 0.12 |  |  |  | 0.29 | 0.21 |
| Os.52641.1.S1_at | LOC_Os09g30486 fasciclin-like arabinogalactan protein 7 precursor, putative, expressed | 0.06 |  |  | 0.24 | 0.38 | 0.37 |
| Os.53242.1.S1_at | Os02g0758800 Plastocyanin-like domain containing protein. | 0.19 |  | 2.49 |  |  |  |
| Os.49129.1.S1_at | Os04g0412200 Ferredoxin I. | 0.18 | 0.44 | 0.22 |  |  |  |
| Os.33316.1.S1_at | Os01g0529800 Very-long-chain fatty acid condensing enzyme CUT1 | 0.12 |  |  |  |  |  |
| Os.50023.1.S1_at | Os02g0834300 Allergen V5/Tpx-1 related family protein. | 0.17 |  |  |  |  |  |
| Os.9211.1.S1_at | Os07g0645000 Allergen V5/Tpx-1 related family protein. | 0.19 |  | 0.26 | 0.21 | 0.35 |  |
| Os.8600.1.S1_at | Os04g0447600 NADPH-dependent codeinone reductase (EC 1.1.1.247). | 0.14 |  |  |  | 0.43 | 0.35 |
| Os.54795.1.S1_at | Os04g0573100 Glucose-methanol-choline oxidoreductase domain containing protein. | 0.05 |  |  |  |  |  |
| Os.54800.1.S1_at | Os06g0607700 ABC transporter related domain containing protein. | 0.06 |  |  |  |  |  |
| OsAffx.30689.1.S1_at | Os10g0508000 Multicopper oxidase, type 1 family protein. | 0.17 |  |  |  |  |  |
| Os.50838.1.A1_at | Os04g0656800 Peroxidase2 precursor (EC 1.11.1.7). | 0.15 |  |  |  | 0.27 | 0.22 |
| Os.153.1.S1_at | Os05g0499300 Peroxidase precursor (EC 1.11.1.7). | 0.07 |  |  |  | 0.44 | 0.22 |
| Os.5034.1.S1_at | Os07g0676900 Peroxidase (EC 1.11.1.7). | 0.04 | 0.33 |  | 4.41 |  | 0.24 |
| Os.5099.1.S1_at | Os10g0536700 Plant peroxidase family protein. | 0.13 | 2.57 |  |  |  |  |
| Os.2427.1.S1_at | Os11g0210100 Peroxidase 43 precursor (EC 1.11.1.7) (Atperox P43). | 0.12 |  |  |  |  |  |
| Os.11563.1.S1_at | Os11g0661600 Peroxidase precursor (EC 1.11.1.7). | 0.15 |  |  |  |  |  |
| Os.6763.1.S1_x_at | Os01g0678800 Heavy metal transport/detoxification protein domain containing protein. | 0.18 |  |  |  | 0.34 | 0.25 |
| Os.42420.1.S1_at | Os01g0850800 Multicopper oxidase, type 1 domain containing protein. | 0.19 |  |  |  | 0.27 | 0.23 |
| Os.51744.1.A1_at | Os08g0108300 Pistil-specific extensin-like protein family protein.; Os08g0108200 FAS2 (Fragment). | 0.16 |  |  |  |  | 0.41 |
| Os.11477.1.S1_at | Os03g0850900 Chemocyanin precursor (Basic blue protein) (Plantacyanin). | 0.08 |  |  |  | 2.76 | 2.51 |
| Os.37454.1.S1_at | Os01g0566500 Dioxygenase RAMOSUS1. | 0.11 |  |  |  |  |  |
| OsAffx.31542.1.S1_x_at | Os12g0108100 Ser Thr specific protein kinase-like protein. | 0.20 |  | 0.48 | 0.22 |  |  |
| Os.12804.1.S1_at | Os10g0516200 Protein kinase domain containing protein. | 0.13 |  | 0.28 | 0.40 | 0.40 |  |
| Os.32680.1.S1_at | Os07g0145400 Protein kinase domain containing protein. | 0.15 |  |  |  | 0.42 | 0.33 |
| Os.5753.1.S1_at | Os04g0654600 Protein kinase domain containing protein. | 0.17 |  |  |  | 0.34 | 0.32 |
| Os.43021.1.S1_at | Os01g0957100 Protein kinase domain containing protein. | 0.14 |  |  |  | 0.37 | 0.32 |
| Os.1986.1.S1_at | Os01g0738300 Protein kinase domain containing protein. | 0.18 |  |  |  | 0.33 | 0.39 |
| Os.36194.1.S1_at | Os01g0243100 Kinesin heavy chain (calmodulin binding proteint). | 0.07 |  |  |  | 5.43 |  |
| Os.35495.1.S1_at | Os01g0265100 NTGB1 (Fragment). | 0.13 |  |  |  |  |  |
| Os.20579.1.S1_at | Os01g0727800 Protease-associated PA domain containing protein (Cellular processes and signaling). | 0.18 |  |  |  | 0.47 | 0.35 |
| Os.10107.1.S1_at | Os04g0683600 Receptor kinase-like protein. | 0.20 | 4.55 | 2.88 |  |  | 0.50 |
| Os.6815.1.S1_at | Os06g0714600 Ras GTPase family protein. | 0.17 |  | 7.87 |  | 0.29 | 0.22 |
| Os.6814.2.S1_s_at | Os11g0109000 Protein phosphatase 2C-like domain containing protein. | 0.17 |  |  |  |  |  |
| Os.21805.1.S1_s_at | Os06g0729400 Gibberellin-regulated protein 2 precursor. | 0.09 |  |  |  |  |  |
| Os.46073.1.A1_at | Os04g0404400 Bipartite response regulator, C-terminal effector domain containing protein. | 0.12 |  | 35.19 |  | 3.52 |  |
| Os.33745.1.S1_at | Os01g0801500 Beta-1,3-glucanase precursor. | 0.11 |  |  |  |  |  |
| Os.8643.1.S1_at | Os03g0250200 Pathogenicity protein PATH531-like protein. | 0.10 |  |  | 0.28 |  | 0.37 |
| OsAffx.3726.1.S1_at | Os04g0137100 Pectate lyase (Fragment). | 0.13 |  |  |  |  | 0.50 |
| Os.49653.1.S1_at | Os06g0133600 Phosphate-induced protein 1 conserved region family protein. | 0.19 |  |  | 0.44 |  |  |
| Os.12381.1.S1_s_at | Os07g0636600 Plant disease resistance response protein family protein. | 0.09 |  |  | 5.36 |  |  |
| OsAffx.23175.1.S1_x_at | Os01g0195400 Harpin-induced 1 domain containing protein. | 0.09 |  |  | 0.22 | 0.33 |  |
| Os.31042.1.S1_at | Os07g0250900 Harpin-induced 1 domain containing protein. | 0.15 |  |  |  |  |  |
| Os.7551.1.S1_at | Os01g0942300 Glucan endo-1,3-beta-glucosidase, acidic isoform precursor | 0.07 |  |  |  |  |  |
| Os.53973.1.S1_at | Os02g0733300 Endo-beta-1,4-glucanase precursor (EC 3.2.1.4). | 0.19 |  |  |  |  |  |
| Os.16862.1.S1_at | Os03g0117300 Beta-glucanase family protein. | 0.17 |  |  | 0.44 | 0.36 |  |
| OsAffx.22670.1.S1_s_at | Os03g0772000 Beta glucanase precursor (EC 3.2.1.73) (Fragment). | 0.17 |  |  |  |  |  |
| Os.4160.1.S1_at | Os05g0495900 Beta-1,3-glucanase precursor (Fragment). | 0.16 |  |  |  |  |  |
| OsAffx.27621.1.S1_s_at | Os06g0247000 Avr9/Cf-9 rapidly elicited protein 231. | 0.14 |  |  |  | 0.27 | 0.28 |
| Os.36913.1.S1_at | Os10g0552800 RCc3 protein (root specific, gibberellic acid–induced gene). | 0.06 |  |  |  |  | 0.36 |
| Os.14195.1.S1_at | Os03g0304500 Heat shock protein DnaJ, N-terminal domain containing protein. | 0.14 |  | 2.37 |  | 0.46 | 0.40 |
| Os.34478.1.S1_at | Os07g0689800 Heat shock protein DnaJ, N-terminal domain containing protein. | 0.14 |  |  |  |  |  |
| Os.6291.1.S1_at | Os06g0691200 Thaumatin-like protein precursor. | 0.12 |  |  |  | 0.32 | 0.37 |
| Os.49997.1.S1_at | Os05g0215000 BURP domain containing protein. | 0.17 |  | 0.24 |  |  |  |
| Os.48467.1.S1_at | Os05g0406800 Leucine-rich repeat, plant specific containing protein. | 0.15 |  |  |  |  |  |
| Os.6800.1.S1_x_at | Os02g0830700 Leucine-rich repeat, plant specific containing protein. | 0.17 |  |  |  |  |  |
| Os.4614.1.S1_a_at | Os02g0616100 Leucine-rich repeat, plant specific containing protein. | 0.11 |  |  |  |  |  |
| OsAffx.14585.1.S1_s_at | Os05g0170300 Leucine-rich repeat, typical subtype containing protein. | 0.12 |  |  |  |  | 0.49 |
| Os.30567.1.S1_at | Os01g0645000 TIS11 protein (dTIS11). | 0.08 |  |  |  | 0.38 | 0.34 |
| Os.32224.1.S1_at | Os01g0761400 TGF-beta receptor, type I/II extracellular region family protein. | 0.18 |  |  |  |  |  |
| Os.52097.1.S1_at | Os04g0526000 PCF1. | 0.05 |  |  |  |  |  |
| Os.37618.1.S1_at | Os06g0140400 DNA-binding protein (Homeodomain-leucine zipper transcription factor). | 0.07 |  |  |  |  | 0.28 |
| Os.2365.1.S1_at | Os06g0140700 Homeobox-leucine zipper protein HAT14 (HD-ZIP protein 14). | 0.11 |  |  | 0.39 |  | 0.39 |
| Os.50830.1.S1_at | Os09g0414500 ZF-HD homeobox protein. | 0.20 |  |  |  |  |  |
| Os.48051.1.S1_at | Os09g0454300 Cyclin-like F-box domain containing protein. | 0.16 |  |  |  |  |  |
| Os.56229.1.S1_at | Os11g0138700 Cyclin-like F-box domain containing protein. | 0.15 |  |  |  |  |  |
| Os.7784.1.S1_at | Os12g0510900 Zn-binding protein, LIM domain containing protein. | 0.20 |  |  |  | 0.43 | 0.47 |
| Os.52203.1.S1_at | Os12g0547500 Kinesin, motor region domain containing protein. | 0.13 |  |  |  |  |  |
| Os.8374.1.S1_at | Os04g0519700 Auxin response factor 10. | 0.16 |  |  |  | 0.35 |  |
| Os.7611.1.S1_at | Os03g0162200 Histone H2A. | 0.19 |  |  |  | 0.35 | 0.28 |
| Os.12821.1.S1_at | Os03g0279200 Histone H2A. | 0.17 |  | 0.44 |  | 0.39 | 0.34 |
| Os.10246.4.S1_x_at | Os06g0160100 Histone H3. | 0.15 |  |  |  | 0.30 | 0.36 |
| Os.10246.2.S1_at | Os05g0438700 Histone H3. | 0.17 |  |  |  | 0.25 | 0.31 |
| Os.10246.5.S1_at | Os01g0866200 Histone H3. | 0.11 |  |  |  | 0.21 | 0.26 |
| Os.3391.1.S1_at | Os09g0401000 Myb-related protein Pp2. | 0.16 |  |  |  |  |  |
| Os.47854.1.S1_at | Os02g0695200 P-type R2R3 Myb protein (Fragment). | 0.17 |  |  |  |  |  |
| Os.33336.1.S1_at | Os01g0211800 Basic-leucine zipper (bZIP) transcription factor domain containing protein. | 0.19 |  |  |  | 0.39 |  |
| Os.16077.1.S1_at | Os02g0790600 Zn-finger, RING domain containing protein. | 0.09 |  |  |  |  | 0.27 |
| Os.55716.1.S1_at | Os04g0162500 Zn-finger, C2H2 type domain containing protein. | 0.16 |  | 0.23 | 0.44 | 0.33 | 0.34 |
| Os.7830.1.S1_at | Os04g0243700 Zn-finger, RING domain containing protein. | 0.18 |  | 2.65 | 0.28 |  |  |
| Os.23220.1.S1_at | Os05g0436900 Zn-finger, DHHC type domain containing protein. | 0.17 |  |  |  |  | 0.26 |
| Os.54644.1.S1_at | Os05g0576300 Zn-finger, C-x8-C-x5-C-x3-H type domain containing protein. | 0.19 |  |  |  |  |  |
| Os.46573.1.S1_at | Os10g0438800 Zn-finger, RING domain containing protein. | 0.11 |  |  |  |  |  |
| Os.54858.1.S1_at | Os10g0555300 Zn-finger, C2H2 type domain containing protein. | 0.20 |  |  |  |  |  |
| Os.23040.1.S1_at | Os01g0623200 C4-dicarboxylate transporter/malic acid transport protein family protein. | 0.19 |  | 6.25 |  |  |  |
| Os.46160.2.S1_at | Os10g0444700 Phosphate transporter 6. | 0.17 |  | 0.23 | 3.68 |  |  |
| Os.2358.1.S1_at | Os08g0466200 K+ potassium transporter family protein. | 0.12 |  |  |  |  |  |
| Os.7619.1.S1_s_at | Os06g0671800 Cellular retinaldehyde binding/alpha-tocopherol transport family protein. | 0.09 |  |  |  | 0.40 | 0.25 |
| Os.8422.1.S1_at | Os01g0908600 Amino acid/polyamine transporter II family protein. | 0.17 |  |  |  |  |  |
| Os.11846.1.S1_at | Os05g0424000 Amino acid carrier (Fragment). | 0.15 | 4.83 | 2.29 | 5.85 |  | 0.44 |
| Os.27524.2.S1_at | Os09g0338500 Desaturase/cytochrome b5 protein. | 0.16 | 0.37 | 0.23 | 0.49 | 0.40 | 0.39 |
| Os.49877.1.S1_at | Os04g0545600 Uclacyanin 3-like protein. | 0.17 |  |  |  |  |  |
| Os.7393.1.S1_at | Os07g0112700 Plastocyanin-like domain containing protein. | 0.10 |  |  |  | 0.34 | 0.37 |
| OsAffx.28504.1.S1_at | Os07g0282300 Aldehyde oxidase-2.; Os07g0281700 Ferredoxin domain containing protein. | 0.15 |  |  |  |  | 0.27 |
| Os.27071.1.S1_at | Os05g0210600 TPR-like domain containing protein (function in protein import). | 0.14 |  |  |  |  | 0.40 |
| Os.31744.1.S1_at | Os01g0112400 Major intrinsic protein family protein. | 0.20 |  |  |  |  |  |
| Os.31191.1.S1_at | Os07g0448400 Plasma membrane integral protein ZmPIP2-6. | 0.13 |  |  |  |  | 0.43 |
| Os.18572.1.S1_at | Os04g0613000 Zinc transporter 1 precursor (ZRT/IRT-like protein 1). | 0.15 |  |  |  |  |  |
| Os.7855.1.S1_at | Os12g0601400 Auxin-responsive protein (Aux/IAA) (Fragment). | 0.16 |  |  | 0.48 | 0.36 | 0.22 |
| Os.9194.1.S1_at | Os02g0757100 Phi-1 protein. | 0.09 |  |  |  | 0.37 | 0.24 |
| Os.18434.1.S1_at | Os02g0619600 von Willebrand factor, type A domain containing protein. | 0.14 |  |  |  |  | 0.42 |
| Os.10102.1.S1_at | Os01g0863500 Conserved hypothetical protein. | 0.19 |  |  |  |  |  |
| Os.57090.1.S1_at | Os03g0291200 Protein of unknown function DUF231 domain containing protein. | 0.08 |  |  |  |  | 0.32 |
| Os.8145.2.S1_at | Os03g0291800 Protein of unknown function DUF231 domain containing protein. | 0.12 |  | 0.22 | 0.37 | 0.48 | 0.45 |
| Os.6965.1.S1_at | Os03g0310800 Parvalbumin family protein. | 0.16 |  | 0.23 |  | 0.35 | 0.31 |
| Os.49505.2.S1_at | Os03g0345300 Conserved hypothetical protein. | 0.04 |  |  |  |  |  |
| Os.33920.1.S1_at | Os03g0400600 Conserved hypothetical protein. | 0.16 |  |  |  | 0.44 | 0.42 |
| Os.33081.1.S1_at | Os03g0594700 Conserved hypothetical protein. | 0.19 |  | 3.18 |  |  |  |
| Os.50969.1.S1_at | Os03g0664400 Fibroin. | 0.17 |  |  |  | 0.47 | 0.27 |
| Os.8439.1.S1_at | Os03g0692700 Protein of unknown function DUF538 family protein. | 0.15 |  |  |  |  | 0.23 |
| Os.10095.1.S1_at | Os03g0817800 Protein of unknown function DUF231 domain containing protein. | 0.13 |  |  | 0.23 |  | 0.41 |
| Os.14285.2.S1_at | Os04g0384800 Conserved hypothetical protein. | 0.15 |  |  | 0.23 |  |  |
| Os.47938.1.S1_at | Os04g0512300 Conserved hypothetical protein. | 0.20 |  |  | 0.40 |  |  |
| Os.20306.1.S1_at | Os04g0528200 Conserved hypothetical protein. | 0.14 |  |  | 2.45 |  |  |
| Os.52543.1.S1_at | Os04g0627400 PAK-box/P21-Rho-binding domain containing protein. | 0.16 |  |  |  |  | 0.46 |
| Os.56185.1.S1_at | Os04g0629400 Conserved hypothetical protein. | 0.10 |  |  |  |  |  |
| Os.23656.1.A1_at | Os04g0672300 Conserved hypothetical protein. | 0.16 |  |  |  | 0.34 | 0.30 |
| OsAffx.14465.1.S1_s_at | Os04g0677600 Conserved hypothetical protein. | 0.08 |  |  |  |  |  |
| Os.51828.1.S1_at | Os05g0122600 Conserved hypothetical protein. | 0.16 |  | 0.43 | 0.48 |  |  |
| Os.10482.1.S1_at | Os05g0366900 Conserved hypothetical protein. | 0.20 |  |  |  |  |  |
| Os.54789.1.S1_at | Os05g0376800 GASA5-like protein (Fragment). | 0.08 |  |  |  |  |  |
| Os.47700.1.A1_at | Os05g0394200 Protein of unknown function DUF630 domain containing protein. | 0.20 |  |  |  | 0.46 | 0.40 |
| Os.51943.1.S1_at | Os05g0462500 Lung seven transmembrane receptor family protein. | 0.18 |  |  |  |  |  |
| Os.50276.1.S1_at | Os05g0582100 Conserved hypothetical protein. | 0.17 |  |  |  |  |  |
| Os.27301.1.S1_at | Os06g0153200 Conserved hypothetical protein. | 0.12 |  |  |  |  |  |
| Os.51692.1.S1_at | Os06g0234600 Protein of unknown function DUF231 domain containing protein. | 0.11 |  |  |  | 0.46 | 0.36 |
| Os.14208.1.S1_at | Os06g0474500 Protein of unknown function DUF239 domain containing protein. | 0.07 |  |  |  |  |  |
| Os.52241.1.S1_at | Os06g0551500 Conserved hypothetical protein. | 0.17 |  |  |  | 2.30 |  |
| Os.5208.1.S1_at | Os06g0554300 Hypothetical protein. | 0.14 |  |  | 0.25 | 0.35 | 0.35 |
| Os.9684.1.S1_at | Os06g0589500 Conserved hypothetical protein. | 0.10 |  |  | 0.22 | 0.21 | 0.21 |
| Os.56087.1.S1_at | Os06g0611100 Conserved hypothetical protein. | 0.07 |  |  |  |  |  |
| Os.57084.1.S1_at | Os06g0624700 Conserved hypothetical protein. | 0.05 |  |  |  | 2.05 |  |
| Os.19277.1.S1_at | Os07g0133500 Protein of unknown function DUF1005 family protein. | 0.19 |  |  |  |  | 0.33 |
| Os.18648.1.S1_at | Os07g0142300 Conserved hypothetical protein. | 0.17 | 2.49 | 4.43 | 2.42 | 0.36 | 0.22 |
| Os.6633.1.S1_at | Os07g0205500 Protein of unknown function DUF239 domain containing protein. | 0.18 |  |  |  |  | 0.46 |
| Os.8486.1.S2_s_at | Os07g0644200 NodH (Fragment). | 0.18 |  |  |  |  |  |
| OsAffx.28928.1.S1_at | Os07g0655800 Conserved hypothetical protein. | 0.14 |  |  |  |  |  |
| Os.9719.1.S1_at | Os07g0671100 Conserved hypothetical protein. | 0.17 |  | 0.39 |  | 0.38 | 0.28 |
| OsAffx.17184.1.S1_at | Os08g0360700 Conserved hypothetical protein. | 0.04 |  |  |  |  |  |
| Os.24594.1.A1_s_at | Os08g0390100 Protein of unknown function DUF810 family protein. | 0.18 |  |  |  |  |  |
| Os.9673.1.S1_at | Os08g0515800 Mitochodrial transcription termination factor-related family protein. | 0.19 |  |  |  | 0.25 | 0.48 |
| OsAffx.17435.1.S1_s_at | Os08g0515900 Conserved hypothetical protein. | 0.17 |  |  |  |  |  |
| Os.7601.1.S1_s_at | Os08g0546900 NC domain containing protein. | 0.14 |  |  |  |  |  |
| Os.49509.1.S1_at | Os08g0560300 Protein of unknown function DUF597 family protein. | 0.13 |  |  |  | 0.40 | 0.35 |
| Os.54831.1.S1_at | Os09g0309600 Conserved hypothetical protein. | 0.15 |  |  |  |  |  |
| Os.14294.1.S1_at | Os09g0323500 Protein of unknown function DUF833 family protein. | 0.15 |  | 68.56 |  |  | 0.25 |
| Os.48846.1.S1_at | Os09g0414900 GASA5-like protein (Fragment). | 0.03 |  |  |  |  | 0.37 |
| Os.51465.1.S1_x_at | Os09g0480100 Conserved hypothetical protein. | 0.20 |  |  |  |  |  |
| Os.8446.1.S1_at | Os09g0487200 Conserved hypothetical protein. | 0.10 |  |  |  |  |  |
| Os.2319.1.S2_at | Os09g0493500 Conserved hypothetical protein. | 0.07 |  | 4.80 |  |  |  |
| Os.51214.2.S1_at | Os09g0509400 Conserved hypothetical protein. | 0.20 |  | 4.11 |  |  | 0.48 |
| Os.52276.1.S1_at | Os09g0529300 Hypothetical protein. | 0.08 |  |  |  |  |  |
| Os.46488.1.S1_at | Os10g0148700 Protein of unknown function DUF1210 family protein. | 0.14 |  |  |  |  |  |
| Os.21524.1.S1_at | Os10g0149000 Protein of unknown function DUF1210 family protein. | 0.07 |  |  |  |  |  |
| Os.46085.1.S1_x_at | Os10g0150000 Protein of unknown function DUF1210 family protein. | 0.07 |  |  |  |  |  |
| Os.8638.1.S1_at | Os10g0150300 Protein of unknown function DUF1210 family protein. | 0.04 |  |  |  |  |  |
| Os.11398.1.S1_at | Os10g0150600 Protein of unknown function DUF1210 family protein. | 0.10 |  |  |  |  |  |
| Os.8385.2.S1_at | Os10g0150800 Protein of unknown function DUF1210 family protein. | 0.20 |  |  |  |  |  |
| Os.15856.1.S1_at | Os10g0154700 Cyclophilin Dicyp-2. | 0.14 | 4.65 | 5.01 |  | 2.42 |  |
| Os.10525.1.S1_at | Os10g0200700 Hypothetical protein. | 0.17 |  |  |  |  |  |
| Os.46580.1.S1_at | Os10g0337700 MFP1 attachment factor 1. | 0.09 | 0.35 | 0.21 |  | 0.40 | 0.27 |
| Os.53394.1.S1_at | Os10g0422800 Conserved hypothetical protein. | 0.19 |  |  |  |  |  |
| Os.2611.1.S1_a_at | Os10g0504600 Chorion 2 family protein. | 0.19 |  |  | 0.40 | 0.33 | 0.29 |
| Os.46597.2.S1_at | Os10g0509800 Conserved hypothetical protein. | 0.20 |  |  |  | 0.22 | 0.26 |
| Os.14119.2.S1_x_at | Os10g0559800 Protein of unknown function DUF547 domain containing protein. | 0.19 |  |  |  | 2.03 |  |
| OsAffx.31541.1.S1_s_at | Os11g0107600 Prenylated rab acceptor PRA1 family protein. | 0.07 |  |  |  |  | 0.49 |
| Os.55139.1.S1_at | Os11g0139400 Conserved hypothetical protein. | 0.08 |  |  |  | 0.27 | 0.25 |
| Os.15841.1.S1_a_at | Os11g0150400 Conserved hypothetical protein. | 0.08 |  | 0.37 |  | 0.47 | 0.30 |
| Os.14121.1.S1_at | Os11g0170000 Amidase family protein. | 0.11 |  |  |  |  |  |
| OsAffx.30964.1.S1_at | Os11g0242600 Uncharacterized plant-specific domain 01627 containing protein. | 0.19 |  |  |  |  |  |
| Os.51855.1.S1_at | Os11g0458100 Hypothetical protein. | 0.19 |  |  |  |  | 0.48 |
| Os.50793.1.S1_at | Os12g0109700 Hypothetical protein. | 0.13 |  |  |  |  |  |
| Os.20118.1.A1_s_at | Os12g0169000 Amidase family protein. | 0.16 | 2.05 |  |  | 0.24 | 0.25 |
| Os.25559.1.S1_at | Os12g0204500 Uncharacterized plant-specific domain 01627 containing protein. | 0.14 | 0.27 | 0.25 | 0.25 |  |  |
| Os.15807.1.S1_s_at | Os12g0507400 Hypothetical protein. | 0.12 |  |  |  |  |  |
| Os.56976.1.S1_at | Os12g0571300 Conserved hypothetical protein. | 0.14 |  |  |  |  |  |
| Os.54620.1.A1_at | Os12g0577200 Hypothetical protein. | 0.10 |  |  | 0.27 | 0.43 |  |
| Os.51699.1.S1_at | Os02g0558600 Conserved hypothetical protein. | 0.14 |  |  |  |  |  |
| Os.50743.1.S1_at | Os02g0656100 Conserved hypothetical protein. | 0.12 |  |  |  |  |  |
| Os.45916.1.S1_x_at | Os01g0251400 Hypothetical protein. | 0.19 |  |  |  |  |  |
| Os.55385.1.S1_at | Os01g0264400 Conserved hypothetical protein. | 0.19 |  |  |  | 0.49 | 0.39 |
| Os.48354.1.S1_at | Os02g0761600 Conserved hypothetical protein. | 0.13 |  |  |  |  |  |
| Os.9875.1.S1_at | Os03g0210500 Protein of unknown function DUF538 family protein. | 0.09 |  |  |  |  | 0.26 |
| Os.24515.1.S1_at | Os01g0546400 Protein of unknown function DUF6 domain containing protein. | 0.17 |  |  |  |  | 0.44 |
| Os.18001.1.S1_at | Os01g0631100 Conserved hypothetical protein. | 0.18 |  |  |  |  |  |
| Os.32162.1.S1_at | Os01g0208400 Conserved hypothetical protein. | 0.19 |  |  |  | 0.46 | 0.35 |
| Os.27509.1.S1_at | Os01g0740400 Protein of unknown function DUF1005 family protein. | 0.09 | 0.41 | 3.03 | 0.44 | 0.42 | 0.26 |
| Os.5831.1.S1_at | Os01g0756900 Conserved hypothetical protein. | 0.17 |  |  |  | 0.47 | 0.43 |
| OsAffx.24129.1.S1_at | Os02g0158500 Uncharacterized plant-specific domain 01627 containing protein. | 0.07 |  |  |  |  |  |
| Os.8984.1.S1_at | Os02g0230300 En/Spm-like transposon protein (Protodermal factor 1). | 0.08 |  |  |  |  |  |
| Os.14430.1.S1_at | Os02g0308400 Beta-Ig-H3/fasciclin domain containing protein. | 0.03 |  |  |  |  |  |
| Os.20298.1.S1_at | Os02g0101900 Hypothetical protein. | 0.04 |  |  |  |  |  |
| Os.52100.1.S1_at | LOC_Os03g56890 expressed protein | 0.04 |  |  |  |  |  |
| Os.14210.1.A1_at | LOC_Os10g36360 expressed protein | 0.16 |  |  |  |  |  |
| Os.47369.1.A1_at | LOC_Os10g39660 expressed protein | 0.17 |  |  |  |  |  |
| Os.56224.1.S1_at | Os01g0110200 Conserved hypothetical protein. | 0.09 | 0.43 | 0.25 | 0.23 | 0.50 |  |
| Os.23188.1.S1_at | Os01g0928200 Hypothetical protein. | 0.15 |  |  |  |  |  |
| OsAffx.11813.1.S1_at | Os01g0937900 Conserved hypothetical protein. | 0.18 |  |  |  | 0.28 | 0.32 |
| Os.20305.1.S1_at | Os01g0127500 Conserved hypothetical protein. | 0.02 |  |  |  |  |  |
| Os.18338.1.S1_at | Os02g0124400 Conserved hypothetical protein. | 0.18 |  |  |  |  |  |
| Os.51291.1.S1_at | Os02g0234700 Conserved hypothetical protein. | 0.19 |  |  | 0.47 | 0.39 | 0.30 |
| Os.12412.1.S1_at | Os02g0264800 Protein of unknown function DUF1070 family protein. | 0.19 |  |  |  |  |  |
| Os.11058.1.S1_x_at | Os02g0329800 Protein of unknown function DUF563 family protein. | 0.16 |  | 3.62 | 0.34 |  | 0.38 |
| Os.53579.1.S1_at | Os02g0505500 Conserved hypothetical protein. | 0.15 |  |  |  |  |  |
| Os.28213.1.S1_at | Os12g0609600 Hypothetical protein. | 0.10 |  |  |  |  |  |
| Os.24249.1.A1_at | Os12g0610800 Protein of unknown function DUF588 family protein. | 0.09 |  |  |  | 0.24 | 0.25 |
| Os.6763.1.S1_at | Unknown | 0.18 |  |  |  | 0.34 | 0.26 |
| OsAffx.9099.1.S1_x_at | Unknown | 0.16 |  |  |  |  | 0.34 |
| Os.57.1.S1_at | Unknown | 0.11 |  |  |  |  | 0.34 |
| Os.49519.1.S1_at | Unknown | 0.07 |  |  |  |  | 0.36 |
| Os.22590.1.A1_at | Unknown | 0.20 |  |  | 0.22 | 0.48 | 0.39 |
| Os.5095.1.S1_at | Unknown | 0.09 |  |  |  | 0.46 | 0.42 |
| Os.12462.1.S1_at | Unknown | 0.20 |  |  |  | 0.45 |  |
| Os.19038.1.S1_at | Unknown | 0.11 |  |  |  |  |  |
| Os.55116.1.S1_at | Unknown | 0.08 |  |  |  |  |  |
| Os.52629.1.S1_at | Unknown | 0.10 |  |  |  |  |  |
| Os.50723.1.A1_x_at | Unknown | 0.12 |  |  |  |  |  |
| Os.51088.1.S1_at | Unknown | 0.11 |  |  |  |  |  |
| Os.50725.1.A1_x_at | Unknown | 0.14 |  |  |  |  |  |
| Os.47879.1.A1_at | Unknown | 0.20 |  |  |  |  |  |
