# Supplementary material for: Genome-wide temporal-spatial gene expression profiling of drought responsiveness in rice
Source: BMC Genomics. 2011 Mar 16;12:149. doi: 10.1186/1471-2164-12-149 (PMC3070656; doi:10.1186/1471-2164-12-149)
Supplement: Additional file 16 — List of TF genes differentially regulated under drought stress. Excel file containing all transcription factor genes differentially regulated by drought stress in all samples [file 1471-2164-12-149-S16.DOC]

**Additional file 16**. The list of TF genes differentially regulated under drought stress

| **Probe ID** | **TF** | **TL** | **PL** | **BL** | **TR** | **PR** | **BP** |
| --- | --- | --- | --- | --- | --- | --- | --- |
| OsAffx.17366.1.S1_at | AP2/EREBP family | 17.83 | 16.59 | 14.00 | 10.82 |  | 18.90 |
| Os.54944.1.S1_at | AP2/EREBP family | 7.32 | 79.67 | 18.94 |  |  | 5.34 |
| Os.38447.1.S1_s_at | AP2/EREBP family |  | 8.67 | 10.13 |  |  | 8.79 |
| Os.38982.1.S1_at | AP2/EREBP family |  | 13.35 | 5.57 |  |  |  |
| OsAffx.27442.1.S1_at | AP2/EREBP family |  | 31.08 | 6.71 |  |  |  |
| Os.3766.1.S1_at | AP2/EREBP family |  |  | 5.08 |  |  |  |
| Os.7329.1.S1_s_at | AP2/EREBP family |  |  | 8.36 |  |  |  |
| OsAffx.14373.1.S1_s_at | AP2/EREBP family |  |  | 7.86 |  |  |  |
| Os.21563.1.S2_at | AP2/EREBP family |  | 25.59 |  |  |  |  |
| Os.21563.1.S1_a_at | AP2/EREBP family |  | 7.49 |  |  |  |  |
| Os.11296.1.S1_a_at | AP2/EREBP family |  | 5.94 |  |  |  |  |
| Os.6009.3.S1_a_at | AP2/EREBP family |  | 12.81 |  |  |  |  |
| Os.9523.1.S1_at | AP2/EREBP family |  | 5.37 |  |  |  |  |
| Os.11094.1.A1_at | AP2/EREBP family |  |  |  |  |  | 12.01 |
| Os.4893.1.S1_at | AP2/EREBP family |  |  |  | 0.07 | 0.04 | 7.72 |
| Os.11120.1.S1_at | AP2/EREBP family |  |  |  | 0.06 | 0.07 |  |
| Os.56665.1.A1_at | AP2/EREBP family |  |  |  | 0.16 | 0.15 |  |
| OsAffx.24232.2.S1_s_at | AP2/EREBP family |  |  |  | 0.15 | 0.18 |  |
| Os.8920.1.S1_at | AP2/EREBP family |  |  |  | 0.03 | 0.01 |  |
| Os.6539.1.S1_at | AP2/EREBP family |  |  |  | 0.18 | 0.19 |  |
| Os.8031.1.S1_at | AP2/EREBP family |  |  |  | 0.03 | 0.12 |  |
| Os.57381.1.S1_at | AP2/EREBP family |  |  |  | 0.01 | 0.03 |  |
| OsAffx.12799.1.S1_s_at | AP2/EREBP family |  |  |  |  | 0.14 |  |
| Os.5816.1.S1_at | AP2/EREBP family |  |  |  |  | 0.15 |  |
| Os.56201.1.S1_at | AP2/EREBP family |  |  |  | 0.15 |  |  |
| Os.10293.1.S1_at | AP2/EREBP family |  |  |  | 0.17 |  |  |
| Os.28406.1.S1_at | AP2/EREBP family |  |  |  | 0.20 |  |  |
| Os.55643.1.S1_at | AP2/EREBP family | 0.16 |  | 0.15 |  |  |  |
| Os.17070.1.S1_at | ARF family |  | 0.09 |  |  |  |  |
| Os.7177.2.S1_a_at | ARF family |  | 0.14 |  |  |  |  |
| Os.34249.2.S1_x_at | ARF family |  | 0.17 |  |  |  |  |
| Os.53414.1.S1_at | AS2 family | 5.55 | 14.45 |  |  |  |  |
| Os.22577.2.S1_x_at | AS2 family |  |  |  |  | 0.09 |  |
| OsAffx.22801.1.S1_at | AS2 family |  |  |  |  | 0.12 |  |
| Os.7112.1.S1_at | AS2 family |  |  |  | 0.14 |  |  |
| Os.15798.1.S1_at | AUX/IAA family |  | 7.08 | 5.25 |  |  | 5.34 |
| Os.11608.1.S1_at | AUX/IAA family | 5.56 | 14.21 | 7.09 |  |  |  |
| Os.7855.1.S1_at | AUX/IAA family |  |  |  |  |  | 0.16 |
| Os.12733.2.S1_at | AUX/IAA family |  | 0.05 |  |  |  |  |
| Os.19952.1.S1_at | AUX/IAA family |  | 0.11 |  |  |  |  |
| Os.4806.1.S1_at | AUX/IAA family |  | 0.20 |  |  |  |  |
| OsAffx.15562.1.S1_s_at | AUX/IAA family | 0.15 |  |  |  |  |  |
| Os.54781.1.S1_at | BES1 family |  | 0.15 |  |  |  | 0.15 |
| Os.27243.1.S1_at | bHLH family | 5.58 | 6.20 |  |  |  |  |
| Os.51063.1.S1_at | bHLH family |  |  | 21.14 |  |  | 5.94 |
| Os.10830.1.S1_at | bHLH family | 0.13 | 0.12 | 0.12 |  |  |  |
| Os.6043.1.S1_at | bHLH family | 11.46 | 0.06 |  |  | 0.17 |  |
| Os.20775.1.S1_at | bHLH family | 0.13 |  | 0.08 |  |  |  |
| Os.27587.1.S1_at | bHLH family |  | 0.02 | 0.13 |  |  |  |
| Os.5178.1.A1_s_at | bHLH family |  | 0.04 | 0.14 |  |  |  |
| Os.51847.1.S1_x_at | bHLH family |  |  |  | 0.11 | 0.14 |  |
| Os.7694.1.S1_at | bHLH family |  |  |  | 0.06 | 0.07 |  |
| Os.5860.1.S1_at | bHLH family |  |  |  | 0.11 | 0.13 |  |
| Os.31303.1.S1_at | bHLH family |  |  |  | 0.13 | 0.17 |  |
| Os.30044.1.S1_a_at | bHLH family |  | 0.13 |  |  |  |  |
| OsAffx.26114.1.S1_at | bHLH family |  | 0.11 |  |  |  |  |
| Os.20361.1.A1_at | bHLH family |  | 0.14 |  |  |  |  |
| Os.27705.1.S1_at | bHLH family |  | 0.16 |  |  |  |  |
| Os.21231.1.S1_at | bHLH family | 0.14 |  |  |  |  |  |
| Os.9216.1.S1_at | bHLH family | 0.10 |  |  |  |  |  |
| Os.49337.1.S1_at | bHLH family |  |  |  | 0.07 |  |  |
| OsAffx.31980.1.S1_at | bHLH family |  |  |  | 0.11 |  |  |
| Os.46600.1.S1_at | bHLH family |  |  |  | 0.19 |  |  |
| OsAffx.15125.1.S1_at | bHLH family |  |  |  | 0.11 |  |  |
| Os.411.2.S1_x_at | bZIP family | 8.39 | 13.27 | 15.04 | 11.26 | 9.55 | 6.62 |
| Os.19553.1.S1_at | bZIP family | 6.92 | 9.29 | 22.21 |  |  | 7.21 |
| Os.37925.1.S1_a_at | bZIP family |  | 5.44 | 5.25 |  |  |  |
| Os.413.1.S1_at | bZIP family |  |  | 15.12 |  |  | 8.19 |
| OsAffx.11963.1.S1_at | bZIP family |  | 22.71 | 11.31 |  |  |  |
| Os.55684.1.S1_at | bZIP family |  |  |  | 0.18 |  | 0.13 |
| Os.54299.1.S1_at | bZIP family |  |  |  | 13.86 | 19.35 |  |
| Os.1503.1.S1_at | bZIP family | 0.18 |  |  | 0.05 |  |  |
| Os.11781.1.S1_at | bZIP family |  |  | 12.20 |  |  |  |
| Os.33336.1.S1_at | bZIP family |  |  |  |  |  | 0.19 |
| Os.136.1.S1_a_at | bZIP family |  | 6.12 |  |  |  |  |
| Os.26437.1.A1_s_at | bZIP family |  | 0.04 |  |  |  |  |
| OsAffx.23844.1.S1_s_at | bZIP family |  | 0.15 |  |  |  |  |
| Os.26437.1.A1_at | bZIP family |  | 0.14 |  |  |  |  |
| Os.26491.1.S1_at | bZIP family |  | 0.09 |  |  |  |  |
| Os.8561.1.S1_at | bZIP family |  | 0.18 |  |  |  |  |
| Os.18354.1.S1_at | bZIP family |  |  |  |  | 5.95 |  |
| OsAffx.2611.1.S1_at | bZIP family |  |  |  | 0.15 |  |  |
| Os.49294.1.S1_at | C2C2-CO-like family |  |  |  |  |  | 6.91 |
| Os.1189.1.S1_at | C2C2-CO-like family |  | 7.71 |  |  |  |  |
| Os.57511.1.A1_at | C2C2-CO-like family |  | 0.15 |  |  |  |  |
| Os.38848.1.S1_at | C2C2-CO-like family |  | 0.07 |  |  |  |  |
| Os.13999.1.S1_at | C2C2-CO-like family |  |  |  | 9.15 |  |  |
| Os.7920.1.S1_at | C2C2-CO-like family |  |  |  | 7.89 |  |  |
| Os.4185.1.A1_at | C2C2-Dof family |  | 0.13 |  | 0.18 | 0.15 |  |
| Os.4186.1.S1_a_at | C2C2-Dof family |  | 22.06 | 7.30 |  |  |  |
| Os.7834.1.S1_at | C2C2-GATA family | 0.14 |  | 0.13 |  |  |  |
| Os.53733.1.S1_at | C2C2-GATA family |  | 0.07 |  |  |  |  |
| Os.21239.1.S1_at | C2C2-GATA family | 0.13 |  |  |  |  |  |
| Os.28441.1.S1_at | C2H2 family |  | 38.81 | 18.04 |  |  |  |
| Os.16453.1.S1_at | C2H2 family |  | 42.46 | 6.63 |  |  |  |
| Os.15874.1.A1_at | C2H2 family |  | 63.91 | 6.78 |  |  |  |
| Os.10411.1.S1_at | C2H2 family |  | 77.27 | 10.23 |  |  |  |
| Os.49588.1.S1_at | C2H2 family |  | 11.78 |  |  |  |  |
| Os.36152.1.S1_at | C2H2 family |  | 11.12 |  |  |  |  |
| Os.9450.1.S1_at | C2H2 family |  | 5.52 |  |  |  |  |
| Os.54232.1.S1_at | C2H2 family | 6.87 |  |  |  |  |  |
| Os.51307.1.S1_at | C2H2 family |  |  |  | 0.09 | 0.14 |  |
| Os.54858.1.S1_at | C2H2 family |  |  |  |  |  | 0.20 |
| OsAffx.17906.1.S1_x_at | C2H2 family |  | 0.18 |  |  |  |  |
| Os.57344.1.S1_at | C2H2 family |  |  |  | 0.13 |  |  |
| Os.55911.1.S1_at | C2H2 family |  |  |  | 0.20 |  |  |
| Os.28023.1.S1_at | C2H2 family |  |  |  | 0.07 |  |  |
| Os.31975.1.S1_x_at | C3H family |  | 7.28 | 6.92 |  |  | 8.20 |
| Os.29084.1.S1_at | C3H family |  | 40.71 |  | 15.48 | 53.86 |  |
| Os.6318.1.S1_at | C3H family |  | 11.54 |  |  |  |  |
| Os.54644.1.S1_at | C3H family |  |  |  |  |  | 0.19 |
| Os.4796.1.S1_at | C3H family |  | 0.18 |  |  |  |  |
| Os.7928.1.S1_at | CCAAT-HAP2 family |  |  | 6.21 |  |  |  |
| Os.49770.1.S1_s_at | CCAAT-HAP3 family |  | 0.14 | 0.18 |  |  |  |
| Os.49795.1.S1_at | CCAAT-HAP3 family |  | 0.12 |  |  |  |  |
| Os.12037.1.S1_at | CCAAT-HAP5 family |  | 0.13 |  |  |  |  |
| Os.10524.2.S1_at | FHA family |  | 0.09 | 0.10 |  |  |  |
| Os.8564.1.S1_at | FHA family |  | 0.07 | 0.13 |  |  |  |
| Os.16055.1.S1_at | FHA family |  | 13.52 |  |  |  |  |
| Os.7512.1.S1_at | GARP-G2-like family |  | 0.17 |  |  | 0.12 |  |
| Os.55501.1.S1_at | GARP-G2-like family |  | 25.25 |  |  |  |  |
| Os.53136.1.S1_at | GARP-G2-like family |  | 10.03 |  |  |  |  |
| Os.52219.1.A1_at | GARP-G2-like family |  | 7.28 |  |  |  |  |
| Os.24699.1.S1_at | GARP-G2-like family |  | 0.09 |  |  |  |  |
| Os.31716.2.A1_at | GRAS family | 0.18 | 0.04 | 0.08 |  |  |  |
| Os.21578.1.S1_at | GRAS family |  | 0.13 |  |  |  |  |
| Os.2406.2.S1_a_at | GRAS family |  | 0.17 |  |  |  |  |
| Os.49702.1.S1_at | GRAS family |  |  |  |  | 0.12 |  |
| Os.53020.1.S1_at | GRAS family | 0.08 |  |  |  |  |  |
| Os.49245.1.S1_at | HB family | 9.76 | 106.02 | 67.21 | 16.15 | 9.90 | 24.51 |
| Os.12134.1.S1_at | HB family |  |  | 24.59 | 26.85 | 13.53 | 12.00 |
| Os.2362.2.S1_x_at | HB family | 13.45 | 33.93 | 23.02 |  |  | 5.37 |
| OsAffx.11118.1.S1_s_at | HB family |  | 14.14 | 5.68 | 5.29 | 8.49 |  |
| OsAffx.29958.1.S1_x_at | HB family |  | 10.42 | 5.01 |  |  | 34.77 |
| Os.9086.1.S1_at | HB family |  | 33.51 | 8.14 |  |  | 16.60 |
| Os.17301.1.S1_at | HB family |  | 0.06 |  |  |  | 0.19 |
| Os.9332.1.S1_at | HB family | 0.17 | 0.06 |  |  |  |  |
| Os.6271.1.S1_at | HB family |  |  |  |  |  | 10.78 |
| Os.37618.1.S1_at | HB family |  |  |  |  |  | 0.07 |
| Os.2365.1.S1_at | HB family |  |  |  |  |  | 0.11 |
| Os.2364.1.S1_at | HB family |  | 0.12 |  |  |  |  |
| Os.8833.1.S1_at | HB family | 6.31 |  |  |  |  |  |
| Os.54219.1.S1_at | HMG family | 5.03 | 8.49 |  | 16.28 |  |  |
| Os.57443.1.S1_x_at | HMG family |  | 0.14 |  |  |  |  |
| Os.10570.1.S1_at | HSF family | 87.86 | 97.31 | 49.70 | 55.08 |  | 9.47 |
| Os.11250.1.S1_at | HSF family | 9.86 |  | 26.00 | 14.02 | 7.96 | 10.70 |
| Os.51916.1.S1_at | HSF family |  | 32.27 | 7.41 | 16.77 | 49.04 |  |
| Os.35681.1.S1_at | HSF family | 5.03 | 5.10 |  | 19.22 | 12.23 |  |
| Os.2292.3.S1_x_at | HSF family |  | 6.18 |  |  | 19.67 |  |
| Os.23778.1.S1_at | HSF family |  |  |  | 6.96 | 6.90 |  |
| Os.10942.1.S1_a_at | HSF family |  |  |  |  | 13.34 |  |
| Os.2292.2.S1_at | HSF family |  |  |  |  | 38.09 |  |
| OsAffx.27969.1.S1_x_at | HSF family |  |  |  |  | 6.69 |  |
| OsAffx.30145.1.S1_at | HSF family |  |  |  |  | 5.22 |  |
| Os.40021.1.S1_a_at | HSF family |  | 0.18 |  |  |  |  |
| Os.7784.1.S1_at | LIM family |  |  |  |  |  | 0.20 |
| Os.49837.1.S1_a_at | MADS family |  | 0.19 |  |  |  |  |
| Os.11046.1.S1_at | MBF1 family |  | 6.45 |  | 0.15 |  |  |
| Os.2867.1.A1_at | MYB family | 6.39 | 22.34 | 17.75 |  |  | 7.34 |
| Os.56210.1.S1_at | MYB family | 6.35 |  | 6.41 | 15.55 | 16.65 |  |
| Os.32634.1.S1_at | MYB family |  | 48.73 | 9.76 |  |  |  |
| Os.20224.1.S1_at | MYB family |  | 90.84 | 12.89 |  |  |  |
| Os.55096.1.S1_at | MYB family |  |  |  | 15.39 | 33.49 |  |
| OsAffx.3135.1.S1_at | MYB family |  |  |  | 9.30 | 12.97 |  |
| Os.49829.1.S1_at | MYB family |  |  |  | 6.37 | 8.32 |  |
| Os.3390.1.S1_at | MYB family |  | 19.28 |  |  |  |  |
| Os.3388.2.S1_a_at | MYB family |  | 12.77 |  |  |  |  |
| Os.54934.1.S1_at | MYB family |  | 6.82 |  |  |  |  |
| Os.31381.1.S1_at | MYB family |  | 8.61 |  |  |  |  |
| Os.3387.1.S1_at | MYB family |  | 5.59 |  |  |  |  |
| Os.9336.1.S1_at | MYB family |  | 8.09 |  |  |  |  |
| Os.9971.1.S1_s_at | MYB family |  | 6.84 |  |  |  |  |
| Os.9514.1.S1_at | MYB family | 0.11 |  |  | 0.17 |  |  |
| Os.49848.1.S1_at | MYB family | 0.13 |  |  | 0.14 |  |  |
| Os.10115.1.S1_at | MYB family | 0.08 |  |  |  |  |  |
| Os.21636.2.S1_a_at | MYB family | 0.17 |  |  |  |  |  |
| Os.3391.1.S1_at | MYB family |  |  |  |  |  | 0.16 |
| Os.47854.1.S1_at | MYB family |  |  |  |  |  | 0.17 |
| OsAffx.2312.1.S1_at | MYB family |  |  |  | 0.16 |  |  |
| Os.49830.1.S1_at | MYB family |  |  |  | 0.17 |  |  |
| Os.10172.1.S1_at | MYB family |  |  |  | 0.12 |  |  |
| Os.3386.1.S1_x_at | MYB family |  | 0.04 |  |  |  |  |
| OsAffx.15178.1.S1_s_at | MYB-related family | 0.11 | 0.01 | 0.02 | 0.06 |  |  |
| Os.41164.1.S1_at | MYB-related family | 0.17 | 0.02 | 0.04 | 0.11 |  |  |
| Os.49787.1.S1_at | MYB-related family |  | 5.29 | 15.44 |  |  | 10.01 |
| Os.623.3.S1_x_at | MYB-related family |  | 7.98 | 7.51 |  |  |  |
| Os.5335.1.S1_at | MYB-related family |  | 50.19 | 18.33 |  |  |  |
| Os.3141.1.S1_at | MYB-related family |  | 0.15 |  |  |  |  |
| OsAffx.5446.1.S1_s_at | MYB-related family |  | 0.04 |  |  |  |  |
| OsAffx.2971.1.S1_at | MYB-related family |  | 0.12 |  |  |  |  |
| Os.35196.1.S1_at | MYB-related family | 0.10 |  |  |  |  |  |
| Os.8149.1.S1_at | MYB-related family |  |  |  | 14.68 |  |  |
| Os.18955.1.S1_at | MYB-related family |  |  |  | 5.00 |  |  |
| Os.37548.1.S1_at | NAC family | 13.54 | 11.91 | 6.49 | 10.17 | 10.56 |  |
| Os.35020.1.S1_at | NAC family | 21.32 | 18.82 | 28.22 |  |  | 45.04 |
| Os.47735.1.S1_at | NAC family | 8.88 | 19.77 | 8.37 |  |  | 5.48 |
| Os.17286.1.S1_at | NAC family | 9.11 | 51.80 | 15.93 |  |  |  |
| Os.802.1.S1_at | NAC family |  | 9.38 |  | 0.17 | 0.18 |  |
| Os.53950.1.S1_at | NAC family | 0.14 |  |  | 0.20 | 0.11 |  |
| Os.53228.1.S1_at | NAC family | 12.83 |  | 18.27 |  |  |  |
| Os.26695.1.S1_at | NAC family |  |  | 22.27 |  |  | 13.14 |
| Os.56943.1.S1_at | NAC family |  | 0.11 | 0.08 |  |  |  |
| Os.4384.1.S1_at | NAC family |  | 6.05 |  |  |  | 9.11 |
| Os.36651.1.S1_at | NAC family |  |  |  | 0.03 | 0.09 |  |
| Os.4385.1.S1_at | NAC family |  |  | 7.50 |  |  |  |
| Os.51762.1.S1_at | NAC family |  |  | 5.55 |  |  |  |
| Os.35343.1.A1_at | NAC family |  |  | 0.16 |  |  |  |
| Os.7362.1.S1_at | NAC family |  |  |  |  |  | 7.40 |
| Os.18595.1.A1_at | NAC family |  | 14.71 |  |  |  |  |
| Os.39872.1.A1_s_at | NAC family |  | 15.79 |  |  |  |  |
| Os.9354.2.S1_at | NAC family |  | 7.28 |  |  |  |  |
| Os.15708.1.S1_a_at | NAC family |  | 6.52 |  |  |  |  |
| Os.32252.1.S1_at | NAC family |  | 11.36 |  |  |  |  |
| Os.7235.1.S1_at | NAC family |  | 10.30 |  |  |  |  |
| Os.26957.1.A1_a_at | NAC family |  | 7.39 |  |  |  |  |
| Os.17090.1.S1_at | NAC family |  | 0.14 |  |  |  |  |
| Os.34520.1.S1_at | NAC family |  | 0.06 |  |  |  |  |
| Os.23030.1.S1_at | NAC family |  | 0.19 |  |  |  |  |
| Os.39129.1.S1_at | NAC family |  |  |  | 0.10 |  |  |
| Os.51120.1.S1_at | NAC family |  |  |  | 0.11 |  |  |
| OsAffx.30176.1.S1_at | Nin-like family |  | 0.18 |  |  |  |  |
| Os.27449.1.S1_at | PcG family |  | 0.14 |  |  |  |  |
| OsAffx.6386.1.S1_at | PcG family |  | 0.06 |  |  |  |  |
| Os.57573.1.S1_at | PHD family |  | 23.59 | 7.43 |  |  |  |
| Os.54501.1.S1_at | PHD family |  | 15.61 |  |  |  |  |
| Os.49723.1.S1_at | PHD family |  | 6.93 |  |  |  |  |
| Os.18134.1.S1_at | PHD family |  | 0.10 |  |  |  |  |
| Os.49509.1.S1_at | PLATZ family |  |  |  |  |  | 0.13 |
| Os.52576.1.S1_at | SBP family |  | 0.13 |  |  |  |  |
| Os.9500.1.S1_s_at | SBP family |  | 0.19 |  |  |  |  |
| Os.11215.1.S1_a_at | SBP family |  | 0.16 |  |  |  |  |
| Os.26472.1.S1_at | TAZ family |  | 0.20 |  | 0.13 |  |  |
| Os.52097.1.S1_at | TCP family |  |  |  |  |  | 0.05 |
| Os.189.1.S1_at | TCP family |  | 0.07 |  |  |  |  |
| Os.5286.1.S1_at | TCP family |  | 0.06 |  |  |  |  |
| Os.5515.1.S1_at | Trihelix family |  | 5.93 |  |  |  |  |
| Os.13466.1.S1_at | Trihelix family |  | 5.18 |  |  |  |  |
| Os.6016.1.A1_s_at | Trihelix family |  | 0.20 |  |  |  |  |
| Os.33990.1.S1_at | WRKY family |  | 6.12 | 5.04 |  |  |  |
| Os.33131.1.A1_at | WRKY family |  | 24.25 | 6.36 |  |  |  |
| Os.55827.1.S1_at | WRKY family |  | 56.34 | 8.24 |  |  |  |
| Os.37565.2.S1_at | WRKY family |  | 0.11 | 0.15 |  |  |  |
| Os.25606.1.S1_at | WRKY family |  | 0.15 |  | 0.18 |  |  |
| OsAffx.30783.1.S1_at | WRKY family |  | 0.15 |  | 0.12 |  |  |
| Os.48082.1.S1_at | WRKY family |  | 0.10 |  | 0.20 |  |  |
| OsAffx.30442.1.S1_at | WRKY family |  |  |  | 0.02 | 0.09 |  |
| Os.11773.1.S1_at | WRKY family |  |  |  |  |  | 5.69 |
| Os.30386.1.S1_at | WRKY family |  | 20.46 |  |  |  |  |
| OsAffx.16444.1.S1_at | WRKY family |  | 6.26 |  |  |  |  |
| Os.14882.1.S1_at | WRKY family |  | 0.19 |  |  |  |  |
| Os.11321.1.S1_at | WRKY family |  |  |  |  | 0.09 |  |
| Os.11945.1.S1_at | WRKY family |  |  |  | 0.16 |  |  |
| Os.50015.1.S1_at | WRKY family |  |  |  | 0.14 |  |  |
| Os.50830.1.S1_at | ZF-HD family |  |  |  |  |  | 0.20 |
| Os.57347.1.S1_at | ZF-HD family |  | 0.16 |  |  |  |  |
| OsAffx.29500.1.S1_x_at | ZF-HD family | 0.18 |  |  |  |  |  |
| Os.46849.1.S1_at | ZIM family |  | 6.77 |  | 0.07 | 0.07 |  |
| Os.10356.1.S1_at | ZIM family |  | 8.58 | 25.59 |  |  |  |
| Os.12012.1.S1_at | ZIM family |  |  | 6.69 |  |  |  |
| Os.9923.1.S1_s_at | ZIM family |  |  | 5.26 |  |  |  |
| Os.8088.1.S1_at | ZIM family |  |  |  |  | 0.16 |  |
