# Supplementary material for: Genome-wide temporal-spatial gene expression profiling of drought responsiveness in rice
Source: BMC Genomics. 2011 Mar 16;12:149. doi: 10.1186/1471-2164-12-149 (PMC3070656; doi:10.1186/1471-2164-12-149)
Supplement: Additional file 17 — List of TF gene stages specifically regulated by drought stress. Excel file of the identified transcription factor genes specifically regulated by drought stress [file 1471-2164-12-149-S17.DOC]

**Additional file 17**. The list of TF genes stage specific-regulated by drought stress

| **Probe ID** | **TF** | **TL** | **PL** | **BL** | **TR** | **PR** | **BP** |
| --- | --- | --- | --- | --- | --- | --- | --- |
| Tillering Stage |  |  |  |  |  |  |  |
| Os.1503.1.S1_at | bZIP family | 0.18 |  |  | 0.05 |  |  |
| Os.9514.1.S1_at | MYB family | 0.11 |  |  | 0.17 |  |  |
| Os.49848.1.S1_at | MYB family | 0.13 |  |  | 0.14 |  |  |
| Panicle Elongation Stage | |  |  |  |  |  |  |
| Os.6043.1.S1_at | bHLH family | 11.46 | 0.06 |  |  | 0.17 |  |
| Os.7512.1.S1_at | GARP-G2-like family |  | 0.17 |  |  | 0.12 |  |
| Os.3390.1.S1_at | MYB family |  | 19.28 |  |  |  |  |
| Os.3388.2.S1_a_at | MYB family |  | 12.77 |  |  |  |  |
| Os.54934.1.S1_at | MYB family |  | 6.82 |  |  |  |  |
| Os.31381.1.S1_at | MYB family |  | 8.61 |  |  |  |  |
| Os.3387.1.S1_at | MYB family |  | 5.59 |  |  |  |  |
| Os.9336.1.S1_at | MYB family |  | 8.09 |  |  |  |  |
| Os.9971.1.S1_s_at | MYB family |  | 6.84 |  |  |  |  |
| Os.3386.1.S1_x_at | MYB family |  | 0.04 |  |  |  |  |
| Os.3141.1.S1_at | MYB-related family |  | 0.15 |  |  |  |  |
| OsAffx.5446.1.S1_s_at | MYB-related family |  | 0.04 |  |  |  |  |
| OsAffx.2971.1.S1_at | MYB-related family |  | 0.12 |  |  |  |  |
| Os.802.1.S1_at | NAC family |  | 9.38 |  | 0.17 | 0.18 |  |
| Os.18595.1.A1_at | NAC family |  | 14.71 |  |  |  |  |
| Os.39872.1.A1_s_at | NAC family |  | 15.79 |  |  |  |  |
| Os.9354.2.S1_at | NAC family |  | 7.28 |  |  |  |  |
| Os.15708.1.S1_a_at | NAC family |  | 6.52 |  |  |  |  |
| Os.32252.1.S1_at | NAC family |  | 11.36 |  |  |  |  |
| Os.7235.1.S1_at | NAC family |  | 10.30 |  |  |  |  |
| Os.26957.1.A1_a_at | NAC family |  | 7.39 |  |  |  |  |
| Os.17090.1.S1_at | NAC family |  | 0.14 |  |  |  |  |
| Os.34520.1.S1_at | NAC family |  | 0.06 |  |  |  |  |
| Os.23030.1.S1_at | NAC family |  | 0.19 |  |  |  |  |
| Os.46849.1.S1_at | ZIM family |  | 6.77 |  | 0.07 | 0.07 |  |
| Booting Stage |  |  |  |  |  |  |  |
| Os.51063.1.S1_at | bHLH family |  |  | 21.14 |  |  | 5.94 |
| Os.413.1.S1_at | bZIP family |  |  | 15.12 |  |  | 8.19 |
| Os.26695.1.S1_at | NAC family |  |  | 22.27 |  |  | 13.14 |
| Os.4385.1.S1_at | NAC family |  |  | 7.50 |  |  |  |
| Os.51762.1.S1_at | NAC family |  |  | 5.55 |  |  |  |
| Os.35343.1.A1_at | NAC family |  |  | 0.16 |  |  |  |
| Os.52097.1.S1_at | TCP family |  |  |  |  |  | 0.05 |
| Os.11773.1.S1_at | WRKY family |  |  |  |  |  | 5.69 |
| Os.50830.1.S1_at | ZF-HD family |  |  |  |  |  | 0.20 |
