# Supplementary figures and images for: Genome-wide temporal-spatial gene expression profiling of drought responsiveness in rice
Source: BMC Genomics. 2011 Mar 16;12:149. doi: 10.1186/1471-2164-12-149 (PMC3070656; doi:10.1186/1471-2164-12-149)

## Slide 1
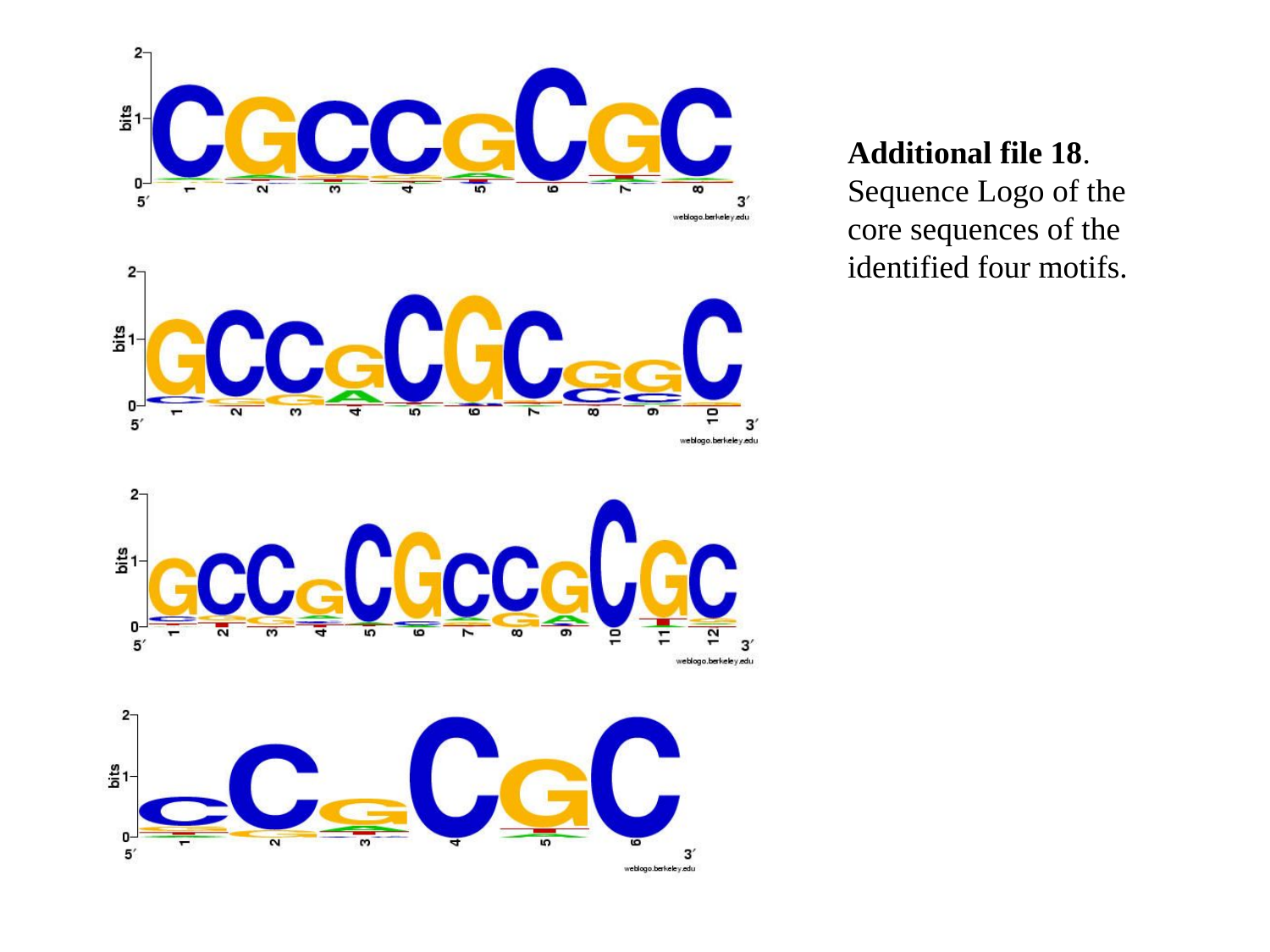

Additional file 18. Sequence Logo of the core sequences of the identified four motifs.

Supplement: Additional file 18 — Sequence Logo of the core sequences of the identified four motifs. A PPT file of the sequence logo of the core sequences of the identified motifs [file 1471-2164-12-149-S18.PPT]
